# Tuning the Supramolecular Polymerization and Cell Response of Ureidopyrimidinone Monomers by Pushing the Hydrophobic Threshold

**DOI:** 10.1021/jacs.5c01445

**Published:** 2025-06-11

**Authors:** Riccardo Bellan, Martin G. T. A. Rutten, Xianwen Lou, Chloe M. Wallace, Maritza M. Rovers, Andrew J. Smith, Marcel H. P. van Genderen, Dave J. Adams, Patricia Y. W. Dankers

**Affiliations:** 1 Institute for Complex Molecular Systems, 3169 Eindhoven University of Technology, Eindhoven 5600 MB, The Netherlands; 2 Department of Biomedical Engineering, 3169 Eindhoven University of Technology, Eindhoven 5600 MB, The Netherlands; 3 Department of Chemical Engineering and Chemistry, 3169 Eindhoven University of Technology, Eindhoven 5600 MB, The Netherlands; 4 School of Chemistry, 3526 University of Glasgow, Glasgow, Scotland G12 8QQ, U.K.; 5 120796 Diamond Light Source Ltd., Diamond House, Harwell Science and Innovation Campus, Didcot OX11 0DE, U.K.

## Abstract

Understanding the assembly of small molecules in aqueous media is crucial for the development of adaptive biomaterials. The mechanical properties of supramolecular networks, including stiffness and stress relaxation, play a key role in cellular spreading and can be tuned via formulation strategies or monomer design. Here, we demonstrate the modulation of supramolecular polymerization and cellular response of ureidopyrimidinone (UPy) monomers in water by tailoring the length of the alkyl spacer within the monomer structure. A library of four UPy derivatives with varying hydrophilic–hydrophobic balances was synthesized by using an optimized synthetic approach. The assembly behavior and dynamics of the supramolecular polymers were investigated both in solution and gel states using a wide range of techniques. The results revealed that the alkyl spacer length significantly affects the supramolecular polymer dynamics, kinetics, and stability. Monomers with 6 and 8 methylene units formed dynamic elongated structures, while those with 10 and 12 units yielded robust and stable bundled fibers. In the gel state, a physical cross-linker was required for gel formation. The gels formed by the monomers featuring 8 and 10 methylene units exhibited optimal mechanical properties, promoting the spreading of human normal dermal fibroblasts in both 2D and 3D cultures. These findings highlight the impact of the monomer architecture on the properties of UPy supramolecular systems, paving the way for the rational design of biomaterials with tunable properties.

## Graphical abstract

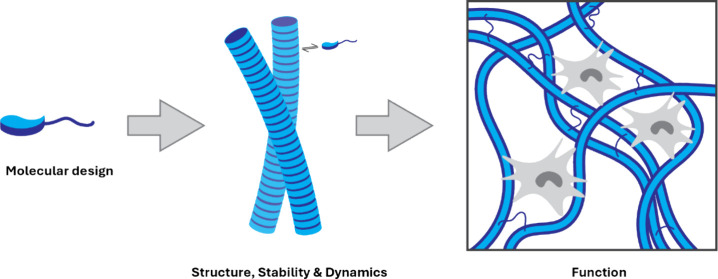

## Introduction

Supramolecular polymers are ubiquitous in living systems, and they regulate both functional and structural aspects of cellular life, such as cell division and cell motility.^1–3^ The noncovalent nature of the interactions involved in their assembly process confers to them the unique ability of being robust yet dynamic.^4^ As a consequence of the aforementioned properties, natural supramolecular polymers have inspired scientists to develop synthetic supramolecular polymers that are responsive to external stimuli, thus providing a class of materials with excellent biocompatibility and therefore suitable for biomedical applications.^5^ Indeed, when dispersed in water, under appropriate conditions, these polymers can entangle and trap solvent molecules through surface tension and capillary forces, leading to the formation of three-dimensional (3D) networks with viscoelastic properties^6^ that can be employed as delivery systems and/or as three-dimensional scaffolds for regenerative medicine.^7,8^ Furthermore, supramolecular biomaterials present modularity and tunability due to the noncovalent interactions between monomers that allows the incorporation of bioactive cues.^9^

Peptide amphiphiles (PAs) represent a class of self-assembling monomers consisting of an alkyl tail covalently attached to a peptide sequence that can self-assemble into nanofibers in water through a combination of hydrophobic interactions and hydrogen bonding.^10–12^ Notably, PAs have been shown to effectively stabilize growth factors and to induce bone and nerve regeneration by means of a modular approach.^13–15^ Furthermore, the tuning of the chemical structure within the peptide sequence was demonstrated to affect the rigidity of the supramolecular structures, which, in turn, results in different biological responses. Indeed, several examples are highlighting how the bioactivity of the supramolecular constructs is enhanced by the high degree of supramolecular motion,^16–18^ probably due to a more frequent interaction of the bioactive ligand with the receptor, as observed for disordered proteins in biological systems.^19^

Another example of synthetic self-assembling building blocks is the 1,3,5-benzene-tricarboxamide (BTA) molecules, which assemble into μm-long one-dimensional (1D) dynamic fibers in water through a combination of 3-fold hydrogen bonding between the amides, π–π stacking, and hydrophobic interactions.^20–23^ BTA-based fibers with bioactive groups have been studied for intracellular delivery,^24^ protein recruitment,^25^ and anchoring to red blood cells.^26^ Furthermore, BTA-based hydrogels were shown to have potential as 3D printable bioinks.^27,28^ However, their high dynamicity limits their effectiveness in tissue engineering, as it hinders cell spreading.^29^

In comparison, urea motifs form stronger interactions than amides owing to multiple and cooperative hydrogen bonding.^30^ To this end, water-compatible bis-urea bolaamphiphiles were developed and were found to form supramolecular structures whose morphology depends on the hydrophilic/hydrophobic balance of the supramolecular monomer.^31,32^ These structures can be formulated into hydrogels with bioactivity, injectability, and strain-stiffening properties.^33–35^

Ureidopyrimidinone (UPy) molecules are another class of self-assembling monomers whose assembly partially relies on urea motifs. Indeed, UPy hydrogelators usually consist of a hard block composed of a UPy core and a C_6_ alkyl chain connected to a second C_12_ chain via a urea group. This design allows the dimerization of the UPy motifs in water via quadruple hydrogen bonding and the formation of 1D supramolecular fibers through a combination of π–π stacking and hydrophobic interactions, which are further stabilized by the lateral hydrogen bonding provided by the urea groups.^36,37^ The hard block is then connected to a poly­(ethylene glycol) (PEG) chain via a urethane group to ensure the water solubility of the assemblies.^38^ UPy fibers decorated with cationic bioactive additives in solution were shown to promote intracellular siRNA delivery^39^ and to stabilize growth factors.^40^ Furthermore, by increasing the fiber concentration and by adding a bifunctional telechelic UPy cross-linker (BF), UPy hydrogels can be obtained^38,41^ and used as pH-responsive injectable delivery systems^42–45^ as well as 3D scaffolds for regenerative medicine as synthetic ECM mimics^46–48^ due to their tunable stiffness, stress relaxation, and slow fiber dynamics.^29^ Indeed, the dynamicity of the UPy fibers was investigated both in solution^49^ and in bulk^48^ by mixing a monovalent UPy with a BF, showing that an increasing amount of BF leads to an increase in fiber dynamicity in both cases as well as lower cell adhesion and spreading within the gels.^48^

The structure–assembly relationship in water has been well-established for PAs,^50–52^ BTAs,^21,23,53^ and bis-urea bolaamphiphiles,^32^ highlighting the importance of the hydrophilic–hydrophobic balance in determining the nanostructure morphology, dynamics, and bioactivity. Although many studies provided insights into the structure–assembly relationship of UPy molecules in organic solvents,^36,37,54^ this relationship remains largely unexplored in an aqueous environment. Indeed, only the effect of varying the PEG length on their assembly process was reported.^38,41^

Hence, this work aims to establish a structure–assembly relationship for UPy molecules in aqueous environment to find a hydrophobic–hydrophilic threshold that leads to supramolecular polymers with similar properties to those of the UPy molecules reported above, both in solution and in the gel state, while improving their water solubility. To this end, four novel UPy derivatives were synthesized, varying the length of the alkyl spacer between the urea group and the undeca­(ethylene glycol) methyl ether (mOEG_11_) chain. Furthermore, the urethane group connecting the hard block to the mOEG_11_ chain is replaced by an ether moiety, as for BTAs,^27,53^ through a facile synthetic procedure. A detailed examination of the self-assembly behavior of the UPy monomers in aqueous solution at different pHs is performed using fluorescence, UV–vis and ^1^H NMR spectroscopy, small-angle X-ray scattering (SAXS), and cryogenic transmission electron microscopy (cryoTEM). In addition, the dynamics of the supramolecular systems is addressed through hydrogen/deuterium exchange followed by mass spectrometry (HDX-MS).^55^ Moreover, the UPy materials were formulated into hydrogels by mixing them with a small amount of BF at different ratios, and the resulting rheological properties were analyzed using shear rheology. Lastly, the effects of varying the number of methylene units in the alkyl spacer on the cell adhesion and spreading were tested on fibroblast cells.

## Results and Discussion

### Design and Synthesis

The monomer design takes inspiration from the work of Kieltyka et al.^38^ where the assembly behavior of a UPy molecule composed by a UPy core followed by a C_6_ spacer, a urea group, and a C_12_ spacer connected to a mOEG_11_ via a urethane group was investigated. In the current work, four novel UPy molecules (**Cn-O**) are synthesized through a facile method where the urethane group is replaced by an ether bond and the alkyl spacer connecting the mOEG_11_ chain consists of 6 (**C6**-O****), 8 (**C8**-O****), 10 (**C10**-O****), or 12 (**C12**-O****) methylene units. The synthesis of the final molecules can be summarized as the coupling of amphiphilic amines **7a**–**d** to the isocyanate group of UPy synthon **2** as described in Scheme 1.

**Scheme 1:**
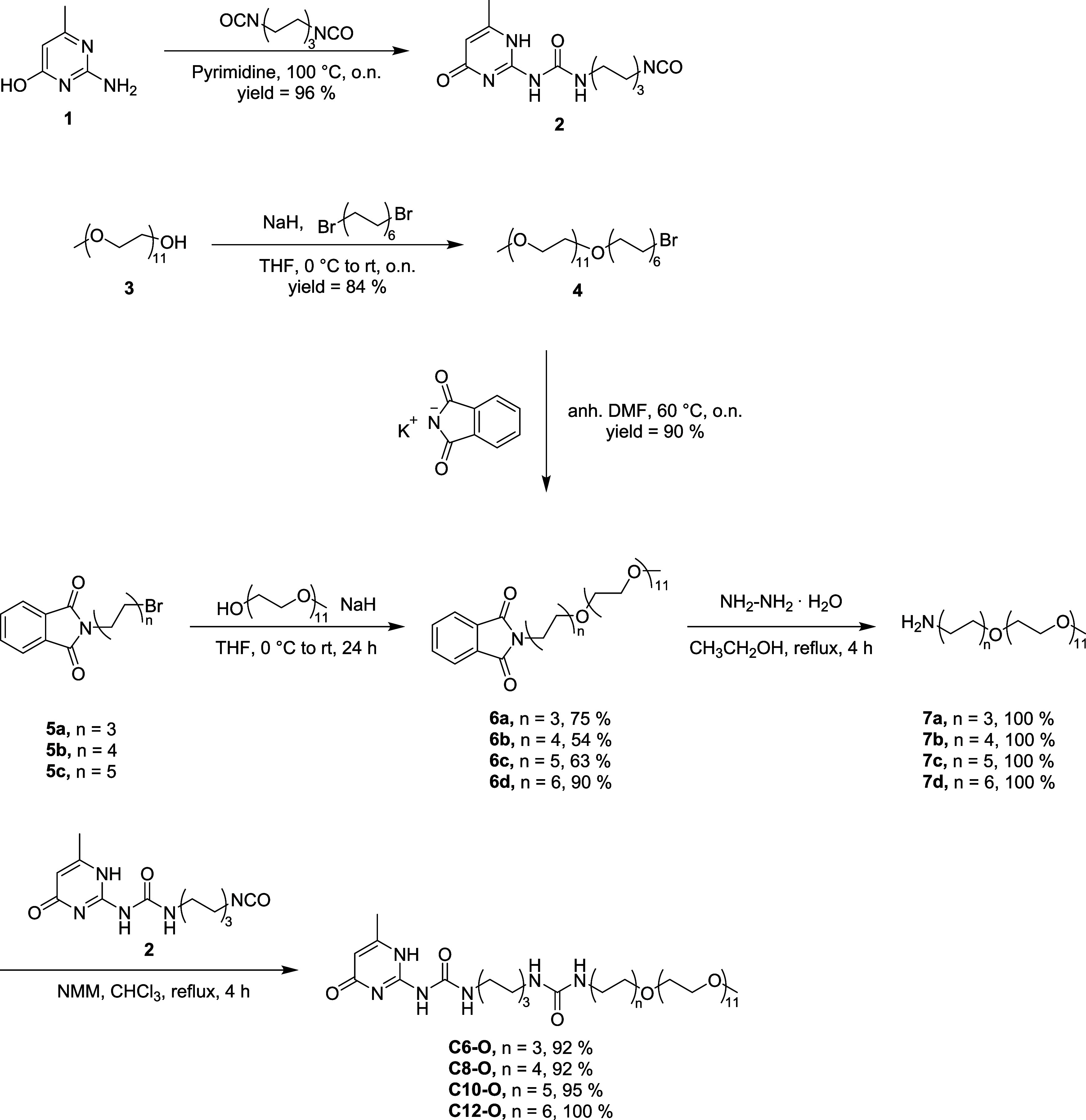
Synthetic routes toward the final UPy materials employed in this study

A key point for the synthesis of **Cn-O** involves the formation of an ether bond between mOEG_11_–OH (**3**) and the respective alkyl chains through Williamson etherification.^56^ In particular, for the synthesis of **6a**–**c**, mOEG_11_–OH is converted into an alkoxide by sodium hydride and reacted with the respective *N*-(bromoalkyl)­phthalimide (**5a**–**c**) to provide **6a**–**c** in good yields (63–75%). For **6d**, the lack of commercial availability of *N*-(12-bromododecyl)­phthalimide required its synthesis by reacting **3** with 1,12,-dibromododecane, followed by the nucleophilic substitution (S_N_2) of the bromide of **4** with potassium phthalimide to provide **6d** in two total steps as previously described.^57^ Next, the phthalimide intermediates (**6a**–**d**) are converted into the respective amines (**7a**–**d**) through Gabriel synthesis^58^ in the presence of hydrazine hydrate and then reacted with the UPy synthon **2** to provide the final compounds in nearly quantitative yields. The synthesized monomers are obtained as white solids after freeze-drying and are fully characterized by means of ^1^H NMR, ^13^C NMR, and liquid chromatography mass spectrometry (LC-MS).

### Self-Assembly in Solution

Once obtained, all the monomers are assembled in water at neutral pH and their assembly is quantified by the Nile red fluorescence (NRF) assay. Nile red is a solvatochromic fluorescent dye that exhibits a blueshift upon encapsulation in a hydrophobic environment,^59^ therefore allowing the determination of the critical aggregation concentration (cac) of supramolecular polymers.^60^ The concentration-dependent Nile red curves (Figure 1a) of the assembled monomers in solution highlight the importance of the hydrophobic chain length in driving their assembly process by displaying increasing cac values with a decreasing number of methylene units. Indeed, the most hydrophobic **C12**-O**** presents a cac of 1.0 μM, which increases to 5.0 μM for **C10**-O**** with two fewer methylene units. Similarly, **C6**-O**** and **C8**-O**** exhibit comparable cac values between 75 and 80 μM due to the smaller size of the respective alkyl chains.

**Figure 1:**
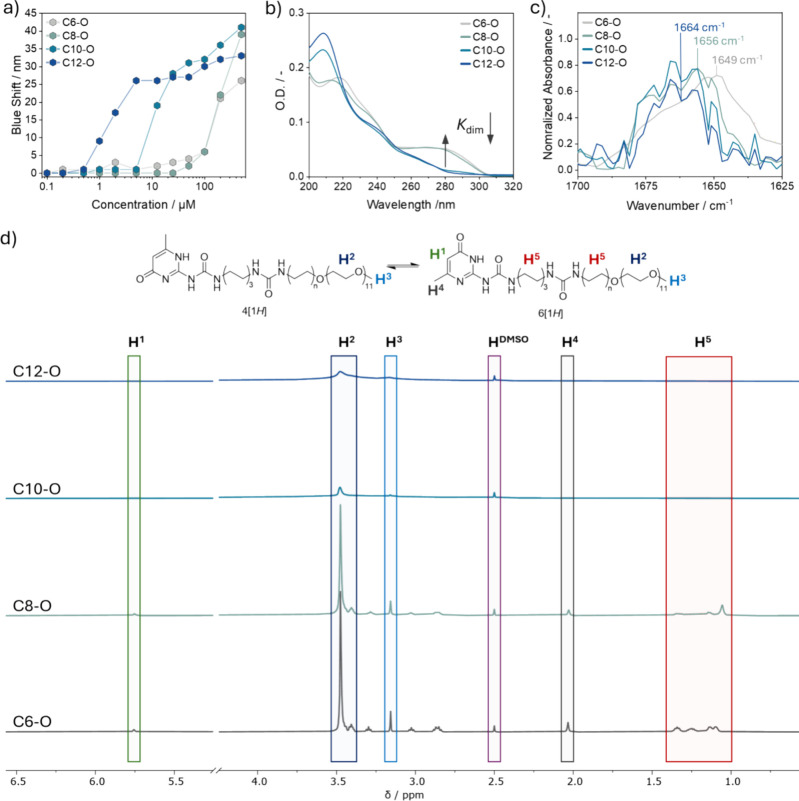
Self-assembly analyses of the synthesized molecules in MQ water. (a) Nile red fluorescence assay for the determination of the cac of the UPy molecules (*C* = 0.1–500 μM, *T* = 20 °C). (b) UV–vis spectra of the synthesized molecules (*C* = 100 μM, *T* = 20 °C, and *l* = 1 mm). (c) Partial FT-IR spectrum of the amide I vibration of each monomer in D_2_O (*C* = 2.5 mM, *T* = 20 °C, and *l* = 0.05 mm). (d) ^1^H NMR spectra (500 MHz, D_2_O, 298 K) of the synthesized molecules at room temperature (*C* = 500 μM).

To obtain more information on the assembly of the synthesized molecules, UV–vis spectroscopy in combination with Fourier transform infrared (FT-IR) and ^1^H NMR spectroscopy was performed above the cac of each monomer (Figure 1b–d). The UV–vis spectra of **C12**-O**** and **C10**-O**** (Figure 1b) at 100 μM look similar, presenting an absorption maximum at 208 nm and two shoulders at 240 and 260 nm corresponding to the hydrogen-bonded UPy 4­[1*H*] tautomer.^54^ On the other hand, the UV–vis spectra of **C6**-O**** and **C8**-O**** at the same concentration (Figure 1b) present maxima at 218 and 214 nm, respectively, a shoulder at 240 nm and a second maximum at 280 nm for **C6**-O**** and at 275 nm for **C8**-O****. The higher absorption at 280 nm corresponds to the nonhydrogen-bonded 6­[1*H*] tautomer and to a decreased *K*_dim_ for **C6**-O**** and **C8**-O**** compared to those of the more hydrophobic derivatives at the investigated concentration. FT-IR spectroscopy in D_2_O (Figure 1c) further confirmed the results obtained from the UV–vis experiments, showing a peak at 1664 cm^–1^ in the amide I region corresponding to the C=O stretch of the hydrogen-bonded 4­[1*H*] tautomer in the spectra of **C12**-O**** and **C10**-O****. In comparison, in the spectra of **C8**-O**** and **C6**-O****, the absorption at 1664 cm^–1^ decreases proportionally to the decrease in the alkyl chain size and shoulder peaks at 1656 and 1649 cm^–1^ are present, respectively. This indicates weakening of hydrogen bonding probably due to a higher concentration of the free 6­[1*H*] tautomer in solution.^54^ ^1^H NMR spectroscopy was then employed to quantify the visible fraction of each monomer in the liquid-like phase or in small and mobile aggregates.^61,62^ In the NMR spectra of the synthesized monomers at 500 μM (Figure 1d), only the signals coming from **C6**-O**** and **C8**-O**** are all visible at room temperature, while for **C10**-O**** and **C12**-O****, only the broad peak corresponding to the mOEG_11_ chain at 3.47 ppm is visible. Indeed, the visible percentage of the hard block of both **C10**-O**** and **C12**-O**** is 0 mol %, indicating equal low mobility due to full assembly. On the other hand, the molar fraction of the **C6**-O**** hard block in the liquid-like phase is 81 mol %, while it is 32 mol % for the **C8**-O****, indicating that **C8**-O**** presents in average a lower degree of mobility than **C6**-O****, which might be ascribed to the formation of more rigid assemblies (*vide infra*). Furthermore, the signals corresponding to the alkylidene proton and the methyl group of the UPy ring in **C8**-O**** are upfield shifted compared to those of **C6**-O**** (Figure S44), indicating enhanced assembly for the former resulting from close contact of the aromatic rings, as previously described.^37^ This is further corroborated by the higher absorbance at 280 nm in the UV–vis spectrum of **C6**-O**** (Figure S43a) at 500 μM compared to that of **C8**-O****, which indicates lower dimerization of the former monomer. Interestingly, the percentage of visible mOEG_11_ for **C12**-O**** and **C10**-O**** could not be calculated due to the broadness of the corresponding peaks, suggesting restricted mobility of the hydrophilic moiety likely caused by its involvement in the assembly formation (*vide infra*). In contrast, **C6**-O**** and **C8**-O**** exhibit 100 mol % of visible mOEG_11_, indicating a higher degree of mobility and negligible involvement in assembly formation compared to the former monomers. As a result, the assembly process of the synthesized molecules seems to be driven by the relative amount of the 4­[1*H*] tautomer, which, in turn, is influenced by the size of the alkyl spacer connected to the mOEG_11_ chain. Indeed, **C10**-O**** and **C12**-O**** are fully assembled within the investigated concentration range as they are mostly present as 4­[1*H*] tautomers, while for **C6**-O**** and **C8**-O****, only part of the monomers is involved in assembly formation as their tautomeric equilibria are more shifted toward the 6­[1*H*] tautomer due to the shorter alkyl chains, as demonstrated by the respective UV–vis spectra (Figure S43a).

CryoTEM imaging and SAXS were employed to elucidate the morphology of the assemblies formed by the different monomers in water above the cac of each monomer (Figure 2a–f). CryoTEM shows isolated single fibers for **C6**-O**** (Figure 2a) and **C8**-O**** (Figure 2b) with similar diameters ranging from 7.0 to 8.0 nm (Figure 2e). However, even though the average diameter of the assemblies formed by **C6**-O**** and **C8**-O**** is the same, the two additional methylene units in the alkyl chain of the latter appear to promote the elongation of the assemblies, leading to μm-long fibers unlike the ones formed by **C6**-O**** that are imaged as nanometers long at the investigated concentration. In comparison, bundles of two μm-long fibers are observed for **C10**-O**** (Figure 2c) and **C12**-O**** (Figure 2d), with similar diameters ranging from 10 to 12 nm (Figure 2e). Remarkably, negligible differences in the diameter of the assemblies formed by each monomer were observed at higher concentrations (Figure S63).

**Figure 2:**
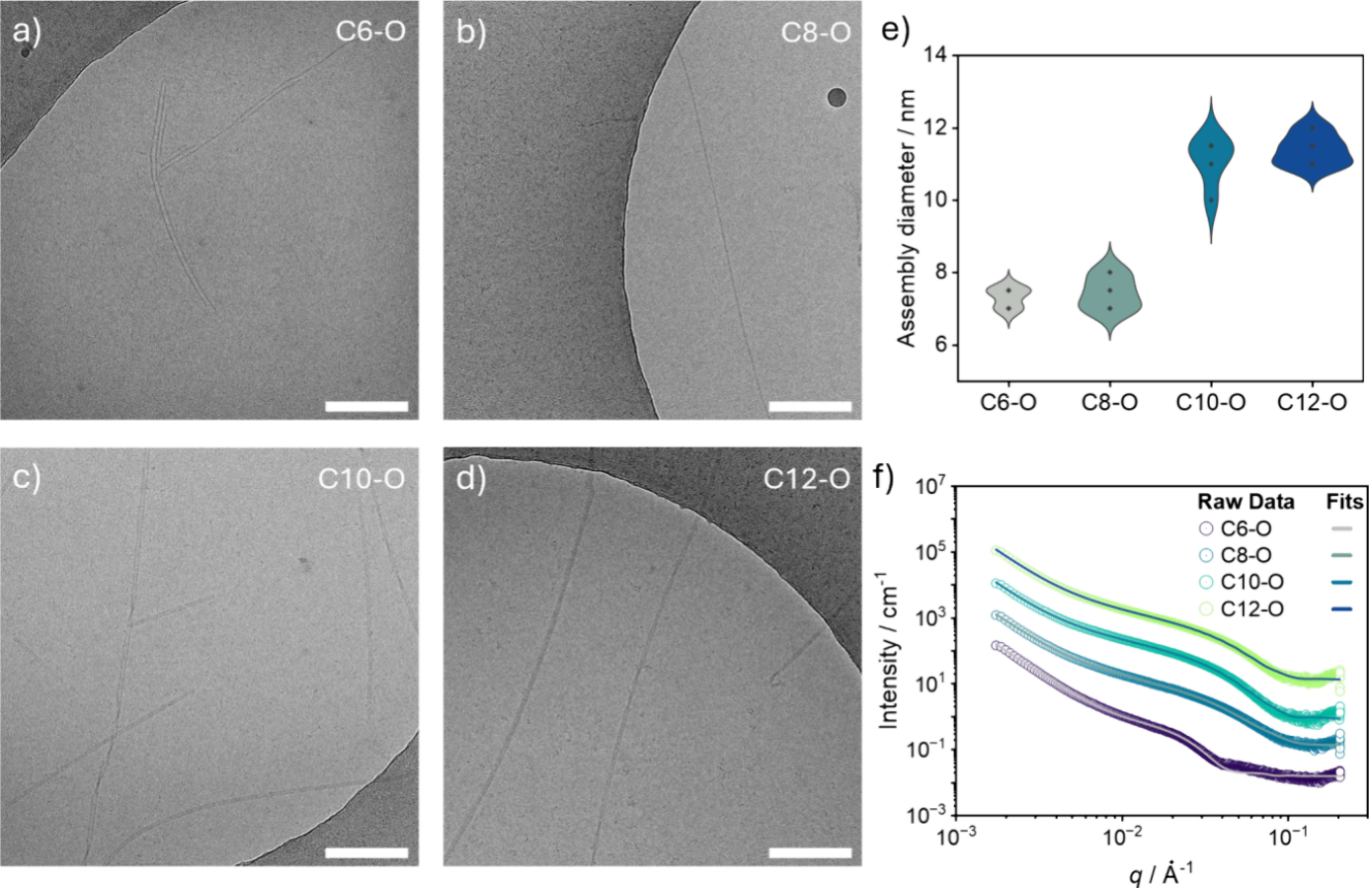
Morphological study of the assembled monomers in water. Representative cryoTEM images of (a) **C6**-O****, (b) **C8**-O****, (c) **C10**-O****, and (d) **C12**-O**** in MQ water (*C* = 100 μM) showing a clear transition from individual fibers (a, b) to bundles of fibers (c, d) by increasing the length of the alkyl spacer next to the urea group. Scale bars: 200 nm. (e) Violin graph displaying differences in the diameter of the assembled structures formed by each monomer. (f) Plot of the SAXS data (circles) and fits (solid lines) of the assembled monomers in water (*C* = 2.5 mM). Data are vertically shifted for the sake of clarity.

The scattering data (Figure 2f) of **C12**-O****, **C10**-O****, and **C8**-O**** could be fitted best to an elliptical cylinder model (Table S7), confirming the presence of elongated cylindrical structures in solution with a similar radius of 3.0 nm. On the other side, the scattering curve of **C6**-O**** could be fitted best to a cylinder model (Table S7), indicating the formation of cylinders with an average radius of 8.2 nm. These results suggest that the bundles observed in the micrographs of **C10**-O**** and **C12**-O**** might be composed by two fibers with a radius of 3.0 nm, while confirming the formation of single fibers for **C8**-O**** with the same radius of 3.0 nm. Interestingly, the assemblies formed by **C6**-O**** seem to be wider than those observed in the respective cryoTEM micrographs. This might be attributed to the lateral aggregation of the fibers into larger aggregates (Figure S62), which are forced apart during the cryoTEM sample preparation by shear forces. As a result, predominantly small and single fibers become visible during the cryoTEM imaging, while the original size of the fiber in solution is detected by the less invasive SAXS measurement. From the morphological analyses, 10 methylene units in the alkyl spacer next to the mOEG_11_ chain appear to be the hydrophobic threshold to induce bundle formation in solution, below which only single fibers are observed.

To compare the molecular dynamics of the structures formed by the monomers in solution, hydrogen–deuterium exchange followed by mass spectrometry (HDX-MS) experiments (Figure 3a) were carried out. This technique is widely employed to study the dynamicity of supramolecular polymers in solution by following the exchange of the labile hydrogens involved in hydrogen bonding with deuterium atoms.^21,55,63^ To this end, the monomers were assembled in MQ water at 2.5 mM and diluted 25-fold to a final concentration of 100 μM, at which fiber formation was observed for all the monomers. The exchange of the labile hydrogens with deuterium was then followed with MS over 7 days. The synthesized molecules bear five exchangeable protons all embedded in the hard block. Three minutes after dilution, the amount of fully exchanged monomers (**Cn-O-5D**) varies depending on the alkyl chain length, similarly to what was observed for BTA and ureido toluene bisurea molecules.^21,63^ Indeed, the percentage of **C12**-O**-5D** goes from 28% 3 min after dilution to 55% after 7 days, while a much faster molecular exchange is observed for **C10**-O****, where the **C10**-O**-5D** percentage after 3 min is 79% and levels off to 89% after 7 days. By reducing the methylene units further, **C6**-O**** and **C8**-O**** show the fastest dynamicity with 100% of **C6**-O**-5D** and **C8**-O**-5D** observed 3 min after dilution because of the shorter shielding alkyl chains. These results suggest that, at the examined concentration, the more hydrophobic the molecule is, the more stable are the assemblies derived from it. In particular, for **C12**-O**** and **C10**-O**** structures, complete H/D exchange is never observed in the experiment time frame, suggesting the presence of more static structures than those formed by **C6**-O**** and **C8-O**.

**Figure 3:**
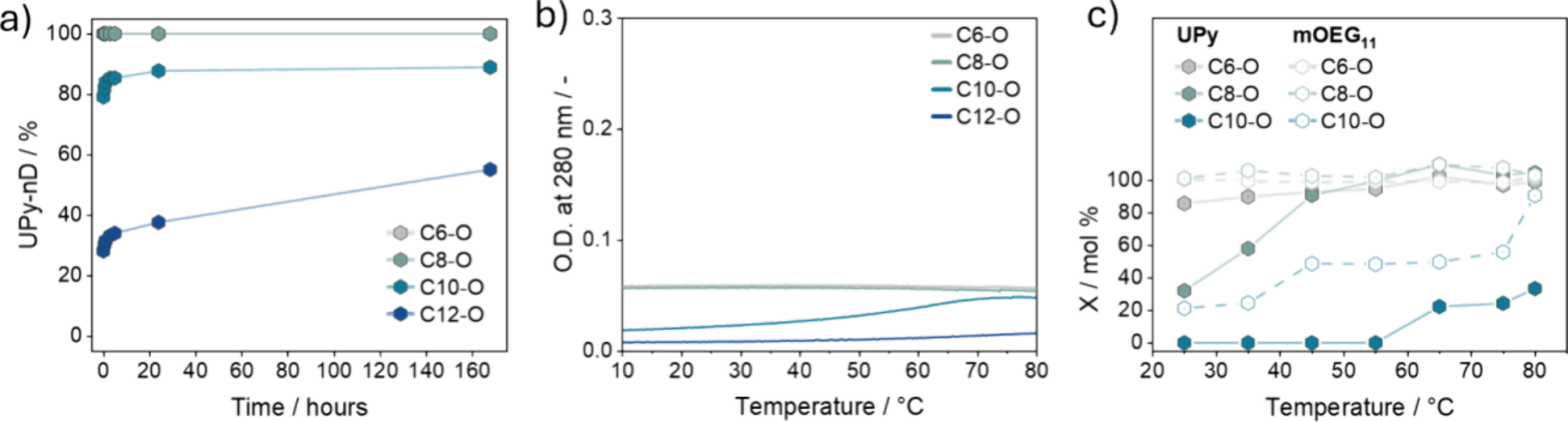
Assembly (a) dynamics and (b, c) kinetics study on the assembled monomers in water. (a) Percentage of fully deuterated UPy molecules as a function of time after 25× dilution of 2.5 mM aqueous samples in D_2_O. (b) Cooling curves of each monomer in water (*C* = 100 μM, 0.1 °C·min^–1^, and *l* = 1 mm) and (c) mole fraction (*X*) quantification of the UPy motif (solid lines and filled dots) and mOEG_11_ chain (dashed lines and empty dots) in the liquid-like phase for **C6**-O****, **C8**-O****, and **C10**-O**** at different temperatures (25–80 °C) in D_2_O (*C* = 500 μM).

The influence of the size of the alkyl spacer on the thermal stability of the assemblies formed by each monomer was analyzed through variable-temperature UV–vis (VT-UV) and ^1^H NMR (VT-NMR) spectroscopy (Figure 3b,c) above the cac, at 100 and 500 μM, respectively. Upon heating the samples up to 80 °C, different degrees of increase in absorbance at 280 nm, indicating the conversion to the 6­[1*H*] tautomer, are observed (Figure. S42). Indeed, the cooling curves obtained from the VT-UV (Figure 3b) indicate negligible conversion of **C12**-O**** into the 6­[1*H*] tautomer at high temperature, while the one of **C10**-O**** shows nearly quantitative conversion above 73 °C. On the other hand, the cooling curves of both **C6**-O**** and **C8**-O**** hardly show any absorbance variation at 280 nm over the temperature range, confirming the presence of the 6­[1*H*] tautomer, at the explored concentration. These results imply that the aggregates formed by **C12**-O**** are the more stable among the tested ones, while decreasing the number of methylene units in the alkyl spacer leads to less stable assemblies due to the conversion of the 4­[1*H*] tautomer into the 6­[1*H*] tautomer at elevated temperatures.

VT-NMR experiments in D_2_O (Figure 3c) corroborate the VT-UV results and provide further information about the parts of the molecule involved in the assembly process. Besides a broad peak ascribed to the mOEG_11_ chain, no peaks were found in the spectra of **C12**-O**** (Figure S48), indicating the low mobility of the monomers and high stability of the assemblies over the temperature range. However, owing to the lack of good signals, no monomer quantification was possible. For **C10**-O**** (Figure S47), the peaks corresponding to the UPy core become visible only at 65 °C and the respective visible percentage is calculated to be 22 mol %. Interestingly, the signals corresponding to the mOEG_11_ chain become sharper at 45 °C, underlining its higher degree of mobility compared to the hard block, probably due to its peripheral position toward the aqueous interface. At 80 °C, the percentage of the UPy core in the liquid-like phase increases to 34 mol %, whereas the one corresponding to the mOEG_11_ chain reaches 91 mol %. This indicates nearly full mobility of the hydrophilic region at high temperature, while a significant fraction of the hard block remains involved in assembly formation with low mobility. On the other hand, even if the percentage of UPy in the liquid-like phase for both **C6**-O**** and **C8**-O**** reaches 100 mol % above 55 °C, below that temperature, the percentage of visible UPy for **C8**-O**** is lower (as reported above), indicating lower mobility of the respective hard block compared to that of **C6**-O**** and higher stability of the formed assemblies. Unlike the more hydrophobic monomers, the fraction of mOEG_11_ in the liquid-like phase is 100 mol % over the whole temperature range for both **C6**-O**** and **C8**-O****. Thus, the assemblies formed by **C12**-O**** are more stable, while reducing the number of methylene units in the hydrophobic spacer leads to a gradual decline in the stability of the corresponding assemblies and increased mobility of the monomers within them, in agreement with the HDX-MS and VT-UV experiments.

### Influence of pH in the Assembly Process

UPy molecules are known to be pH-responsive due to the tautomeric equilibrium between the keto and enol group within the UPy ring, which can be deprotonated into enolate under basic conditions and hamper the UPy dimerization through electrostatic repulsion (Figure 4a).^42^ The effect of the pH on the assembly of each monomer was first investigated by UV–vis spectroscopy. The UV–vis spectra of all the molecules (Figure S43b–e) at pH 12 show an absorption maximum at 272 nm, which corresponds to the enolate form.^38,64^ The UV–vis spectra of **C10**-O**** (Figure S43d) and **C12**-O**** (Figure S43e) at pH 3.0 and pH 7.0 present the classic features of the 4­[1*H*] tautomer, indicating the assembly of the respective monomer at both pHs. On the other hand, the UV–vis spectra of **C6**-O**** (Figure S43b) and **C8**-O**** (Figure S43c) present a hypsochromic shift from 284 to 280 nm and from 290 to 285 nm, respectively, from acidic to neutral pH, suggesting the formation of different interactions between the monomers at the different pHs.^65^

**Figure 4:**
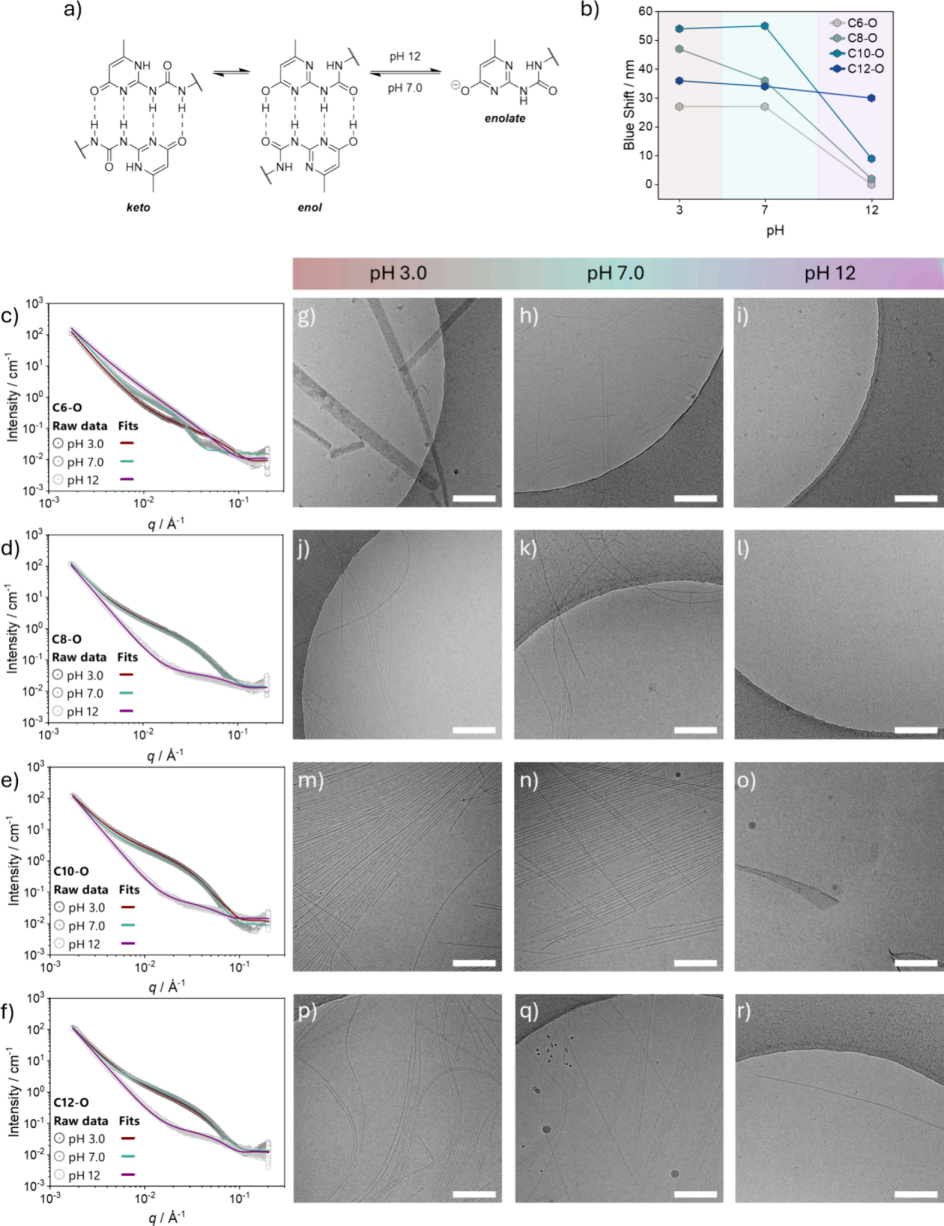
pH responsiveness of the supramolecular polymers formed by each monomer in water. (a) Enolate formation within the UPy ring at a basic pH. (b) pH-dependent NRF assay of each monomer at pH 3.0, pH 7.0, and pH 12. Plot of the SAXS data (circles) and fits (solid lines) of (c) **C6**-O****, (d) **C8**-O****, (e) **C10**-O****, and (f) **C12**-O**** in MQ water at pH 3.0, 7.0, and 12 (*C* = 2.5 mM). Representative cryoTEM images of (g–i) **C6**-O****, (j–l) **C8**-O****, (m–o) **C10**-O****, and (p–r) **C12**-O**** in MQ water at pH 3.0, pH 7.0, and pH 12 (*C* = 500 μM). Scale bars = 200 nm.

^1^H NMR spectroscopy in D_2_O at pD 3.0, 7.0, and 12 was then used to investigate the effects of pD on the monomer mobility within the assemblies and the transition from the keto to enolate form. At pD 3.0 and 7.0, the spectra of **C10**-O**** (Figure S51) and **C12**-O**** (Figure S52) showed restricted mobility due to assembly formation, while at pD 12, the increased polarity of both monomers in the enolate form enhanced their mobility. The percentage of visible UPy in the liquid-like phase is 22.5 mol % for **C12**-O**** and 24.7 mol % for **C10**-O**** indicating a similar increased mobility for both monomers under basic conditions. Similarly, the percentage of visible mOEG_11_ ranges from 26 mol % for **C12**-O**** to 29 mol % for **C10**-O****, indicating similar mobility for both the hard block and the mOEG_11_ chain at basic pD. In comparison, the percentage of visible UPy increases from 82 to 100 mol % for **C6**-O**** (Figure S49) and from 32 to 100 mol % for **C8**-O**** (Figure S50) as the pD shifts from neutral to basic, indicating full mobility of both monomers under basic conditions upon enolate formation. Indeed, distinct shifts in the alkylidene proton signals confirm the conversion from keto to enolate within the UPy ring.

The influence of the pH on the assembly formation was further investigated with the NRF assay (Figure 4b). No significant differences in the blueshift of **C12**-O**** are observed at the different pHs, suggesting the formation of assemblies of similar polarity under all the tested conditions. Two fewer methylene units in **C10**-O**** result in the formation of assemblies with analogue polarity at pH 3.0 and pH 7.0, while a drop in the blueshift is observed at basic pH, suggesting disassembly of the aggregates. In contrast, **C8**-O**** exhibits a progressive decrease in the blueshift from acidic to basic pH, indicating the formation of aggregates of different polarity at acidic and neutral pH, while disassembly under basic conditions. Similarly to **C10**-O**** but with lower blueshift values, **C6**-O**** displays a steady blueshift from acidic to neutral pH and a drop at basic pH, indicating the presence of assemblies of similar polarity from acidic to neutral pH and disassembly at basic pH.

Finally, the influence of the pH on the morphology of the assemblies was studied via SAXS (Figure 4c–f) (Tables S6–S11) and cryoTEM (Figure 4g–r). The scattering curve of **C6**-O**** (Figure 4c) at acidic pH could be fitted best to a flexible cylinder model, suggesting the presence of fibers with an average radius of 2.4 nm, while the curve at neutral pH could be fitted best to a cylinder model, indicating the presence of fibers with an average radius of 8.2 nm. The data at basic pH could be fitted best to a lamella model, suggesting the presence of 5.0 nm-thick lamellae or disks in solution. CryoTEM images of **C6**-O**** confirmed the pH-dependent morphology observed with SAXS. At acidic pH (Figure 4g), large polydisperse ribbon-like aggregates are present surrounded by smaller single fibers, while at neutral pH (Figure 4h), single fibers are visualized with the radius matching with the SAXS analysis. Finally, at basic pH (Figure 4i), small micelles are observed, in agreement with SAXS analysis. On the other hand, the scattering curves of **C8**-O**** (Figure 4d), **C10**-O**** (Figure 4e), and **C12**-O**** (Figure 4f) could be best fitted to an elliptical cylinder model both at pH 3.0 and pH 7.0, indicating the formation of cylindrical structures with radii of 2.8 and 2.4 nm at acidic and neutral pH, respectively, for **C8**-O**** and an unchanged radius of 3.0 nm for **C10**-O**** and **C12**-O**** at both pHs. At basic pH, the scattering curves of all the aforementioned monomers could be fitted best to a sphere model, suggesting the formation of small micelles with a radius ranging from 3.0 to 4.0 nm. Indeed, the micrographs of **C8**-O**** show a mixture of μm-long bundled and single fibers at acid pH (Figure 4j), while mostly single fibers at neutral pH (Figure 4k). In comparison, small micellar structures were observed under basic conditions (Figure 4l), in agreement with the NRF assay results and SAXS data. Two more methylene units in **C10**-O**** lead to μm-long bundled fibers at pH 3.0 (Figure 4m) and 7.0 (Figure 4n). Remarkably, poorly organized structures were visualized at basic pH (Figure 4o), in agreement with the drop in blueshift obtained from the NRF assay. Lastly, the cryoTEM micrographs of **C12**-O**** (Figure 4p–r) show a homogeneous distribution of μm-long bundled fibers of similar diameters both at acidic (Figure 4p) and neutral pH (Figure 4q), while mostly small aggregates along with fewer spare single fibers under basic conditions (Figure 4r), in agreement with the drop in scattering intensity of the respective SAXS curve.

These results suggest that the size of the alkyl spacer next to the mOEG_11_ chain does not prevent enolate formation under basic conditions, but it affects the pH responsiveness of the resulting assemblies. Indeed, 10 methylene units appear to be the threshold below which clear morphological transitions are observed by changing the pH of the medium.

### Mechanical and Dynamic Characterization of Supramolecular Hydrogels

In order to study the mechanical and dynamic properties of the synthesized molecules in bulk, they were formulated in supramolecular hydrogels by increasing the concentration (9.8 mM, ∼1.0% w/v) to where previous UPy based molecules are able to entangle and form a hydrogel.^48,66^ The different compounds were therefore dissolved at basic pH and high temperatures using a previously established protocol.^67^ After neutralization, the molecules were allowed to assemble at 37 °C for 24 h. Afterward, rheological measurements (Figure S56) showed the formation of soft networks for **C12**-O**** and **C6**-O**** (*G*′ ∼10 Pa) with a storage modulus close to the error limit of the instrument. While this was expected for the **C12**-O****, as cryoTEM revealed the formation of long fibers capable of entangling into a hydrogel, the ability of **C6**-O**** to form a soft hydrogel remains unexpected, especially in contrast to **C8**-O**** and **C10**-O****, which formed liquid dispersions (*G*′ ∼1 Pa, raw phase ∼180 °), highlighting significant differences in the capability of forming a network for the different monomers. For **C6**-O****, this might point toward the formation of a different macroscopic network compared to those formed by the other monomers, as previously suggested by the SAXS data. Nevertheless, it is important to note that the measured *G*′ is exceptionally low and close to the machine error limit. In contrast, **C8**-O**** and **C10**-O**** formed liquid dispersions (*G*′ ∼1 Pa, raw phase ∼180 °), showing a large contrast between the ability of the molecules to form a network. Nevertheless, the networks formed by the synthesized molecules yielded only very soft hydrogels, which are likely not stiff enough to support cell adhesion and spreading.

In the past, stronger elastic-like hydrogels could be formed by incorporation of a small amount of a telechelic UPy-PEG_10k_-UPy molecule (**BF**) (Figure 5a), which is able to form cross-links between the UPy fibers.^29,48^ In this respect, a small amount of **BF** (0.1 mM) was added to the UPy molecules, in a **UPy**:**BF** molar ratio of 80:1 (Table S1). Similar to before, the molecules were allowed to assemble at 37 °C for 24 h. All mixtures were now able to form much stiffer elastic-like networks (*G*′ > 300 Pa, tan δ: 0.1–0.2; with tan δ = *G*″/*G*′) (Figure 5b and Figure S57). Combinations of **BF** with **C12**-O**** or **C10**-O**** resulted in hydrogels with similar mechanics, i.e., *G*′ ∼700 Pa and tan δ ∼0.15. Remarkably, **C8**-O**** with **BF** formed the stiffest hydrogel network (*G*′ ∼1000 Pa), while **C6**-O**** formed the softest hydrogel (*G*′ ∼350 Pa). We hypothesize that the strong network of **C8**-O**** with **BF** is attributed to the formation of single fibers instead of bundles (i.e., entanglement of multiple fibers), as observed in the cryoTEM images (Figure S64). This allows for a more homogeneous distribution of the cross-links between the individual fibers, which can cause a large increase in the mechanical stiffness. In contrast, the soft network of **C6**-O**** with **BF** is likely caused by the inability of the molecules to form long robust fibers, causing inferior mechanics.

**Figure 5:**
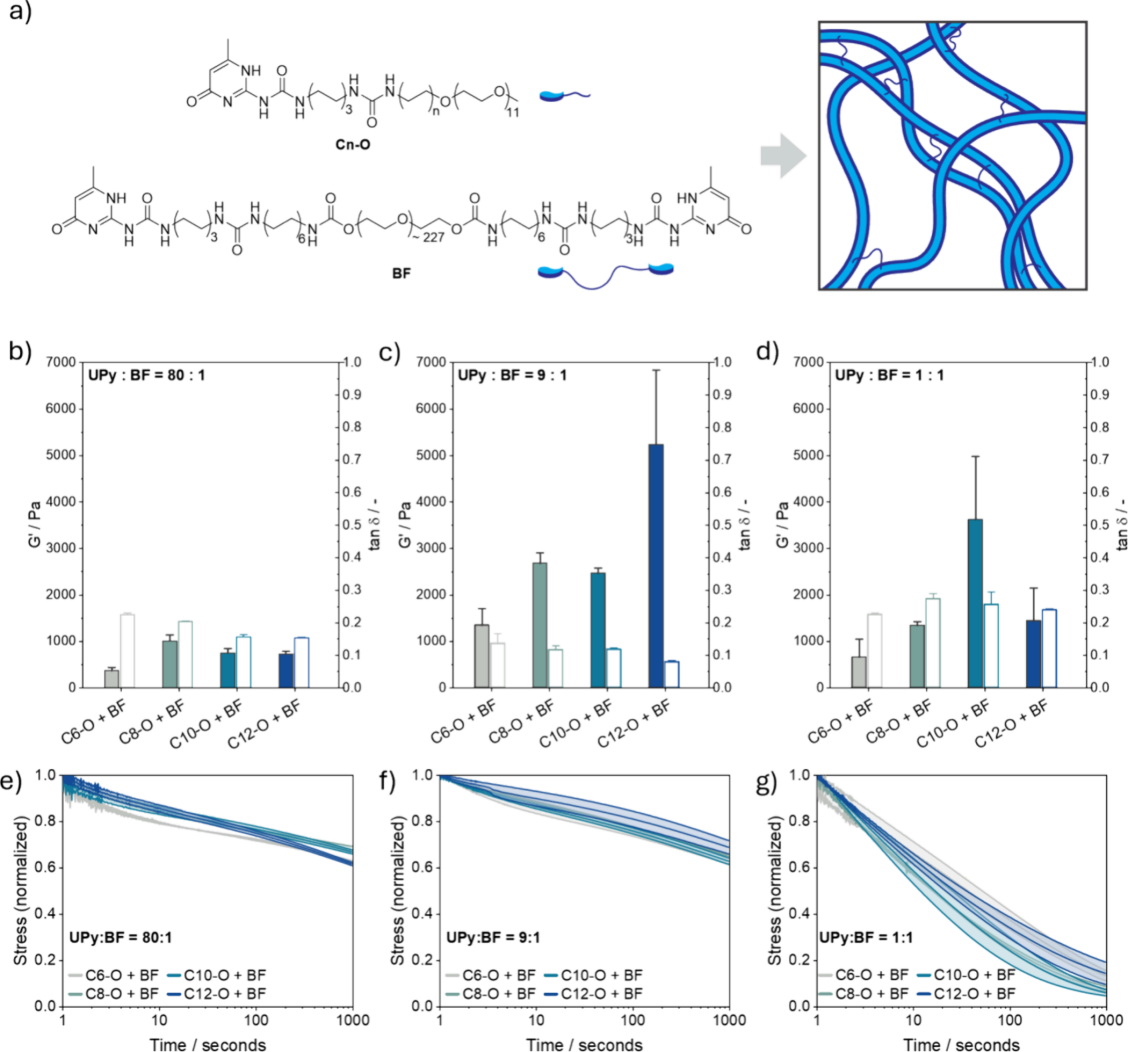
Mechanical properties of hydrogel networks composed of the various (a) UPy molecules mixed with a **BF** cross-linker. (b–d) Storage modulus (solid bars) and loss tangent (empty bars) of the various networks (*y* = 0.01 and ω= 1 rad s^–1^) at (b) **UPy**:**BF** = 80:1, (c) at 9:1, and (d) 1:1 showing that the ratio has a large effect on the storage modulus, while small effects can be observed by using a different UPy structure. The viscoelastic ratio stays similar for all gels in 80:1 and 9:1 ratios, while increasing the ratio to 1:1 leads to more viscous networks. (e–g) Stress relaxation of the various networks at different **UPy**:**BF** ratios, (e) 80:1, (f) 9:1, and (g) 1:1 following the released stress over 1000 s. All networks show similar stress decays over time depending on the **UPy**:**BF** ratio. The bar graph represents means with SEM.

In addition, bulk dynamics were investigated using frequency-dependent measurements (Figure S57), showing similar time-dependent viscoelastic behavior between all networks, i.e., *G*′ ≫ *G*″ over all frequencies. Furthermore, stress relaxation experiments were conducted, which followed the decrease in stress after strain was applied (Figure 5e). Here, all networks showed a similar stress decay over time, with ∼35% released stress after 1000 s (Figure S58), highlighting the rigidity of the networks. In addition, a continuous relaxation spectrum was fitted to the different relaxation curves (Figure S58) to obtain insight into the underlying relaxation mechanisms. Again, similar results were obtained for the different networks, with one significant relaxation mechanism at short timescales (∼0.1–1.0 s) and a second much longer relaxation mechanism (>10,000 s), which is not experimentally probed. Finally, also strain-dependent measurements showed similar behavior between all the different networks, i.e., *G*′–*G*″ crossover ∼35% (Figure S59). These results highlight the similarities in bulk dynamics between the different hydrogels.

The molar ratio of 80:1 between monofunctional monomers and **BF** was chosen as this previously resulted in solid-like hydrogels (i.e., *G*′ > *G*″) at low building block concentrations and favored cell adhesion and spreading.^48^ Nevertheless, in the past, hydrogel mechanics and dynamics could be tuned simply by the molar ratio between the two building blocks, i.e., monofunctional **UPy** and **BF**.^29,48^ To explore similar tunability in the above-described hydrogels, the ratio between **UPy:BF** was changed to 9:1 and ultimately to 1:1, while keeping the total amount of UPy molecules fixed (i.e., 9.87 mM) (Tables S2 and S3). Changing the ratio to 9:1 resulted in the formation of stiffer hydrogels compared to 80:1 (*G*′ > 1000 Pa) (Figure 5c). We attribute this increase to the rise of the % w/v of the gels (as **BF** has a much larger molecular weight) and to the ability of **BF** to percolate and form an elastic-like network by itself.^48,66,68–70^ Similar to the case before, **C6**-O**** with **BF** formed the softest network (*G*′ ∼1500 Pa). Combinations of **BF** with **C8**-O**** or **C10**-O**** resulted in hydrogels with similar mechanics, i.e., *G*′ between 2000 and 3000 Pa. This is in contrast to **C12**-O**** with **BF**, which formed the stiffest hydrogel *G*′ ∼5000 Pa. However, it is important to note the large deviation for these gels, especially compared to the small deviations observed for the 80:1 hydrogels. We attribute this difference to the fast gelation of **BF** by itself, which can greatly impact the inhomogeneity in the sample and thereby the bulk mechanics. In contrast to these changes in *G*′, the relative elasticity (tan δ) remained largely unchanged, i.e., tan δ ∼0.1 (Figure 5c). Furthermore, the dynamics between the different hydrogels were similar and showed no significant differences between the gels that contained an 80:1 ratio (Figure 5e,f). All gels released ∼35% stress after 1000 s (Figure S60). In addition, stress relaxation spectra remained similar to the most important relaxation mechanism at short timescales (<1 s) (Figure S60). Nevertheless, the spectra revealed additional relaxation mechanisms on the intermediate and long timescales (1–10,000 s) that were not present in the 80:1 hydrogels. This might indicate the formation of a more complex interactive network between the monofunctional UPy monomers and the **BF**, or additional relaxation mechanisms that are introduced between the **BF** molecules themselves, as these molecules are also able to percolate and form a separate network at these concentrations. These additional mechanisms are most obvious for the networks that can form longer fibers, especially for **C12**-O****, which showed a broad range of different relaxation mechanisms (Figure S60). This large range of mechanisms might indicate the formation of additional interactions between the different molecules, which could explain the higher *G*′ that was obtained for this network.

Finally, mixtures were made in a 1:1 molar ratio of **UPy**:**BF** (Table S3). The formed hydrogels showed a drop in *G*′ compared to the 9:1 ratio, reaching a *G*′ of ∼1000 Pa, except for **C10**-O**** with **BF**, which reached a stiffness of ∼3500 Pa (Figure 5d). This drop in *G*′ compared to the 9:1 ratio might indicate that the molecules and assemblies start to interfere with one another, i.e., the % w/v of **BF** becomes sufficiently high to allow the formation of its own elastic network, which could hamper the cross-linking with the monofunctional molecules and cause phase separation.^66^ Especially, **C12**-O****, which is able to form less dynamic fibers, seems to be hindered by **BF**, as the deviation between the samples becomes larger, indicating inhomogeneities in the network. On the other hand, **C6**-O**** with **BF** still formed the softest gel, indicating that these fibers might still be too short to form a stiff network. The network of **C10**-O**** with **BF** becomes remarkably stiffer when it transitions from a 9:1 to 1:1 ratio. This change is likely due to the formation of a more complex network at the latter ratio, where **BF** not only forms a network by itself but also acts as a cross-linker between the fibers of **C10**-O****. In this case, the **C10**-O**** fibers may provide an optimal framework for accommodating and supporting the development of such an intricate network, ultimately leading to an increased stiffness. In contrast to the earlier ratios (80:1 and 9:1), the networks with a 1:1 ratio formed more viscous networks, as indicated by a higher tan δ of ∼0.2 (Figure 5d). In addition, more dynamic networks were obtained, as observed by stress relaxation experiments (Figure 5g). All the different networks were able to relax ∼80% stress after 1000 s (Figure S61). Remarkably, there was no large difference between the different molecules, i.e., all networks showed a similar fast stress relaxation. This fast stress relaxation coincided with changes in the relaxation spectra, as more important relaxation mechanisms occurred at short and intermediate timescales (<100 s) and there was no indication of a long relaxation mechanism (>10,000 s) (Figure S61). Comparing the stress relaxation of the different ratios (Figure 5e–g) revealed a similar pattern, i.e., a similar stress relaxation curve for all networks as long as the ratio between the building blocks stays similar. Only at a 1:1 ratio, a faster stress relaxation was observed for all the molecules, indicating that the ratio between the mono and **BF** molecules is responsible for the bulk dynamics of the networks rather than the intrinsic molecular design of the molecule itself. A similar conclusion can be observed from the bulk mechanics, i.e., *G*′, which is largely influenced by the ratio between the building blocks. Nevertheless, some substantial differences in *G*′ could be observed between the different molecules. Indeed, **C6**-O**** with **BF** always formed the softest hydrogel network likely due to the formation of smaller fibers. In comparison, all the other networks showed a similar stiffness, although the stiffest network was always formed by a different molecule. The formation of the stiffest networks in every ratio is likely caused by a complex interplay between the length of the fibers, the ability to incorporate homogeneous cross-links and the inhomogeneities and interference when a large amount of **BF** is incorporated.

### Influence of the Alkyl Spacer on Cellular Spreading

The influence of the alkyl spacer length of each monomer on the cell spreading behavior of human normal dermal fibroblasts (hNDFs) was investigated. To this end, hydrogels at **UPy**:**BF** = 80:1 were formulated with 1 mM UPy-cRGD to promote cell adhesion (Table S4).^29^ Confocal microscopy images demonstrated appropriate cell adhesion and spreading on all hydrogels after 1 day (Figure 6a–d) in the 2D culture. However, cells on the **C6**-O**** (Figure 6a) hydrogel exhibited less spread and had shorter protrusions. Indeed, the percentage of the surface area covered by cells (Figure 6e) shows that the shortest **C6**-O**** exhibits the least cell spreading, whereas the **C8**-O****, **C10**-O****, and **C12**-O**** result in a modest increase in the cell-occupied area, probably due to a more homogeneous distribution of the networks (Figure S64) and greater stiffnesses (Figure 5b) compared to that of **C6**-O****. To further investigate the cell–material interactions, the intracellular distribution of the mechanotransduction marker yes-associated protein (YAP) was quantified.^71^ YAP is a transcriptional cofactor within the Hippo signaling pathway, whose subcellular localization is regulated by mechanical properties such as hydrogel stiffness and viscoelasticity. YAP is known to translocate to the nucleus on stiffer substrates but remains predominantly in the cytoplasm on softer substrates.^72,73^ Using a custom cell profiler pipeline, the intensity of YAP expression in both the nuclei and the cytoplasm was measured. Across all conditions, the nuclear-to-cytoplasmic YAP ratio exceeded 1, suggesting that the mechanotransduction was active in all hydrogels (Figure 6f). However, no significant differences in YAP localization ratios were observed between the tested conditions. Cells were then encapsulated in the 3D hydrogels (Table S5) for 3 days. In the **C6**-O**** (Figure S65a) and **C12**-O**** (Figure S65d) hydrogels, cells largely remained round and nonspreading, while some degree of spreading was observed in **C8**-O**** (Figure S65b) and **C10**-O**** (Figure S65c) hydrogels, though still reduced compared to 2D cultures (Figure 6e). This reduced spreading in 3D might be attributed to the greater physical constraints imposed by the hydrogels.^74–76^ Indeed, even though 3D hydrogels more closely mimic *in vivo* conditions by providing mechanical cues in all three dimensions, they may also sterically hinder cell spreading. In contrast, 2D cultures, lacking such constraints, allow cells to spread unconstrained within the *x–y* plane. Another hypothesis might be related to the differences in molecular dynamics observed in diluted conditions, which can result in a more adaptable distribution of the cell-adhesive ligand within the networks formed by **C8**-O**** with **BF** and **C10**-O**** with **BF** rather than within the hydrogel formed by **C12**-O**** with **BF**, thus promoting cell spreading in 3D culture.

**Figure 6:**
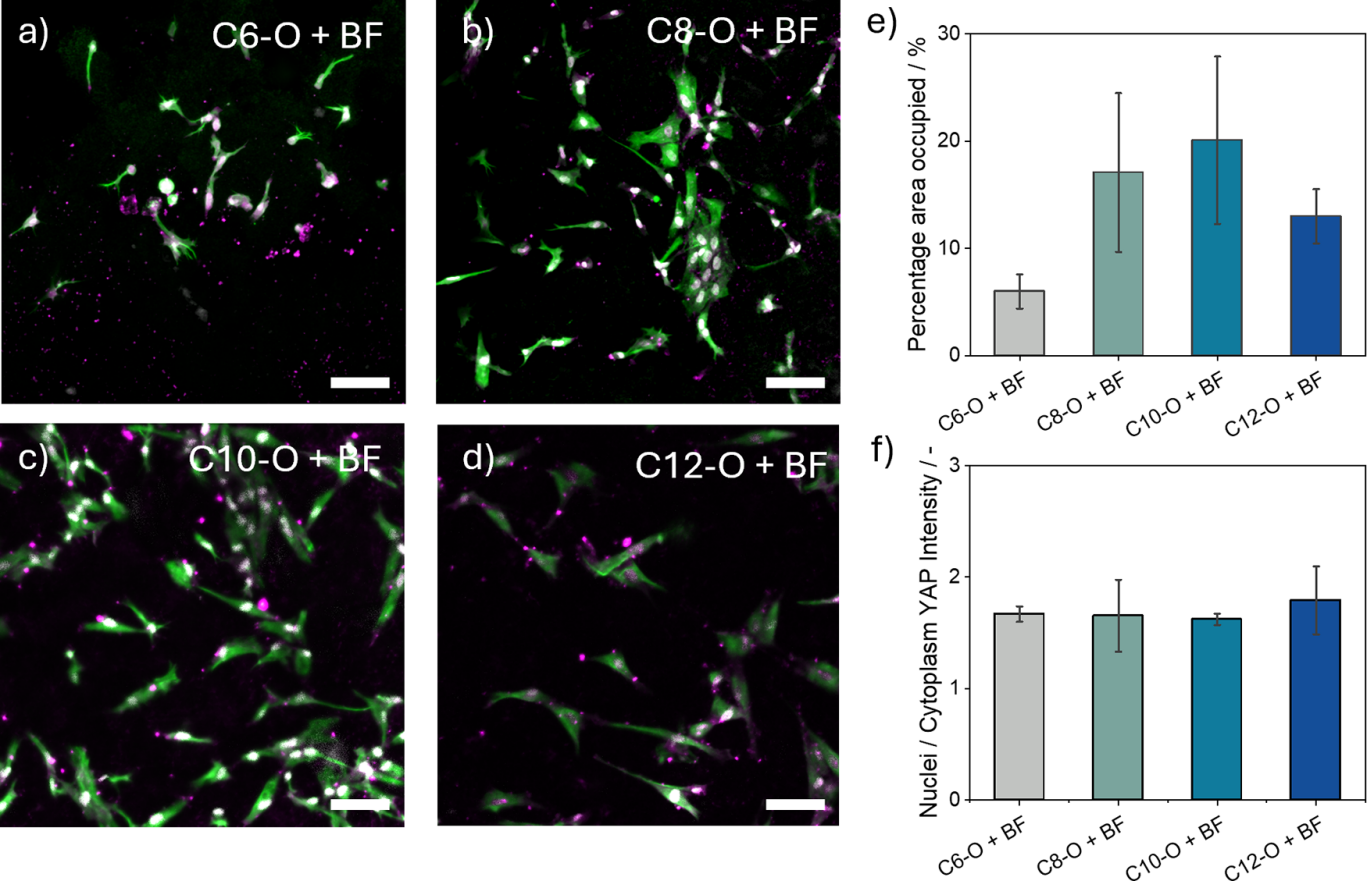
(a–d) Cell culture in 2D on hydrogels formulated with the different monomers after 1 day of incubation (cytoskeleton, green; nuclei, white; YAP, magenta). Scale bars = 100 μM. (e) Percentage area covered (%) by cells and (f) quantified YAP nuclei-to-cytoplasm ratio. The bar graph represents means with SEM.

From these results, it can be concluded that only the hydrogels formed by the **C8**-O****, **C10**-O****, and **C12O** in combination with the **BF** can support cellular adhesion and spreading due to their viscoelastic properties in 2D culture, while this effect is not directly translated to the 3D culture.

## Conclusions and Outlook

To conclude, four new UPy molecules have been synthesized through a new facile synthetic approach, providing the pure final molecules in good overall yields (50–75%). The assembly studies in solution showed that all the monomers can assemble into elongated structures in water; however, 8 methylene units in the alkyl spacer are needed to yield μm-long elliptical cylinders. Interestingly, despite the difference in length, the aggregates formed by both **C6**-O**** and **C8**-O**** were imaged as single fibers of similar dynamicity in diluted aqueous solutions while more stable μm-long bundled fibers were observed for **C10**-O**** and **C12**-O**** in which also the mOEG_11_ chains present restricted mobility probably due to their involvement in the bundling process. Lastly, the thermal and pH stabilities of the resulting aggregates in solution were shown to increase by increasing the number of methylene units in the alkyl spacer next to the mOEG_11_ chain. Interestingly, in the bulk studies, all the monomers were able to form hydrogels of similar stiffness when combined with different ratios of **BF** except for **C6**-O****, probably due to the shorter length of the respective fibers. Furthermore, all the hydrogels formed by each monomer, except for **C6**-O****, in combination with the **BF** cross-linker in **UPy**:**BF** = 80:1 were able to sustain cell spreading in 2D culture in a similar fashion, indicating the need for a stiffness threshold, which is ultimately dictated by the monomer molecular design.

## Supplementary Material



## Notes

Synthetic procedures, molecular characterization data, additional UV–vis spectra, VT-^1^H NMR characterization and pD-dependent ^1^H NMR spectra, additional SAXS spectra and fitting parameters, full pH-dependent Nile red emission spectra, additional HDX-MS analysis, rheology analyses, and cryoTEM images of the hydrogels in 3D cell culture experiments (PDF)

## References

[1] Mouw J. K., Ou G., Weaver V. M. (2014). Extracellular Matrix Assembly: A Multiscale Deconstruction. Nat. Rev. Mol. Cell Biol..

[2] Vale R. D. (2003). The Molecular
Motor Toolbox for Intracellular Transport. Cell.

[3] van
den Ent F., Amos L. A., Löwe J. (2001). Prokaryotic
Origin of the Actin Cytoskeleton. Nature.

[4] Webber M. J., Appel E. A., Meijer E. W., Langer R. (2016). Supramolecular Biomaterials. Nat. Mater..

[5] Versluis F., van Esch J. H., Eelkema R. (2016). Synthetic
Self-assembled Materials
in Biological Environments. Adv. Mater..

[6] Flory P. J. (1974). Introductory
Lecture. Faraday Discuss. Chem. Soc..

[7] Li J., Mooney D. J. (2016). Designing Hydrogels
for Controlled Drug Delivery. Nat. Rev. Mater..

[8] Slaughter B. V., Khurshid S. S., Fisher O. Z., Khademhosseini A., Peppas N. A. (2009). Hydrogels in Regenerative Medicine. Adv. Mater..

[9] Mollet B. B., Comellas-Aragonès M., Spiering A. J. H., Söntjens S. H. M., Meijer E. W., Dankers P. Y. W. (2014). A Modular Approach to Easily Processable
Supramolecular Bilayered Scaffolds with Tailorable Properties. J. Mater. Chem. B.

[10] Hendricks M. P., Sato K., Palmer L. C., Stupp S. I. (2017). Supramolecular Assembly
of Peptide Amphiphiles. Acc. Chem. Res..

[11] Velichko Y. S., Stupp S. I., De La
Cruz M. O. (2008). Molecular Simulation Study of Peptide
Amphiphile Self-Assembly. J. Phys. Chem. B.

[12] Paramonov S. E., Jun H.-W., Hartgerink J. D. (2006). Self-Assembly
of Peptide–Amphiphile
Nanofibers: The Roles of Hydrogen Bonding and Amphiphilic Packing. J. Am. Chem. Soc..

[13] Lee S. S., Fyrner T., Chen F., Álvarez Z., Sleep E., Chun D. S., Weiner J. A., Cook R. W., Freshman R. D., Schallmo M. S., Katchko K. M., Schneider A. D., Smith J. T., Yun C., Singh G., Hashmi S. Z., McClendon M. T., Yu Z., Stock S. R., Hsu W. K., Hsu E. L., Stupp S. I. (2017). Sulfated Glycopeptide Nanostructures
for Multipotent Protein Activation. Nat. Nanotechnol..

[14] Mata A., Geng Y., Henrikson K. J., Aparicio C., Stock S. R., Satcher R. L., Stupp S. I. (2010). Bone Regeneration
Mediated by Biomimetic
Mineralization of a Nanofiber Matrix. Biomaterials.

[15] Álvarez Z., Kolberg-Edelbrock A. N., Sasselli I. R., Ortega J. A., Qiu R., Syrgiannis Z., Mirau P. A., Chen F., Chin S. M., Weigand S., Kiskinis E., Stupp S. I. (2021). Bioactive Scaffolds
with Enhanced Supramolecular Motion Promote Recovery from Spinal Cord
Injury. Science.

[16] Yuan S. C., Álvarez Z., Lee S. R., Pavlović R. Z., Yuan C., Singer E., Weigand S. J., Palmer L. C., Stupp S. I. (2024). Supramolecular Motion Enables Chondrogenic Bioactivity
of a Cyclic Peptide Mimetic of Transforming Growth Factor-Β1. J. Am. Chem. Soc..

[17] Álvarez Z., Ortega J. A., Sato K., Sasselli I. R., Kolberg-Edelbrock A. N., Qiu R., Marshall K. A., Nguyen T. P., Smith C. S., Quinlan K. A., Papakis V., Syrgiannis Z., Sather N. A., Musumeci C., Engel E., Stupp S. I., Kiskinis E. (2023). Artificial Extracellular
Matrix Scaffolds of Mobile Molecules Enhance Maturation of Human Stem
Cell-Derived Neurons. Cell Stem Cell.

[18] Lee S. S., Hsu E. L., Mendoza M., Ghodasra J., Nickoli M. S., Ashtekar A., Polavarapu M., Babu J., Riaz R. M., Nicolas J. D., Nelson D., Hashmi S. Z., Kaltz S. R., Earhart J. S., Merk B. R., McKee J. S., Bairstow S. F., Shah R. N., Hsu W. K., Stupp S. I. (2015). Gel Scaffolds of
BMP-2-binding Peptide Amphiphile Nanofibers for Spinal Arthrodesis. Adv. Healthcare Mater..

[19] Wright P. E., Dyson H. J. (2015). Intrinsically Disordered
Proteins in Cellular Signalling
and Regulation. Nat. Rev. Mol. Cell Biol..

[20] Lafleur R. P. M., Lou X., Pavan G. M., Palmans A. R. A., Meijer E. W. (2018). Consequences
of a Cosolvent on the Structure and Molecular Dynamics of Supramolecular
Polymers in Water. Chem. Sci..

[21] Lou X., Lafleur R. P. M., Leenders C. M. A., Schoenmakers S. M. C., Matsumoto N. M., Baker M. B., van Dongen J. L. J., Palmans A. R. A., Meijer E. W. (2017). Dynamic
Diversity
of Synthetic Supramolecular Polymers in Water as Revealed by Hydrogen/Deuterium
Exchange. Nat. Commun..

[22] Albertazzi L., van der Zwaag D., Leenders C. M. A., Fitzner R., van der
Hofstad R. W., Meijer E. W. (2014). Probing Exchange Pathways in One-Dimensional
Aggregates with Super-Resolution Microscopy. Science.

[23] Leenders C. M. A., Albertazzi L., Mes T., Koenigs M. M. E., Palmans A. R. A., Meijer E. W. (2013). Supramolecular Polymerization in Water Harnessing Both
Hydrophobic Effects and Hydrogen Bond Formation. Chem. Commun..

[24] Bakker M. H., Lee C. C., Meijer E. W., Dankers P. Y. W., Albertazzi L. (2016). Multicomponent
Supramolecular Polymers as a Modular Platform for Intracellular Delivery. ACS Nano.

[25] Wijnands S. P. W., Engelen W., Lafleur R. P. M., Meijer E. W., Merkx M. (2018). Controlling
Protein Activity by Dynamic Recruitment on a Supramolecular Polymer
Platform. Nat. Commun..

[26] Morgese G., de Waal B. F. M., Varela-Aramburu S., Palmans A. R. A., Albertazzi L., Meijer E. W. (2020). Anchoring Supramolecular
Polymers to Human Red Blood
Cells by Combining Dynamic Covalent and Non-Covalent Chemistries. Angew. Chemie Int. Ed..

[27] Leenders C. M. A., Mes T., Baker M. B., Koenigs M. M. E., Besenius P., Palmans A. R. A., Meijer E. W. (2014). From Supramolecular Polymers to Hydrogel
Materials. Mater. Horiz..

[28] Hafeez S., Aldana A. A., Duimel H., Ruiter F. A. A., Decarli M. C., Lapointe V., van Blitterswijk C., Moroni L., Baker M. B. (2023). Molecular
Tuning of a Benzene-1, 3, 5-Tricarboxamide Supramolecular Fibrous
Hydrogel Enables Control over Viscoelasticity and Creates Tunable
ECM-Mimetic Hydrogels and Bioinks. Adv. Mater..

[29] Rijns L., Peeters J. W., Hendrikse S. I. S., Vleugels M. E. J., Lou X., Janssen H. M., Meijer E. W., Dankers P. Y. W. (2023). Importance of
Molecular and Bulk Dynamics in Supramolecular Hydrogels in Dictating
Cellular Spreading. Chem. Mater..

[30] Jones C. D., Kennedy S. R., Walker M., Yufit D. S., Steed J. W. (2017). Scrolling
of Supramolecular Lamellae in the Hierarchical Self-Assembly of Fibrous
Gels. Chem..

[31] Pal A., Karthikeyan S., Sijbesma R. P. (2010). Coexisting Hydrophobic Compartments
through Self-Sorting in Rod-like Micelles of Bisurea Bolaamphiphiles. J. Am. Chem. Soc..

[32] Liu J., Schotman M. J. G., Hendrix M. M. R. M., Lou X., Marin San
Roman P. P., Voets I. K., Sijbesma R. P. (2021). Effects of Structural
Variation on the Self-assembly of Bis-urea Based Bolaamphiphiles. J. Polym. Sci..

[33] Chebotareva N., Bomans P. H. H., Frederik P. M., Sommerdijk N. A. J. M., Sijbesma R. P. (2005). Morphological Control and Molecular Recognition by
Bis-Urea Hydrogen Bonding in Micelles of Amphiphilic Tri-Block Copolymers. Chem. Commun..

[34] Pawar G. M., Koenigs M., Fahimi Z., Cox M., Voets I. K., Wyss H. M., Sijbesma R. P. (2012). Injectable Hydrogels from Segmented
PEG-Bisurea Copolymers. Biomacromolecules.

[35] Liu J., Zhang Y., van Dongen K., Kennedy C., Schotman M. J. G., Marín San Román P. P., Storm C., Dankers P. Y. W., Sijbesma R. P. (2023). Hepatic Spheroid
Formation on Carbohydrate-Functionalized
Supramolecular Hydrogels. Biomacromolecules.

[36] Hirschberg J. H. K. K., Koevoets R. A., Sijbesma R. P., Meijer E. W. (2003). Helical Supramolecular
Aggregates Based on Ureidopyrimidinone Quadruple Hydrogen Bonding. Chem. - Eur. J..

[37] Nieuwenhuizen M. M. L., de Greef T. F. A., van der Bruggen R. L. J., Paulusse J. M. J., Appel W. P. J., Smulders M. M. J., Sijbesma R. P., Meijer E. W. (2010). Self-assembly of Ureido-pyrimidinone
Dimers into One-dimensional Stacks by Lateral Hydrogen Bonding. Chem. - Eur. J..

[38] Kieltyka R. E., Pape A. C. H., Albertazzi L., Nakano Y., Bastings M. M. C., Voets I. K., Dankers P. Y. W., Meijer E. W. (2013). Mesoscale Modulation
of Supramolecular Ureidopyrimidinone-Based Poly (Ethylene Glycol)
Transient Networks in Water. J. Am. Chem. Soc..

[39] Bakker M. H., Kieltyka R. E., Albertazzi L., Dankers P. Y. W. (2016). Modular Supramolecular
Ureidopyrimidinone Polymer Carriers for Intracellular Delivery. RSC Adv..

[40] Hendrikse S. I. S., Spaans S., Meijer E. W., Dankers P. Y. W. (2018). Supramolecular
Platform Stabilizing Growth Factors. Biomacromolecules.

[41] Dankers P. Y. W., Hermans T. M., Baughman T. W., Kamikawa Y., Kieltyka R. E., Bastings M. M. C., Janssen H. M., Sommerdijk N. A. J. M., Larsen A., Van Luyn M. J. A., Bosman A. W., Popa E. R., Fytas G., Meijer E. W. (2012). Hierarchical Formation
of Supramolecular
Transient Networks in Water: A Modular Injectable Delivery System. Adv. Mater..

[42] Bastings M. M. C., Koudstaal S., Kieltyka R. E., Nakano Y., Pape A. C. H., Feyen D. A. M., van Slochteren F. J., Doevendans P. A., Sluijter J. P. G., Meijer E. W., Chamuleau S. A. J., Dankers P. Y. W. (2014). A Fast PH-switchable and Self-healing
Supramolecular Hydrogel Carrier for Guided, Local Catheter Injection
in the Infarcted Myocardium. Adv. Healthcare
Mater..

[43] Wintjens A. G. W. E., Fransen P. K. H., Lenaerts K., Liu H., van Almen G. C., van Steensel S., Gijbels M. J., de Hingh I. H. J. T., Dankers P. Y. W., Bouvy N. D. (2023). Development of a
Supramolecular Hydrogel for Intraperitoneal Injections. Macromol. Biosci..

[44] Wintjens A. G. W. E., Liu H., Fransen P. P. K. H., Lenaerts K., van Almen G. C., Gijbels M. J., Hadfoune M., Boonen B. T. C., Lieuwes N. G., Biemans R., Dubois L. J., Dankers P. Y. W., de
Hingh I. H. J. T., Bouvy N. D. (2023). Treating Colorectal Peritoneal Metastases
with an Injectable Cytostatic Loaded Supramolecular Hydrogel in a
Rodent Animal Model. Clin. Exp. Metastasis.

[45] Eding J. E. C., Vigil-Garcia M., Vink M., Demkes C. J., Versteeg D., Kooijman L., Bakker M. H., Schotman M. J. G., Dankers P. Y. W., van
Rooij E. (2024). Hydrogel Based Delivery of AntimiR 195 Improves Cardiac
Efficacy after Ischemic Injury. Adv. Ther..

[46] Vrehen A. F., Rutten M. G. T. A., Dankers P. Y. W. (2023). Development of
a Fully Synthetic
Corneal Stromal Construct via Supramolecular Hydrogel Engineering. Adv. Healthcare Mater..

[47] Vrehen A. F., van Sprang J. F., Schotman M. J. G., Dankers P. Y. W. (2024). Collagen Type
I Mimicking Peptide Additives to Functionalize Synthetic Supramolecular
Hydrogels. Mater. Today Bio.

[48] Diba M., Spaans S., Hendrikse S. I. S., Bastings M. M. C., Schotman M. J. G., van
Sprang J. F., Wu D. J., Hoeben F. J. M., Janssen H. M., Dankers P. Y. W. (2021). Engineering the Dynamics of Cell
Adhesion Cues in Supramolecular Hydrogels for Facile Control over
Cell Encapsulation and Behavior. Adv. Mater..

[49] Hendrikse S. I. S., Wijnands S. P. W., Lafleur R. P. M., Pouderoijen M. J., Janssen H. M., Dankers P. Y. W., Meijer E. W. (2017). Controlling and
Tuning the Dynamic Nature of Supramolecular Polymers in Aqueous Solutions. Chem. Commun..

[50] Cui H., Cheetham A. G., Pashuck E. T., Stupp S. I. (2014). Amino Acid Sequence
in Constitutionally Isomeric Tetrapeptide Amphiphiles Dictates Architecture
of One-Dimensional Nanostructures. J. Am. Chem.
Soc..

[51] Pashuck E.
T., Cui H., Stupp S. I. (2010). Tuning Supramolecular Rigidity of Peptide Fibers through
Molecular Structure. J. Am. Chem. Soc..

[52] Freeman R., Han M., Álvarez Z., Lewis J. A., Wester J. R., Stephanopoulos N., McClendon M. T., Lynsky C., Godbe J. M., Sangji H., Luijten E., Stupp S. I. (2018). Reversible Self-Assembly
of Superstructured Networks. Science.

[53] Leenders C. M. A., Baker M. B., Pijpers I. A. B., Lafleur R. P. M., Albertazzi L., Palmans A. R. A., Meijer E. W. (2016). Supramolecular Polymerisation
in
Water; Elucidating the Role of Hydrophobic and Hydrogen-Bond Interactions. Soft Matter.

[54] de
Greef T. F. A., Nieuwenhuizen M. M. L., Sijbesma R. P., Meijer E. W. (2010). Competitive
Intramolecular Hydrogen Bonding in Oligo (Ethylene Oxide) Substituted
Quadruple Hydrogen Bonded Systems. J. Org. Chem..

[55] Lou X., Schoenmakers S. M. C., van Dongen J. L. J., Garcia-Iglesias M., Casellas N. M., Fernández-Castaño Romera M., Sijbesma R. P., Meijer E. W., Palmans A. R. A. (2021). Elucidating Dynamic
Behavior of Synthetic Supramolecular Polymers in Water by Hydrogen/Deuterium
Exchange Mass Spectrometry. J. Polym. Sci..

[56] Freedman H. H., Dubois R. A. (1975). An Improved Williamson
Ether Synthesis Using Phase
Transfer Catalysis. Tetrahedron Lett..

[57] Altieri A., Aucagne V., Carrillo R., Clarkson G. J., D’Souza D. M., Dunnett J. A., Leigh D. A., Mullen K. M. (2011). Sulfur-Containing
Amide-Based [2] Rotaxanes and Molecular Shuttles. Chem. Sci..

[58] Gibson M.
S., Bradshaw R. W. (1968). The Gabriel
Synthesis of Primary Amines. Angew. Chemie Int.
Ed. in English.

[59] Sackett D. L., Wolff J. (1987). Nile Red as a Polarity-Sensitive Fluorescent Probe of Hydrophobic
Protein Surfaces. Anal. Biochem..

[60] Tantakitti F., Boekhoven J., Wang X., Kazantsev R. V., Yu T., Li J., Zhuang E., Zandi R., Ortony J. H., Newcomb C. J., Palmer L. C., Shekhawat G. S., de la Cruz M. O., Schatz G. C., Stupp S. I. (2016). Energy Landscapes
and Functions of Supramolecular Systems. Nat.
Mater..

[61] Edwards W., Smith D. K. (2013). Dynamic Evolving
Two-Component Supramolecular Gels
Hierarchical Control over Component Selection in Complex Mixtures. J. Am. Chem. Soc..

[62] Hirst A. R., Coates I. A., Boucheteau T. R., Miravet J. F., Escuder B., Castelletto V., Hamley I. W., Smith D. K. (2008). Low-Molecular-Weight
Gelators: Elucidating the Principles of Gelation Based on Gelator
Solubility and a Cooperative Self-Assembly Model. J. Am. Chem. Soc..

[63] Vleugels M. E. J., Bosman R., da Camino Soligo P. H., Wijker S., Fehér B., Spiering A. J. H., Rijns L., Bellan R., Dankers P. Y. W., Palmans A. R. A. (2024). Bisurea-Based Supramolecular Polymers
for Tunable Biomaterials. Chem. - Eur. J..

[64] Stimson M. M., Reuter M. A. (1945). The Ultraviolet Absorption Spectra
of Cytosine and
Isocytosine 1,2. J. Am. Chem. Soc..

[65] Hestand N. J., Spano F. C. (2017). Molecular Aggregate
Photophysics beyond the Kasha Model:
Novel Design Principles for Organic Materials. Acc. Chem. Res..

[66] Pape A. C. H., Bastings M. M. C., Kieltyka R. E., Wyss H. M., Voets I. K., Meijer E. W., Dankers P. Y. W. (2014). Mesoscale Characterization
of Supramolecular Transient Networks Using SAXS and Rheology. Int. J. Mol. Sci..

[67] Rutten M. G. T. A., Rijns L., Dankers P. Y. W. (2024). Controlled, Supramolecular Polymer
Formulation to Engineer Hydrogels with Tunable Mechanical and Dynamic
Properties. J. Polym. Sci..

[68] Rovers M. M., Rogkoti T., Bakker B. K., Bakal K. J., van Genderen M. H. P., Salmeron-Sanchez M., Dankers P. Y. W. (2024). Using a Supramolecular
Monomer Formulation Approach to Engineer Modular, Dynamic Microgels,
and Composite Macrogels. Adv. Mater..

[69] Schotman M. J. G., Fransen P.-P., Song J., Dankers P. Y. W. (2022). Tuning
the Affinity
of Amphiphilic Guest Molecules in a Supramolecular Polymer Transient
Network. RSC Adv..

[70] Schotman M. J. G., Peters M. M. C., Krijger G. C., van Adrichem I., de Roos R., Bemelmans J. L. M., Pouderoijen M. J., Rutten M. G. T. A., Neef K., Chamuleau S. A. J., Dankers P. Y. W. (2021). In Vivo Retention
Quantification
of Supramolecular Hydrogels Engineered for Cardiac Delivery. Adv. Healthcare Mater..

[71] Ma S., Meng Z., Chen R., Guan K.-L. (2019). The Hippo Pathway:
Biology and Pathophysiology. Annu. Rev. Biochem..

[72] Musah S., Wrighton P. J., Zaltsman Y., Zhong X., Zorn S., Parlato M. B., Hsiao C., Palecek S. P., Chang Q., Murphy W. L., Kiessling L. L. (2014). Substratum-Induced
Differentiation
of Human Pluripotent Stem Cells Reveals the Coactivator YAP Is a Potent
Regulator of Neuronal Specification. Proc. Natl.
Acad. Sci. U. S. A..

[73] Totaro A., Castellan M., Battilana G., Zanconato F., Azzolin L., Giulitti S., Cordenonsi M., Piccolo S. (2017). YAP/TAZ Link Cell Mechanics to Notch
Signalling to
Control Epidermal Stem Cell Fate. Nat. Commun..

[74] Klangprapan J., Souza G. R., Ferreira J. N. (2024). Bioprinting Salivary Gland Models
and Their Regenerative Applications. BDJ Open.

[75] Kutluk H., Bastounis E. E., Constantinou I. (2023). Integration
of Extracellular Matrices
into Organ-on-chip Systems. Adv. Healthcare
Mater..

[76] Merino-Casallo F., Gomez-Benito M. J., Hervas-Raluy S., Garcia-Aznar J. M. (2022). Unravelling
Cell Migration: Defining Movement from the Cell Surface. Cell Adh. Migr..

