# Supplementary material for: Tuning the Supramolecular Polymerization and Cell Response of Ureidopyrimidinone Monomers by Pushing the Hydrophobic Threshold
Source: J Am Chem Soc. 2025 Jun 11;147(25):21478–91. doi: 10.1021/jacs.5c01445 (PMC12203578; doi:10.1021/jacs.5c01445)
Supplement: Supplementary file 1 [file ja5c01445_si_001.pdf]

Supporting Information for

Tuning supramolecular polymerization and cell response of ureido-pyrimidinone monomers by pushing the hydrophobic threshold

Riccardo Bellan[1,2], Martin G. T. A. Rutten[1,2], Lou Xianwen[1,3], Chloe M. Wallace[4], Maritza M. Rovers[1,2], Andrew J. Smith[5], Marcel H. P. van Genderen[1,2], Dave J. Adams[4], Patricia Y. W. Dankers[*1,2,3]

[1] Institute for Complex Molecular Systems, Eindhoven University of Technology, Eindhoven, 5600 MB, the Netherlands

[2] Department of Biomedical Engineering, Eindhoven University of Technology, Eindhoven, 5600 MB, the Netherlands

[3] Department of Chemical Engineering and Chemistry, Eindhoven University of Technology, Eindhoven, 5600 MB, the Netherlands

[4] School of Chemistry, University of Glasgow, Glasgow, G12 8QQ, Scotland, UK

[5] Diamond Light Source Ltd, Diamond House, Harwell Science and Innovation Campus, Didcot, OX11 0DE, UK

* Corresponding author:

Patricia Y. W. Dankers, e-mail: p.y.w.dankers@tue.nl

Table of Contents

1 Experimental section S2

1.1 Materials and methods S2

1.2 Synthetic procedures S9

2 Molecular characterization S17

3 UV-Vis S38

3.1 VT-UV Vis spectra S38

3.2 UV-Vis and pH dependent UV-Vis spectra S39

4 ^{1}H-NMR spectroscopy S40

4.1 VT-NMR spectra S41

4.2 pH dependent ^{1}H-NMR spectra S44

5 Nile Red Fluorescence assay at different pHs S47

6 SAXS analysis S48

7 HDX-MS analysis S51

8 Rheological measurements S52

9 CryoTEM imaging S55

10 3D cell encapsulation experiments S57

11 References S58

1 Experimental section

1.1 Materials and methods

Unless otherwise specified, all solvents were of AR quality and purchased from Biosolve. Deuterated compounds were obtained from Cambridge Isotope Laboratories and stored over 4 Å molecular sieves except for sodium deuteroxide 40 wt % and deuterium chloride solution 35 wt % which were purchased from Merck. Dry solvents were obtained using MBraun solvent purification system (MB SPS-800). Water for aqueous samples was purified on an EMD Millipore Milli-Q Integral Water Purification System. Glassware was dried in an oven at 135 °C overnight prior to reactions under dry conditions. Reactions were followed by thin-layer chromatography (TLC) using 60-F254 silica gel plates from Merck and visualized by UV light at 254 nm and/or staining (ninhydrin, potassium permanganate). Flash column chromatography was performed on a Grace Reveleris X2 chromatography system using Reveleris Silica Flash cartridges. All **^{1}H-NMR** and **^{13}C-NMR** spectra for molecular characterization were recorded on Bruker AVANCE III HD spectrometers (400 MHz for ^{1}H NMR and 100 MHz for ^{13}C NMR). Proton chemical shifts are reported in ppm (δ) downfield from tetramethyl silane (TMS). Peak multiplicities are abbreviated as s: singlet; d: doublet; t: triplet, dt: doublet of triplet; m: multiplet. Carbon chemical shifts are reported in ppm (δ) downfield from TMS. The NMR spectra were processed using MNova x64 14.01. Liquid chromatography mass spectroscopy (LC-MS) spectra were acquired using a device consisting of multiple components: Shimadzu SCL-10 A VP system controller with Shimadzu LC-10AD VP liquid chromatography pumps (with an Alltima C18 3 u (50 × 2.1 mm) reversed-phase column and gradients of water), a Shimadzu DGU 20A3 prominence degasser, a Thermo Finnigan surveyor auto sampler, a Thermo Finnigan surveyor PDA detector and a Thermo Scientific LCW Fleet. All samples were dissolved in 1:1 H_2O:ACN in *ca*. 0.1 mg mL^{-1} concentration. **Infrared spectra** were recorded using a PerkinElmer Spectrum Two FT-IR spectrometer equipped with a PerkinElmer Universal ATR Two Accessory. All solid-state spectra were measured at room temperature from 500 cm^{-1} to 4000 cm^{-1} and were averaged over 128 scans.

General sample preparation protocol: Samples were prepared by dissolving the UPy molecules as solids either in MQ water (pH 6.8 – 7.2) or in D_2O at the desired concentration by means of sonication for one hour. The resulting samples were then equilibrated overnight while shaking (600 rpm and 20 °C). In case of concentration dependent experiments, a stock solution at the highest concentration employed in the experiment was prepared as described above and diluted at different concentrations in the proper aqueous solvent. Next, the diluted samples were further sonicated for one hour and equilibrated overnight under shaking (600

rpm at 20 °C). For pH or pD dependent measurements, samples were prepared by dissolving the materials as solids either in MQ water at pH 3.0, 7.0 or 12 or in D_2O at pD 3.0, 7.0 or 12 according to the above reported conditions.

Solution FT-IR: FT-IR spectra in solution were recorded on a Perkin Elmer Spectrum Two FT-IR spectrometer. Liquid samples were measured at room temperature from 2000 cm^{-1} to 1000 cm^{-1} over 128 scans and 4 cm^{-1} resolution in a BaF_2 cell with a spacer of 0.06 mm.

UV-Vis spectroscopy: UV-Vis measurements were carried out on a JASCO V-750-UV-Vis spectrometer equipped with a JASCO Peltier ETCR-762 with a temperature range of 263 K to 383 K and external circulating thermostat JASCO CTU-100 using the following parameters unless otherwise specified: response time: 0.24 sec, bandwidth: 1.0 nm. All spectroscopic measurements were performed using sealed Hellma quartz cuvettes with an optical path length of 1.0 mm and 9.0 mm metal spacer as heat bridge. Variable temperature UV (VT-UV) measurements were carried out by heating the sample from 10 °C to 80 °C at a rate of 1.0 °C min^{-1} using the same instrument and the following parameters: response time: 0.24 sec, bandwidth: 1.0 nm, heating rate: 1.0 °C min^{-1}, data pitch: 1.0 °C, temperature interval: 5.0 °C. All measurements were baseline corrected and averaged over three measurements. The samples were placed in the sample holder at 10 °C for 1 hour before heating to 80 °C to ensure comparable thermal history. The cooling curves monitored at 280 nm obtained were obtained by controlled cooling from 80 °C to 10 °C at a rate of 0.2 °C·min^{-1}. Measurements were performed after heating the sample at 80 °C for 1 hour.

Fluorescence spectroscopy: The self-assembly of the UPy molecules were assessed by incorporation of the hydrophobic solvatochromic probe Nile Red (9-diethylamino-5-benzo[α]phenoxazinone), which exhibits a blue shift emission upon inclusion in hydrophobic environments. Nile Red was dissolved at 625 μM in ethanol and further diluted into solutions to a final concentration of 250 nM. As a result, all samples contain 0.04 v/v % ethanol, which is expected to not interfere with the self-assembly process. The aqueous solutions of the UPy molecules (concentration: 0.1 μM to 100 μM) mixed with Nile Red (250 nM) were excited at 550 nm and the spectra were recorded over a wavelength range of 580–800 nm using an Agilent Cary Eclipse fluorescence spectrophotometer. Blue shifts were calculated by subtracting the emission wavelength of Nile Red in MQ water (λ_{em} = 658 nm) from the emission wavelength of the sample. Blue shifts were plotted against concentrations to determine the critical aggregation concentration of each molecule.

Variable Temperature (VT-NMR) and pH dependent ^{1}H-NMR spectroscopy: VT-NMR and pH dependent ^{1}H-NMR experiments were carried out on a Varian Unity Inova 500 MHz Spectrometer equipped with a 5 mm AutoX DB probe. VT-NMR experiments were performed by heating the samples from 25 °C to 80 °C using. Samples were prepared at 500 μM in D_2O and equilibrated overnight at room temperature. Anhydrous DMSO was diluted in D_2O at 100 mM and further diluted into solutions as internal standard for kinetic analysis. As a result, each sample contains 0.1 v/v % of the diluted DMSO solution (100 mM in D_2O) which is expected not to interfere with the assembly process. Every sample was equilibrated 15 minutes before every measurement at the target temperature and all the spectra were acquired over 128 scans with a relaxation delay of 10 sec. pH dependent ^{1}H-NMR experiments were acquired over the same set of parameters described above.

Small Angle X-Ray Scattering (SAXS):[1,2] SAXS measurements were performed at the Diamond Light Source, Didcot, at the I22 beamline under experiment number SM33006-1. The beamline operated at an energy of 12.4 keV and the detector distance was set to 8.750 m, allowing a final Q range of 0.0017 to 0.203 Å^{-1}. Samples were prepared in glass vials and loaded into borosilicate glass capillaries (1.56 mm internal diameter) using a 1 mL syringe equipped with a 21G needle. For each sample, 1000 x 10 ms frames were collected and averaged to achieve a total exposure of 10 s.

The raw data was processed using the Dawn Science software (version 2.27),[1] according to a standard I22 pipeline.[2] As part of the processing, the backgrounds (the scattering from the solvent) were subtracted from the raw 2D SAXS data and a full azimuthal integration was performed to reduce the data to a I vs *q* plot. The plots were then fitted to structural models in the SasView software (5.0.5)

To calculate the X-ray scattering length densities (SLDs) for all samples, the NIST neutron activation and scattering calculator was used (https://www.ncnr.nist.gov/resources/activation/), assuming a density of 1.55 g/cm^3 for the monomers.

HDX-MS measurements:[3] HDX–MS measurements were carried out using a XevoTM G2 QTof mass spectrometer (Waters) with a capillary voltage of 2.7 kV, a sampling cone voltage of 20 V, and an extraction cone voltage of 4.0 V. The source temperature was set at 100 °C, the desolvation temperature was set at 400 °C, the cone gas flow was set at 10 L/h, and the desolvation gas flow was set at 100 L/h. The sample solutions subjected to HDX were introduced into the mass spectrometer using a Harvard syringe pump (11 Plus, Harvard Apparatus) at a flow rate of 50 μL/min. The UPy samples at a concentration of 2.5 mM in MQ water were diluted 25 times in D_2O (including 0.5 mM sodium acetate to facilitate detection), resulting in a final concentration of 100 μM. The samples were stored at room temperature

during the experiment. MS spectra of the supramolecular assemblies in water were recorded at a number of given time points after dilution. The distributions of the various deuterated species of the UPy molecules were determined based on the isotope patterns calculated from Bruker Compass IsotopePattern software.

Cryo-TEM: Vitrified thin films for CryoTEM analysis were prepared using an automated vitrification robot (FEI Vitrobot Mark IV) by plunge vitrification in liquid ethane. Before vitrification, a 200-mesh copper grid covered with a Quantifoil R 2/2 holey carbon film (Quantifoil Micro Tools GmbH) was surface plasma treated for 40 seconds using a Cressington 208 carbon coater. CryoTEM imaging was carried out on the Glacios (Thermo Fisher), equipped with a field emission gun (X-FEG), Ceta 16M camera and a Falcon 4i direct electron detector. The microscope was operated at 200 kV acceleration voltage in bright-field TEM at a nominal magnification of 6.500× and a dose rate of 2 $e^-/Å^2 \cdot s$; or at 24.000× magnification and a dose rate of 4 $e^-/Å^2 \cdot s$; both with a 1s image acquisition time.

Hydrogel formation: UPy hydrogels were prepared by following a previously described procedure.[4] The solid UPy compounds were weighed into 1.5 mL clean, separate glass vials. The UPy-Cn compounds were dissolved in adequate amount of basic PBS (i.e. PBS with 80 mM NaOH). Parafilm was applied around the lid to prevent evaporation. The samples were heated for ~ 30 min at 70 °C in a water bath. During and after heating, the samples were vortexed several times for 10 seconds. Thereafter, the combined solution was neutralized using 1 M HCl in MQ water. UPy-PEG-UPy was dissolved in PBS. Parafilm was applied around the lid to prevent evaporation. The samples were heated for ~ 90 min at 70 °C in a water bath. During and after heating, the samples were vortexed several times for 10 seconds. Final hydrogels were formed by mixing the UPy solution with UPy-PEG-UPy in a 1:1 volume ratio. In cases where no UPy-PEG-UPy was used, the UPy solution was diluted with PBS. This final solution was pipetted in a 96 well-plate and allowed to form a hydrogel for 24 h at 37 °C.

Table S1. Hydrogels composition for **UPy** : **BF** = 80 : 1 ratio.

Sample ID	UPy [mM]	UPy [mg/mL]	BF [mM]	BF [mg/mL]	UPy : BF	Total UPy [mM]
**C6-O + BF**	9.75	8.86	0.12	1.35	80 : 1	9.87
**C8-O + BF**	9.75	9.13	0.12	1.35	80 : 1	9.87
**C10-O + BF**	9.75	9.41	0.12	1.35	80 : 1	9.87
**C12-O + BF**	9.75	9.68	0.12	1.35	80 : 1	9.87

Table S2. Hydrogels composition for **UPy** : **BF** = 9 : 1 ratio.

Sample ID	UPy [mM]	UPy [mg/mL]	BF [mM]	BF [mg/mL]	UPy : BF	Total UPy [mM]
**C6-O + BF**	8.88	8.08	0.99	10.96	9 : 1	9.87

**C8-O + BF**	8.88	8.33	0.99	10.96	9 : 1	9.87
**C10-O + BF**	8.88	8.57	0.99	10.96	9 : 1	9.87
**C12-O + BF**	8.88	8.82	0.99	10.96	9 : 1	9.87

Table S3. Hydrogels composition for **UPy** : **BF** = 1 : 1 ratio.

Sample ID	UPy [mM]	UPy [mg/mL]	BF [mM]	BF [mg/mL]	UPy : BF	Total UPy [mM]
**C6-O + BF**	4.93	4.49	4.93	54.8	1 : 1	9.87
**C8-O + BF**	4.93	4.62	4.93	54.8	1 : 1	9.87
**C10-O + BF**	4.93	4.76	4.93	54.8	1 : 1	9.87
**C12-O + BF**	4.93	4.90	4.93	54.8	1 : 1	9.87

Rheology: Rheological measurements were carried out on a TA Instruments Discovery Hybrid Rheometer 3 or 30 equipped with an 8 mm flat steel plate-plate geometry which was slowly lowered until it made full contact with the gel, yielding a typical gap height of 350 – 850 μm for gels composed of UPy and UPy-PEG-UPy. For the (near) liquid-like samples of only UPy lower gaps were needed to fully cover the geometry (75 – 650 μm). Low viscosity silicon oil (47 V 100, RHODORSIL®) was used around the hydrogel to minimize sample drying. Samples were loaded at 37 °C after which the complex modulus G* (γ = 1.0 %, ω = 1 rad/s) was measured for 5 minutes to ensure that samples were at a stable plateau modulus and were and not altered or damaged during loading. Subsequent frequency sweep measurements were performed at ω = 100 rad s^{-1} to 0.1 rad s^{-1}, at a strain of γ = 1.0 %. Stress-relaxation was measured by applying a strain of γ = 7.5 % with a strain rise time of 0.09 s and monitoring the stress for 1000 seconds. The data were normalized using the stress at 1 second as starting point. Strain-sweep measurements were performed at strains between γ = 0. 01 % and γ = 1000 % with a frequency of ω =1 rad s^{-1}. Continuous time relaxation spectra were calculated by averaging two stress relaxation spectra between 0.14 s and 981.4 s. The resulting G(t) was then fitted via the rheology TRIOS software using:

$$G(t) = G_e + \int_{-\infty}^{+\infty} Hln(\tau) e^{-t/\tau} d \ln \tau$$

The obtained H_i values were then plotted vs τ and normalized using the highest and lowest H_i value.

2D cell culture: The human normal dermal fibroblasts (hNDFs; Lonza) were cultured in DMEM Advanced medium (Gibco) supplemented with 10 % v/v fetal bovine serum (FBS), 1 % v/v penicillin-streptomycin (P/S), and 1% v/v Gluta MAX at 37 °C and 5% CO_2. 2D cell culture of

hNDFS on the hydrogel samples was carried out by preparing the gels (for all conditions 1.0 w/v % + 1.0 mM UPy-cRGD) in 15-well Ibidi slides (10 µL per well). After overnight incubation the hydrogels were UV-sterilised for 20 minutes. After this, hNDF cells were seeded at a density of 3000 cells per well.

3D Cell Culture: 3D cell culture of hNDFS in the hydrogel samples was carried out by preparing the gels (for all conditions 1.0 w/v% + 1.0 mM UPy-cRGD) in 15-well Ibidi slides (10 µL per well). Pre-solutions of **BF** and **Cn-O** were prepared separately. Each UPy monomer with UPy-cRGD was dissolved at twice the desired final concentration at 70 °C for 20 minutes under stirring conditions in an alkaline solution (80 mm NaOH in PBS). The resulting solution was neutralized by the addition of acid (1 M HCl) and then diluted once with complete medium. The bifunctional molecule was dissolved at three times the desired end concentration at 70 °C in PBS for 1 hour. Afterwards, both solutions were transferred from a glass vial to a sterile Eppendorf tube. The solutions were UV-sterilized for 20 minutes. The BF solution was diluted to the desired end concentration with cells in medium to obtain 3000 cells per well. The two solutions were mixed 1:1 and pipetted into the well. After 3 hours of gelation, medium was carefully added on top of the gel, and the cells were cultured for 3 days.

Table S4. Hydrogels composition for 2D cell culture at **UPy** : **BF** = 80 : 1.

Sample ID	UPy [mM]	UPy [mg/mL]	BF [mM]	BF [mg/mL]	UPy-cRGD [mM]	UPy : BF	Total UPy [mM]
**C6-O + BF**	8.75	7.95	0.12	1.12	1	80 : 1	9.87
**C8-O + BF**	8.75	8.19	0.12	1.12	1	80 : 1	9.87
**C10-O + BF**	8.75	8.45	0.12	1.12	1	80 : 1	9.87
**C12-O + BF**	8.75	8.69	0.12	1.12	1	80 : 1	9.87

Table S5. Hydrogels composition for 3D cell culture at **UPy** : **BF** = 84 : 1.

Sample ID	UPy [mM]	UPy [mg/mL]	BF [mM]	BF [mg/mL]	UPy-cRGD [mM]	UPy : BF	Total UPy [mM]
**C6-O + BF**	6.58	5.98	0.09	0.84	1	84 : 1	7.67
**C8-O + BF**	6.58	6.16	0.09	0.84	1	84 : 1	7.67
**C10-O + BF**	6.58	6.35	0.09	0.84	1	84 : 1	7.67
**C12-O + BF**	6.58	6.53	0.09	0.84	1	84 : 1	7.67

Cell staining and Imaging: After the culture period the media was removed and the samples were washed three times with PBS. The hNDFs were then fixed using 3.7 % paraformaldehyde (formaline 37 %, Merck) in PBS for 10 minutes whereafter 0.5 % Triton X-100 in PBS was added for another 10 minutes to permeabilize the cells. Cells were washed twice with PBS and blocked with 10 % goat serum in 0.05 % Triton X-100 in PBS for 30 minutes. Next for 2D hydrogels, the cells were incubated with the primary antibodies anti-YAP1 (1:100; Abcam ab52771) and anti-vimentin (1:300; Abcam ab20346) diluted in 2 % goat serum in 0.05 %

Triton X-100 in PBS overnight at 4 °C. Thereafter, the cells were washed three times with PBS and incubated with the secondary antibodies anti-rabbit Alexa 647 (for YAP) and anti-mouse Alexa 555 (for vimentin) (both 1:250) at room temperature for 2 hours. The solution was removed and the nuclei was stained with DAPI (1:250) for 10 minutes, whereafter the cells were washed three times with PBS. For 3D hydrogels, cells were incubated with phalloidin 488 (1:300) for 2 hours and the nuclei was stained with DAPI (1:250) for 10 minutes. Imaging was performed using a Leica TCS SP8 X confocal microscope (Leica Microsystems). Images were processed in ImageJ to create max-projection images of the original z-stacks and a custom-made cell profiler pipeline was used to quantify percentage area covered by cells, and the intensity of YAP expression inside the cytoplasm and inside the nuclei.

1.2 Synthetic procedures

Synthesis of precursor 1-(6-isocyanatohexyl)-3-(6-methyl-4-oxo-1,4-dihydropyrimidin-2-yl)urea (2):

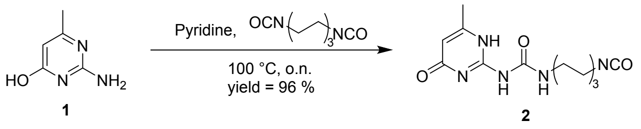

Scheme S1. Synthesis of precursor **2**.

A round-bottomed flask was charged with 2-amino-4-hydroxy-4-methylpyrimidine (**8**) (5.0 g; 40 mmol), hexamethylene diisocyanate (100 mL; 680 mmol) and pyridine (1.0 mL; 12 mmol). The resulting mixture was heated to 100 °C overnight under argon atmosphere while stirring. After 16 hours, the mixture was cooled to room temperature, diluted with 57.0 mL of heptane and filtered over a Buchner filter. During the filtration, the Buchner filter was covered with a large funnel with a N_2 flow to protect the final product from humidity. The product was washed with heptane (5 × 43 mL) and hexane (2 × 43 ml). The product was finally dried under vacuum to provide desired compound (11.3g; 38 mmol) in 96% yield. ^{1}H-NMR(400 MHz, $CDCl_3$): δ 13.11 (s, 1H), 11.86 (s, 1H), 10.19 (s, 1H), 5.82 (s, 1H), 3.30-3.23 (m, 4H), 2.23 (s, 3H), 1.65-1.58 (m, 6H), 1.48 – 1.33 (m, 2H). ^{13}C-NMR (100 MHz, $CDCl_3$): δ 173.22, 156.75, 154.86, 148.43, 106.85, 43.03, 39.93, 31.34, 29.45, 26.38, 26.32, 19.09. NMR results in agreement with the literature.[5]

Synthesis of Cn-O derivatives:

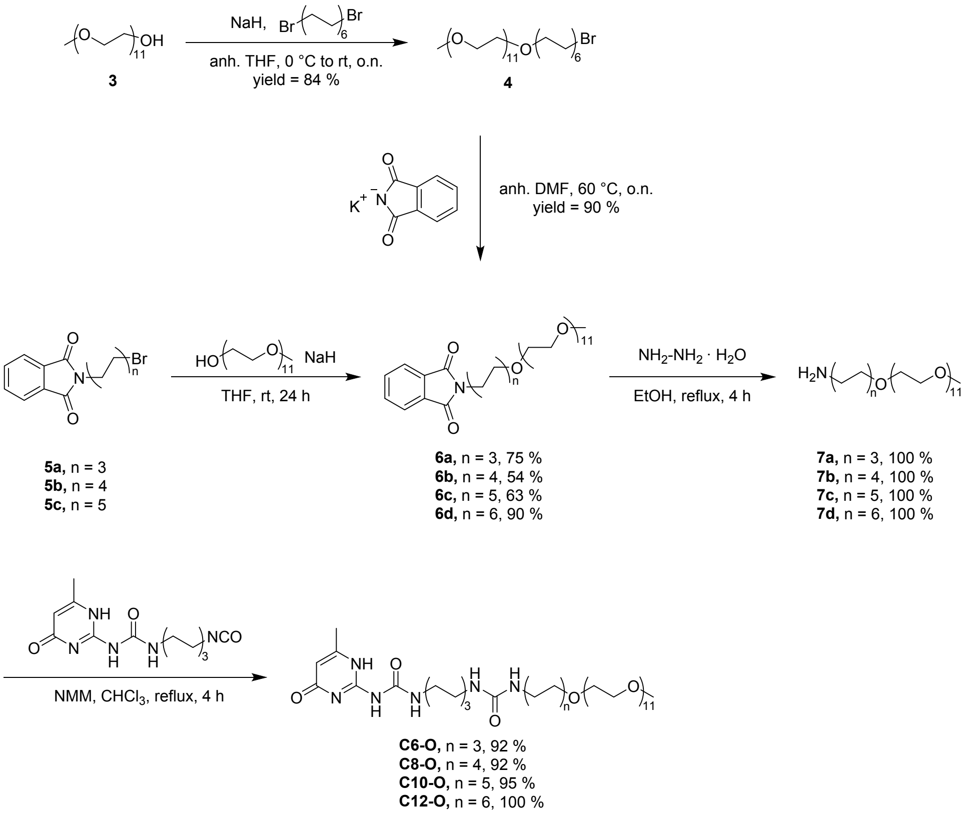

Scheme S2. Synthetic scheme towards **C6-O**, **C8-O**, **C10-O** and **C12-O**.

47-bromo-2,5,8,11,14,17,20,23,26,29,32,35-dodecaoxaheptatetracontane (4)

An oven-dried round-bottomed flask (dried at 135 °C) was charged with mOEG$_{11}$-OH (**3**) (430 mg, 0.83 mmol). **3** was dried over toluene thrice and under high vacuum for 2 hours. The dry **3** was dissolved in anhydrous THF (1.5 mL) and cooled in an ice bath while stirring under argon atmosphere. NaH (50 mg, 1.25 mmol) was added in portion and the resulting mixture was stirred vigorously for 2 hours while warming to rt. Next, 1,12-dibrododecane (1.2 g, 3.7 mmol) was added all in once. The resulting mixture was allowed to stir overnight. After 16 hours the mixture was quenched with H_2O (1 mL). The solvent was evaporated under vacuum and the aqueous solution was poured into a separating funnel along with brine (10 mL). The aqueous phase was extracted with DCM (3 × 50 mL) and the combined organic layers were collected, dried over $MgSO_4$, filtered and concentrated in vacuo. The crude product was purified through normal-phase flash column chromatography (eluent: DCM/MeOH/dimethoxyetane (DME) = 9/0.5/0.5), yielding compound **4** as colourless oil (534 mg, 0.70 mmol) in 84 % yield. ^{1}H NMR (400 MHz, $CDCl_3$) δ 3.63-3.59 (m, 38H), 3.56-3.50 (m, 4H), 3.43 – 3.33 (m, 7H), 1.82 (p, *J* = 8.0 Hz, 2H), 1.54 (p, *J* = 8.0 Hz, 2H), 1.43 – 1.34 (m, 2H), 1.30-1.23 (m, 16H). ^{13}C NMR (100 MHz, $CDCl_3$) δ 72.05, 71.64, 70.74, 70.72, 70.69, 70.63, 70.16, 59.14, 34.15, 32.94, 29.74,

29.67, 29.64, 29.61, 29.58, 29.52, 28.86, 28.27, 26.19. LC-MS: calc. *m/z*: 762.41; found *m/z*: $[M+H]^+$ = 764.75, $[M+Na]^+$ = 787.67.

2-(2,5,8,11,14,17,20,23,26,29,32,35-dodecaoxaheptatetracontan-47-yl)isoindoline-1,3-dione (6d)

A round-bottomed flask was charged with compound **4** (500 g; 0.65 mmol; 1eq.), N,N-dimethylformamide (DMF, 2.0 mL) and potassium phthalimide (150 mg, 0.81 mmol; 1.2 eq.). The mixture reaction was stirred for 16 hours at 60 °C. After cooling to room temperature, the solvent was removed under reduced pressure and the crude was dissolved in DCM (30 mL). The organic layer was washed with $NaOH_{aq}$ 0.1 M (2 × 20 mL) and the aqueous phase was back extracted with fresh DCM (2 × 20 mL). The combined organic layers were dried over anhydrous $MgSO_4$ and concentrated under reduced pressure. The crude product was purified through normal-phase column chromatography (eluent: DCM/MeOH/DME = 95/2.5/2.5) to provide compound **6d** as a yellowish oil (486 mg, 0.59 mmol) in 90 % yield. ^{1}H NMR (400 MHz, $CDCl_3$) δ 7.82 (dd, *J* = 8.0, 4.0 Hz, 2H), 7.69 (dd, *J* = 8.0, 4.0 Hz, 2H), 3.65-3.61 (m, 42H), 3.57-3.52 (m, 4H), 3.43 (t, *J* = 8.0 Hz, 2H), 3.36 (s, 3H), 1.65 (p, *J* = 8.0 Hz, 2H), 1.54 (p, *J* = 8.0, 4.5 Hz, 2H), 1.30 – 1.23 (m, 16H). ^{13}C NMR (100 MHz, $CDCl_3$) δ 168.61, 133.96, 132.34, 123.28, 72.08, 71.70, 70.76, 70.72, 70.67, 70.19, 59.18, 38.22, 29.77, 29.72, 29.69, 29.62, 29.33, 28.74, 27.00, 26.23. LC-MS: calc. *m/z*: 829.52; found *m/z*: $[M+NH_4]^+$ = 847.83, $[M+Na]^+$ = 852.58.

General procedure 1 for the synthesis of 6a-c:

An oven-dried round-bottomed flask (dried at 135 °C) was charged with **3** (0.50 – 1.46 mmol; 1 eq.) and water was azeotropically removed with toluene under vacuum twice. Anhydrous THF (1.0 mL·mmol of **3**) was added and the solution was cooled in an ice bath for 30 min. NaH (22 mg, 0.55 mmol, 1.1 eq.) was added in portion and the resulting mixture was stirred vigorously for 2 hours while warming to rt. *N*-(6-Bromohexyl)phthalimide (**5a-c**) (2 eq.) was dissolve in anh. THF (0.5 – 1.0 mL) in a separate round bottom flask and added to the $mOEG_{11}$-OH/NaH solution. The resulting mixture was allowed to stir overnight and then quenched with H_2O (~ 1.0 mL). The solvent was evaporated under high vacuum. The crude was dissolved in water and brine and poured in a separating funnel along with DCM. The aqueous phase was extracted with DCM (3 × 40 - 60 mL). The combined organic layers were dried over $MgSO_4$ and concentrated under vacuum to provide a crude product which was purified through normal phase column chromatography (eluent: DCM/MeOH/DME = 9/0.5/0.5).

2-(2,5,8,11,14,17,20,23,26,29,32,35-dodecaoxahentetracontan-41-yl)isoindoline-1,3-dione (6a)

The synthesis of **6a** was performed following the general procedure 1, providing **6a** (283 mg, 0.38 mmol) as yellowish oil in 75 % yield. ^{1}H NMR (400 MHz, $CDCl_3$) δ 7.84 (dd, *J* = 8.0, 4.0 Hz, 2H), 7.70 (dd, *J* = 8.0, 4.0 Hz, 2H), 3.69-3.61 (m, 42H), 3.57-3.53 (m, 4H), 3.43 (t, *J* = 8.0 Hz, 2H), 3.38 (s, 3H), 1.67 (p, *J* = 8.0 Hz, 2H), 1.57 (p, *J* = 8.0 Hz, 2H) 1.38-1.35 (m, 4H). ^{13}C NMR (100 MHz, $CDCl_3$) δ 168.58, 133.99, 132.31, 123.30, 72.09, 71.41, 70.76, 70.73, 70.67, 70.20, 59.18, 38.11, 29.61, 28.71, 26.85, 25.84. LC-MS: calc.: *m/z* 745.42, found: $[M+H]^+$ = 746.67, $[M+Na]^+$ = 768.50.

2-(2,5,8,11,14,17,20,23,26,29,32,35-dodecaoxatritetracontan-43-yl)isoindoline-1,3-dione (6b)

The synthesis of **6b** was performed following the general procedure 1, providing **6b** (612 mg; 0.79 mmol) as a yellowish oil in 54 % yield. ^{1}H NMR (400 MHz, $CDCl_3$) δ 7.84 (dd, *J* = 8.0, 4.0 Hz, 2H), 7.71 (dd, *J* = 8.0, 4.0 Hz, 2H), 3.66 - 3.64 (m, 42H), 3.59 – 3.52 (m, 4H), 3.43 (t, *J* = 8.0f Hz, 2H), 3.38 (s, 3H), 1.67 (p, 8H), 1.56 (p, *J* = 8.0 Hz, 2H), 1.38 – 1.26 (m, 8H). ^{13}C NMR (100 MHz, $CDCl_3$) δ 175.02, 140.43, 138.76, 129.73, 78.52, 78.03, 77.19, 77.16, 77.10, 76.64, 65.61, 60.06, 44.61, 36.16, 35.88, 35.70, 35.16, 33.38, 32.58. LC-MS: calc.: *m/z* = 773.46, found = 775.5 $[M+H]^+$ and 796.83 $[M+Na]^+$.

2-(2,5,8,11,14,17,20,23,26,29,32,35-dodecaoxaheptatriacontan-37-yl)isoindoline-1,3-dione (6c)

The synthesis of **6c** was performed following the general procedure 1, providing **6c** (292 mg; 0.37 mmol) as a yellowish oil in 63 % yield. ^{1}H NMR (400 MHz, $CDCl_3$) δ 7.83 (dd, *J* = 8.0, 4.0 Hz, 2H), 7.70 (dd, *J* = 8.0, 4.0 Hz, 2H), 3.68 - 3.61 (m, 42H), 3.57 - 3.52 (m, 4H), 3.42 (t, *J* = 8.0 Hz, 2H), 3.37 (s, 3H), 1.65 (p, *J* = 8.0 Hz, 2H), 1.55 (p, *J* = 8.0 Hz, 2H), 1.31 - 1.25 (m, 14H). ^{13}C NMR (100 MHz, $CDCl_3$) δ 168.58, 133.95, 132.31, 123.26, 72.06, 71.65, 70.74, 70.70, 70.65, 70.16, 59.16, 38.19, 29.74, 29.61, 29.55, 29.29, 28.71, 26.97, 26.18. LC-MS: calc. *m/z*: 801.49, found *m/z*: 824.83 $[M+Na]^+$.

General procedure 2 for the synthesis of 7a-d:

Hydrazine hydrate (50–60%, 2.0 mmol, 162 μL, 14 eq.) was added to a solution of compound **6a-d** (100 mg, 0.14 mmol – 0.46 mmol) in ethanol (14 mL · mmol of **6a-d**) and the resulting solution was heated at reflux for 4 h. After cooling to room temperature, the mixture was dissolved in DCM (~ 50 mL), filtered through a glass filter and washed with 15% aq. NaOH (90 - 120 mL). The aqueous phase was back extracted twice with fresh DCM (3 × 50 – 80 mL) and

the organic layers were dried over $MgSO_4$. The organic phase was concentrated in vacuo to provide a crude **7a-c** that was used without further purification in the next step.

2,5,8,11,14,17,20,23,26,29,32,35-dodecaoxahentetracontan-41-amine (7a)

The synthesis of **7a** was performed following the general procedure 2, providing **7a** (86 mg; 0.14 mmol) as a transparent wax in quantitative yield. The obtained crude was used without further purification in the next step. ^{1}H NMR (400 MHz, $CDCl_3$) δ 3.65 (m, 42H), 3.58 - 3.53 (m, 4H), 3.44 (t, J = 8.0 Hz, 2H), 3.37 (s, 3H), 2.70 (br, 2H), 1.58 (p, J = 8.0 Hz, 2H), 1.44 (p, J = 8.0 Hz, 2H), 1.38 – 1.30 (m, 4H).^{13}C NMR (100 MHz, $CDCl_3$) δ 72.08, 71.54, 70.77, 70.72, 70.67, 70.22, 59.18, 429.75, 26.88, 26.13. LC-MS: calc.: *m/z* 615.42, found: $[M+H]^+$ = 616.75.

2,5,8,11,14,17,20,23,26,29,32,35-dodecaoxatritetracontan-43-amine (7b)

The synthesis of **7b** was performed following the general procedure 2, providing **7b** (508 mg; 0.79 mmol) as a transparent wax in quantitative yield. The obtained crude was used without further purification in the next step. ^{1}H NMR (400 MHz, $CDCl_3$) δ 3.65 - 3.63 (m, 42H), 3.59 - 3.54 (m, 4H), 3.45 (t, *J* = 8.0 Hz, 2H), 3.38 (s, 3H), 2.69 (t, *J* = 8.0 Hz, 2H), 1.57 (p, *J* = 8.0 Hz, 2H), 1.44 (p, *J* = 8.0 Hz, 2H), 1.31 (m, 8H). ^{13}C NMR (100 MHz, $CDCl_3$) δ 72.02, 71.56, 70.72, 70.68, 70.66, 70.60, 70.14, 59.11, 42.21, 29.68, 29.49, 29.47, 26.89, 26.11. LC-MS: calc.: *m/z* 643.45, found: $[M+H]^+$ = 644.75.

2,5,8,11,14,17,20,23,26,29,32,35-dodecaoxapentatetracontan-45-amine (7c)

The synthesis of **7c** was performed following the general procedure 2, providing **7c** (242 mg; 0.36 mmol) as a transparent wax in quantitative yield. The obtained crude was used without further purification in the next step. ^{1}H NMR (400 MHz, $CDCl_3$) δ 3.65 - 3.62 (m, 42H), 3.58 - 3.53 (m, 4H), 3.43 (t, *J* = 8.0 Hz, 2H), 3.37 (s, 3H), 2.67 (t, *J* = 8.0 Hz, 2H), 1.56 (p, *J* = 8.0 Hz, 2H), 1.46 - 1.39 (m, 4H), 1.32 - 1.24 (m, 14H). ^{13}C NMR (100 MHz, $CDCl_3$) δ 72.07, 71.67, 70.77, 70.74, 70.71, 70.66, 70.19, 59.17, 42.40, 33.97, 29.76, 29.69, 29.66, 29.61, 29.60, 27.02, 26.21. LC-MS: calc. *m/z* 671.48, found: $[M+H]^+$ = 672.75.

2,5,8,11,14,17,20,23,26,29,32,35-dodecaoxapentatetracontan-45-amine (7d)

The synthesis of **7d** was performed following the general procedure 2, providing **7d** (322 mg; 0.46 mmol) as a transparent wax in quantitative yield. The obtained crude was used without further purification in the next step. ^{1}H NMR (400 MHz, $CDCl_3$) δ 3.67 – 3.60 (m, 42H), 3.59 – 3.52 (m, 4H), 3.43 (t, *J* = 7.0 Hz, 2H), 3.37 (s, 3H), 2.68 (t, *J* = 7.0 Hz, 2H), 1.56 (p, *J* = 7.0 Hz, 2H), 1.45 (p, *J* = 8.0 Hz, 2H), 1.26 (m, 16H). ^{13}C NMR (100 MHz, $CDCl_3$) δ 72.08, 71.68, 70.77, 70.75, 70.72, 70.66, 70.19, 59.18, 42.27, 33.65, 31.06, 29.77, 29.74, 29.71, 29.61, 27.02, 26.22. LC-MS: calc. *m/z*: 699.51; found *m/z*: $[M+H]^+$ = 700.75.

General procedure 3 for the synthesis of Cn-O:

A round-bottomed flask was charged with **7a-d** (1.1 eq.), *N*-methyl morpholine (1.7 eq.) and $CHCl_3$ (20 mL · mmol of **2**). Compound **2** (0.13 – 0.72 mmol; 1.0 eq.) was added and the resulting mixture was stirred at reflux for 4 hours under argon atmosphere. After 4 hours the mixture was cooled down at rt and stirred for 48 hours. Next, the reaction mixture was concentrated under vacuum and the crude product was purified through normal-phase column chromatography (eluent: DCM/MeOH/DME = 8/2/2). The column fractions containing the compound of interest were pulled together and evaporated to dryness. Finally, the pure compounds were isolated after freeze-drying from MQ water.

1-(6-methyl-4-oxo-1,4-dihydropyrimidin-2-yl)-3-(43-oxo-2,5,8,11,14,17,20,23,26,29,32,35-dodecaoxa-42,44-diazapentacontan-50-yl)urea (C6-O)

The synthesis of **C6-O** was performed following the general procedure 3, providing **C6-O** (100.0 mg, 0.11 mmol) as a white solid in 92 % yield. ^1H NMR (400 MHz, $CDCl_3$) δ 13.15 (s, 1H), 11.82 (s, 1H), 10.07 (s, 1H), 5.82 (s, 1H), 4.80 (t, *J* = 4.0 Hz, 1H), 4.61 (d, *J* = 4.0 Hz, 1H), 3.65 - 3.61 (m, 42H), 3.56 - 3.53 (m, 4H), 3.43 (t, *J* = 8.0 Hz, 2H), 3.37 (s, 3H), 3.23 (dd, *J* = 12 Hz*,* J = 4.0 Hz, 2H), 3.14 (dd, *J* = 12 Hz, *J* = 4.0, Hz 4H), 2.23 (s, 3H), 1.56 (m, 4H), 1.48 (m, 4H), 1.34 (m, 8H). ^{13}C NMR (100 MHz, $CDCl_3$) δ 173.34, 158.65, 156.65, 154.86, 148.59, 106.71, 72.06, 71.40, 70.76, 70.74, 70.70, 70.65, 70.19, 59.17, 40.45, 40.25, 39.74, 30.36, 30.01, 29.60, 29.44, 26.78, 26.41, 26.34, 25.95, 19.09. LC-MS: calc.: *m/z* 908.57, found: $[M+H]^+$ = 909.67, $[M+2H]^{2+}$ = 456.08.

1-(6-methyl-4-oxo-1,4-dihydropyrimidin-2-yl)-3-(45-oxo-2,5,8,11,14,17,20,23,26,29,32,35-dodecaoxa-44,46-diazadopentacontan-52-yl)urea (C8-O)

The synthesis of **C8-O** was performed following the general procedure 3, providing **C8-O** (620 mg; 0.66 mmol) as a white solid in 92 % yield. ^1H NMR (400 MHz, $CDCl_3$) δ 13.16 (br, 1H), 11.82 (br, 1H), 10.07 (br, 1H), 5.83 (s, 1H), 4.86 (t, *J* = 4.0 Hz, 1H), 4.68 (t, *J* = 4.0f Hz, 1H), 3.68 - 3.61 (m, 42H), 3.58 - 3.54 (m, 4H), 3.44 (t, *J* = 8.0 Hz, 2H), 3.38 (s, 3H), 3.24 (q, *J* = 8.0 Hz, 2H), 3.15 (dt, *J* = 8.0, 4.0 Hz, 4H), 2.24 (s, 3H), 1.57 (p, *J* = 8.0 Hz, 4H), 1.47 (p, *J* = 8.0 Hz, 5H), 1.40 – 1.23 (m, 12H). ^{13}C NMR (101 MHz, $CDCl_3$) δ 173.35, 158.69, 156.62, 154.85, 148.61, 106.67, 72.04, 71.57, 70.73, 70.71, 70.67, 70.62, 70.16, 59.14, 40.56, 40.22, 39.72, 30.45, 29.96, 29.69, 29.47, 29.39, 26.96, 26.38, 26.30, 26.11, 19.07. LC-MS: calc.: *m/z* 936.60, found: $[M+2H]^{2+}$ = 478.33.

1-(6-methyl-4-oxo-1,4-dihydropyrimidin-2-yl)-3-(45-oxo-2,5,8,11,14,17,20,23,26,29,32,35-dodecaoxa-44,46-diazadopentacontan-52-yl)urea (C10-O)

The synthesis of **C10-O** was performed following the general procedure 3, providing **C10-O** (302 mg; 0.32 mmol) as a white solid in 95 % yield. ^{1}H NMR (400 MHz, $CDCl_3$) δ 13.16 (s, 1H), 11.83 (s, 1H), 10.08 (s, 1H), 5.83 (s, 1H), 4.72 (t, *J* = 4.0 Hz, 1H), 4.51 (t, *J* = 4.0 Hz, 1H), 3.67 – 3.60 (m, 42H), 3.59 – 3.52 (m, 4H), 3.43 (t, *J* = 8.0 Hz, 2H), 3.37 (s, 3H), 3.24 (q, *J* = 8.0 Hz, 2H), 3.14 (q, *J* = 8.0, 4H), 2.23 (s, 3H), 1.61 - 1.42 (m, 8H), 1.37 - 1.35 (m, 4H), 1.33 – 1.23 (m, 12H). ^{13}C NMR (100 MHz, $CDCl_3$) δ 173.36, 158.59, 156.67, 154.88, 148.60, 106.73, 72.07, 71.65, 70.76, 70.74, 70.71, 70.66, 70.19, 59.18, 40.67, 40.28, 39.70, 30.49, 29.93, 29.75, 29.63, 29.57, 29.48, 29.41, 27.06, 26.35, 26.28, 26.20, 19.10. LC-MS: calc.: *m/z* = 964.63, found = 965.67 $[M+H]^+$, 484.33 $[M+2H]^{2+}$.

1-(6-methyl-4-oxo-1,4-dihydropyrimidin-2-yl)-3-(45-oxo-2,5,8,11,14,17,20,23,26,29,32,35-dodecaoxa-44,46-diazadopentacontan-52-yl)urea (C12-O)

The synthesis of **C12-O** was performed following the general procedure 3, providing **C12-O** (446 mg; 0.45 mmol) as a white solid in quantitative yield. ^{1}H-NMR (400 MHz, $CDCl_3$) δ 13.16 (s, 1H), 11.82 (s, 1H), 10.07 (s, 1H), 5.82 (s, 1H), 4.77 (t, J = 4.0 Hz, 1H), 4.56 (t, J = 4.0 Hz, 1H), 3.64 (m, 42H), 3.58-3.53 (m, 4H), 3.43 (t, J = 8.0 Hz, 2H), 3.37 (s, 3H), 3.23 (td, $J_{HC\text{-}CH}$ = 8.0, Hz $J_{HC\text{-}NH}$ = 4.0 Hz, 2H), 3.14 (td, $J_{\text{HC-CH}}$ = 8.0 Hz, $J_{HC\text{-}NH}$ = 4.0 Hz, 4H), 2.23 (s, 3H), 1.63 – 1.41 (m, 8H), 1.38 – 1.19 (m, 18H). ^{13}C NMR (100 MHz, $CDCl_3$) δ 173.37, 158.63, 156.66, 154.88, 148.61, 106.70, 72.06, 71.67, 70.75, 70.70, 70.64, 70.18, 59.17, 40.65, 40.26, 39.70, 30.50, 29.92, 29.76, 29.71, 29.61, 29.51, 29.41, 27.08, 26.35, 26.27, 26.21, 19.09. LC-MS: calc. *m/z*: 992.66; found *m/z*: $[M+H]^+$ = 993.75 and $[M+H+NH_4]^{2+}$ = 506.00.

2 Molecular characterization

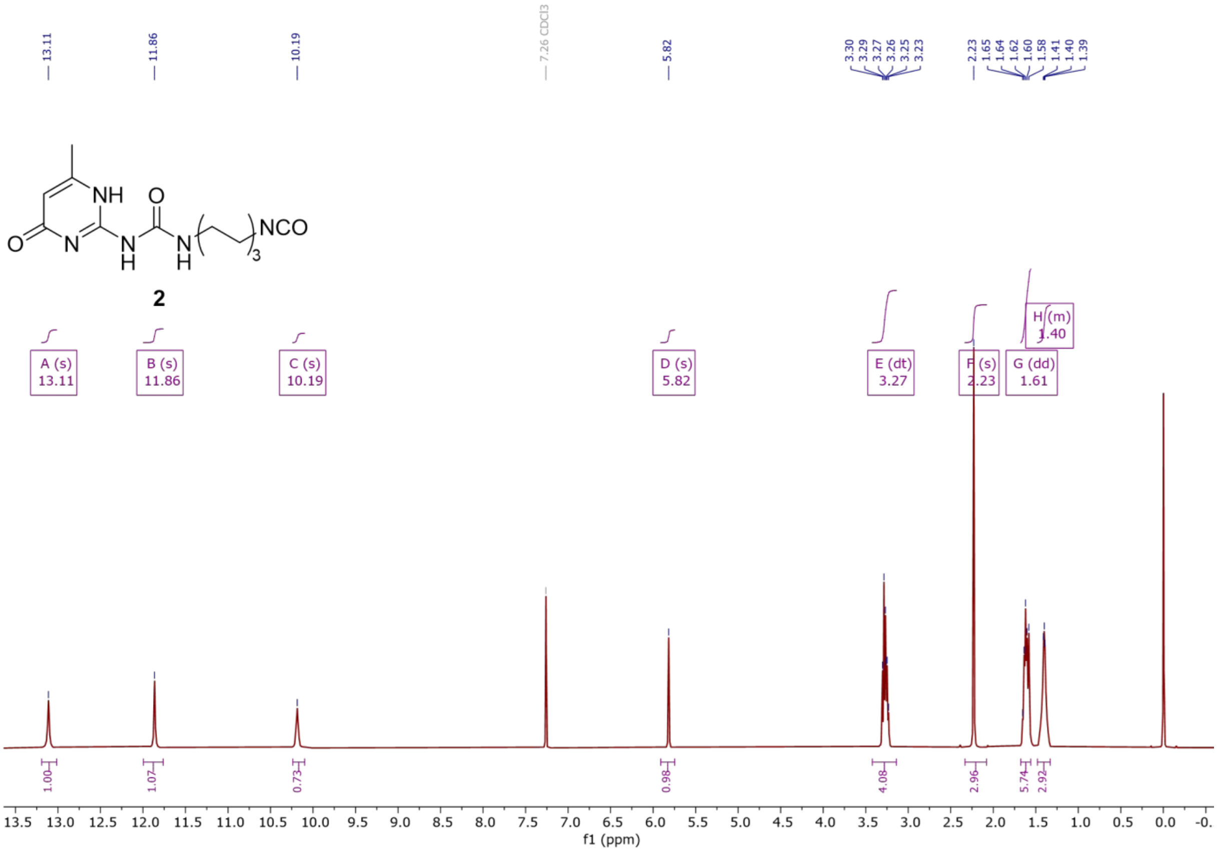

Figure S1. ^{1}H-NMR of compound **2** in $CDCl_3$.

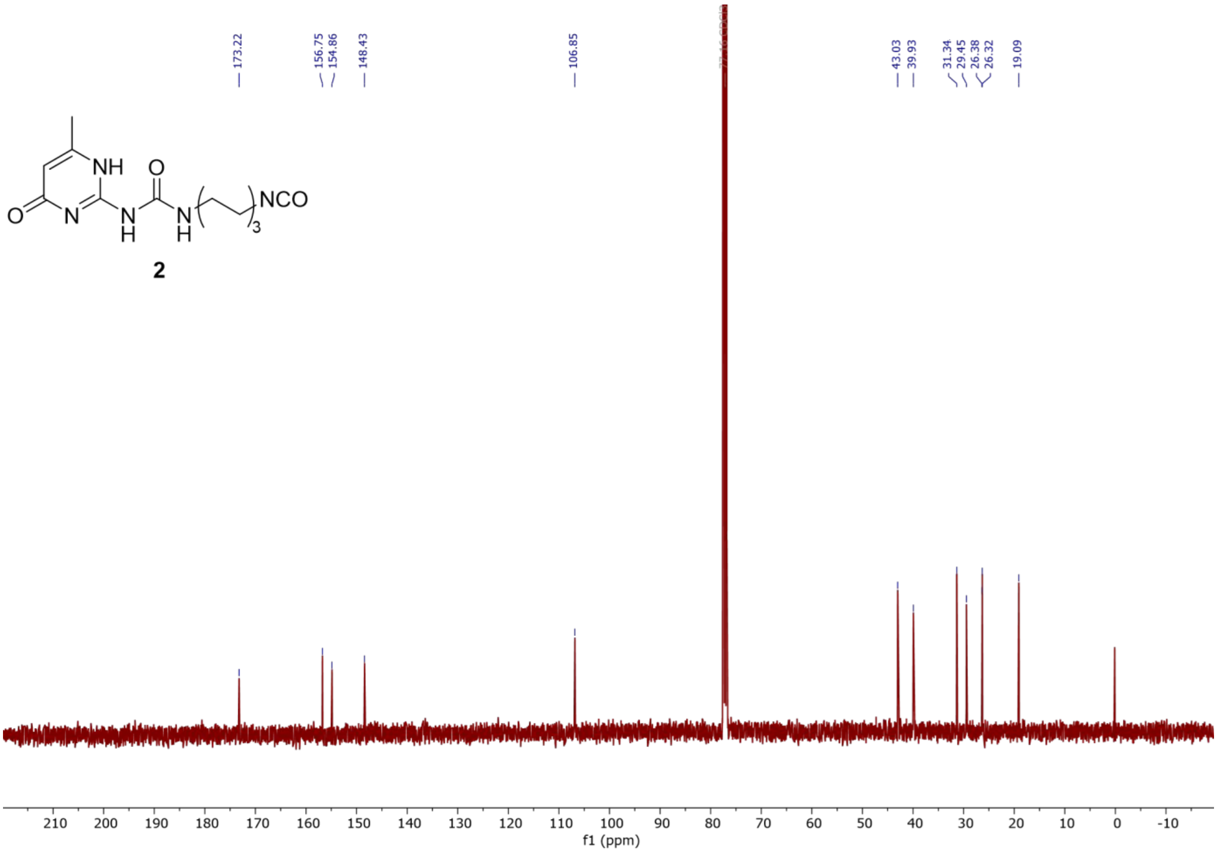

Figure S2. ^{13}C-NMR of compound **2** in $CDCl_3$.

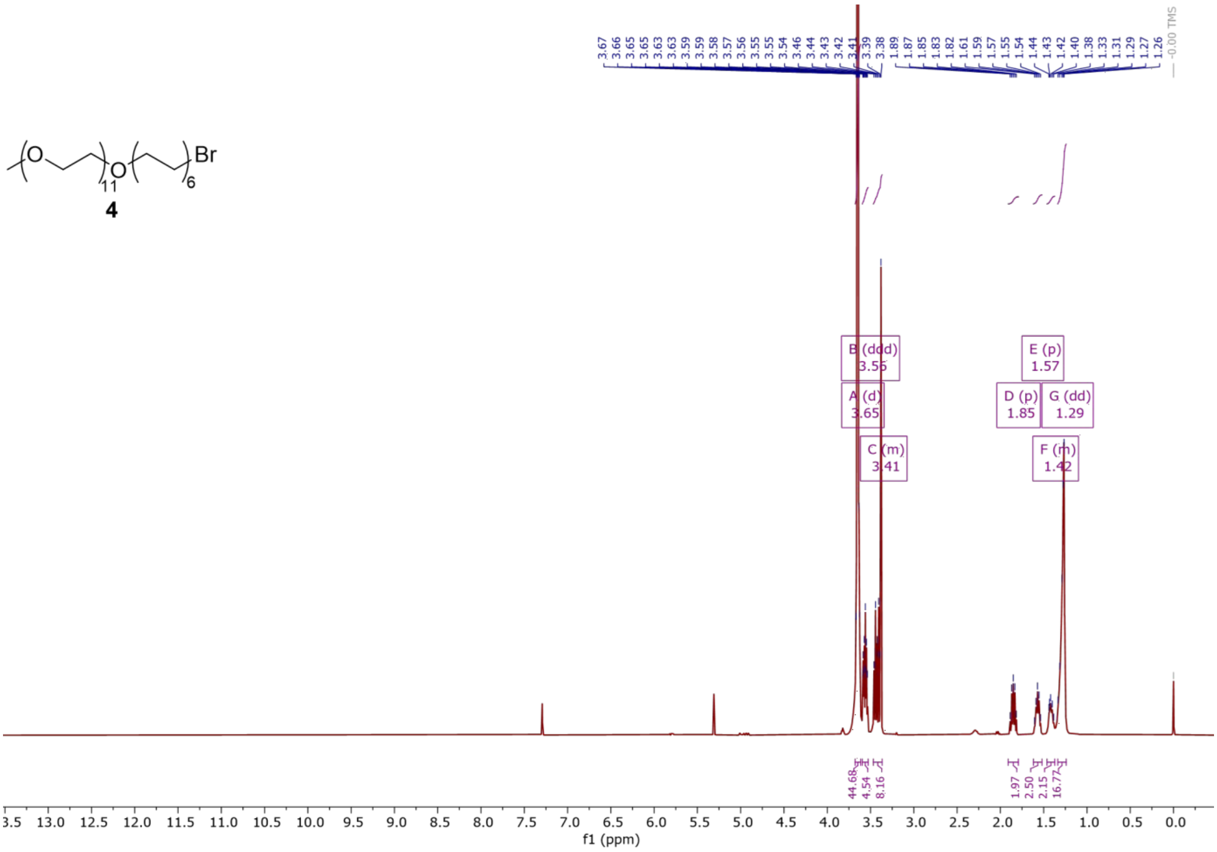

Figure S3. ^{1}H-NMR of **4** in $CDCl_3$.

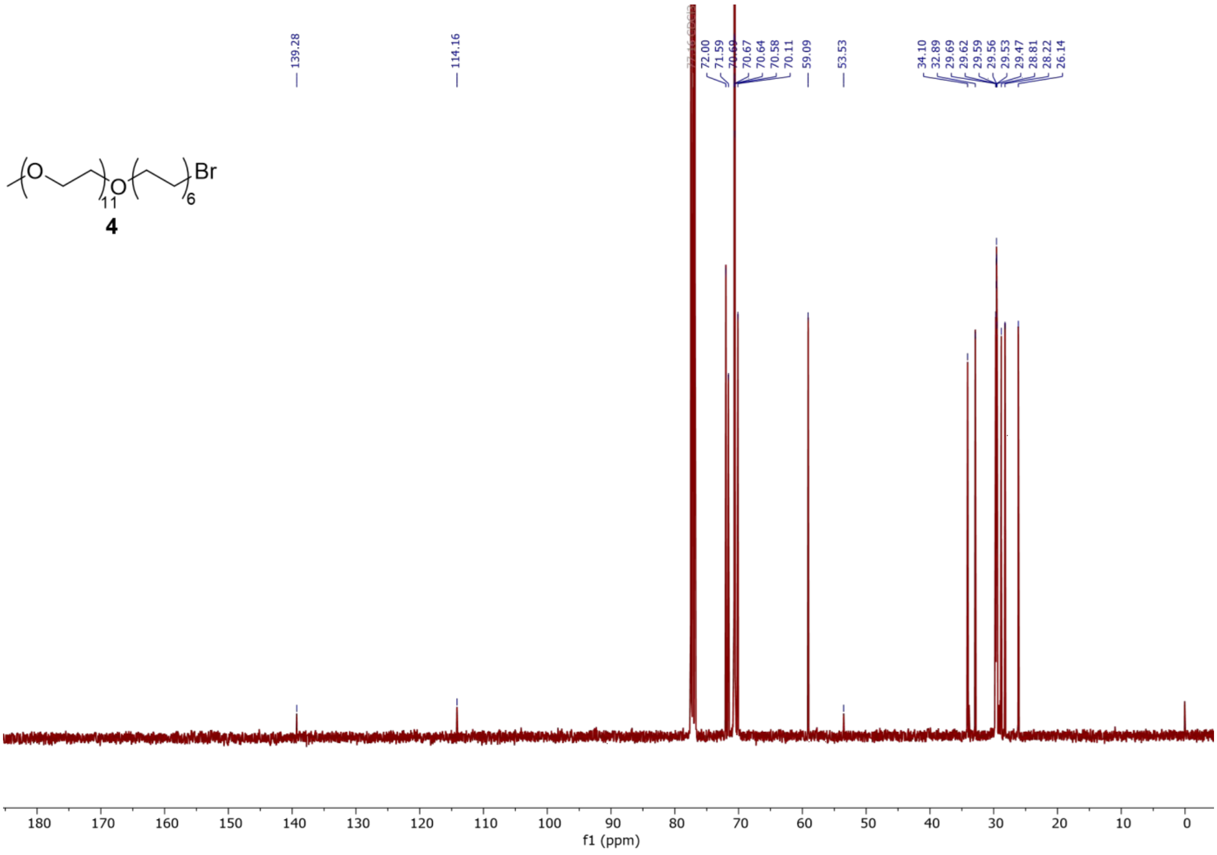

Figure S4. ^{13}C-NMR of **4** in $CDCl_3$.

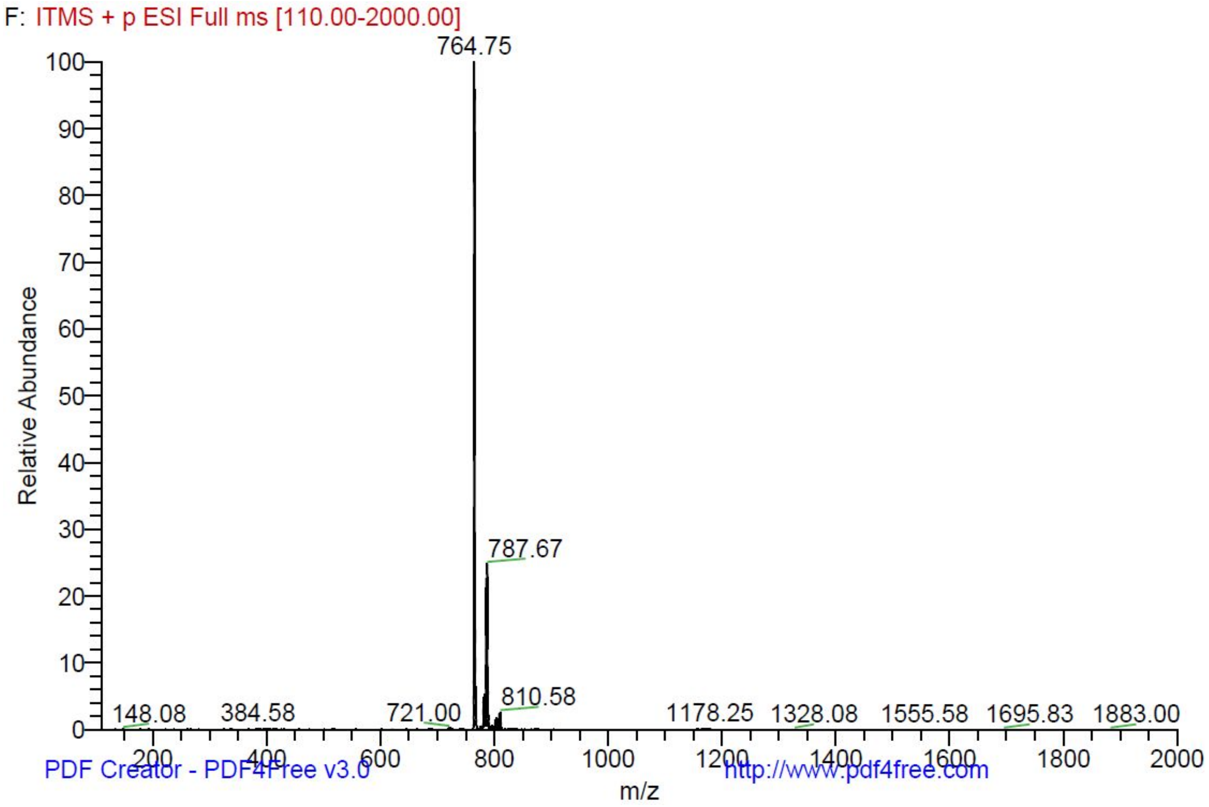

Figure S5. ESI-MS (ESI+, H_2O:CH_3CN = 1:1) spectrum of compound **4**.

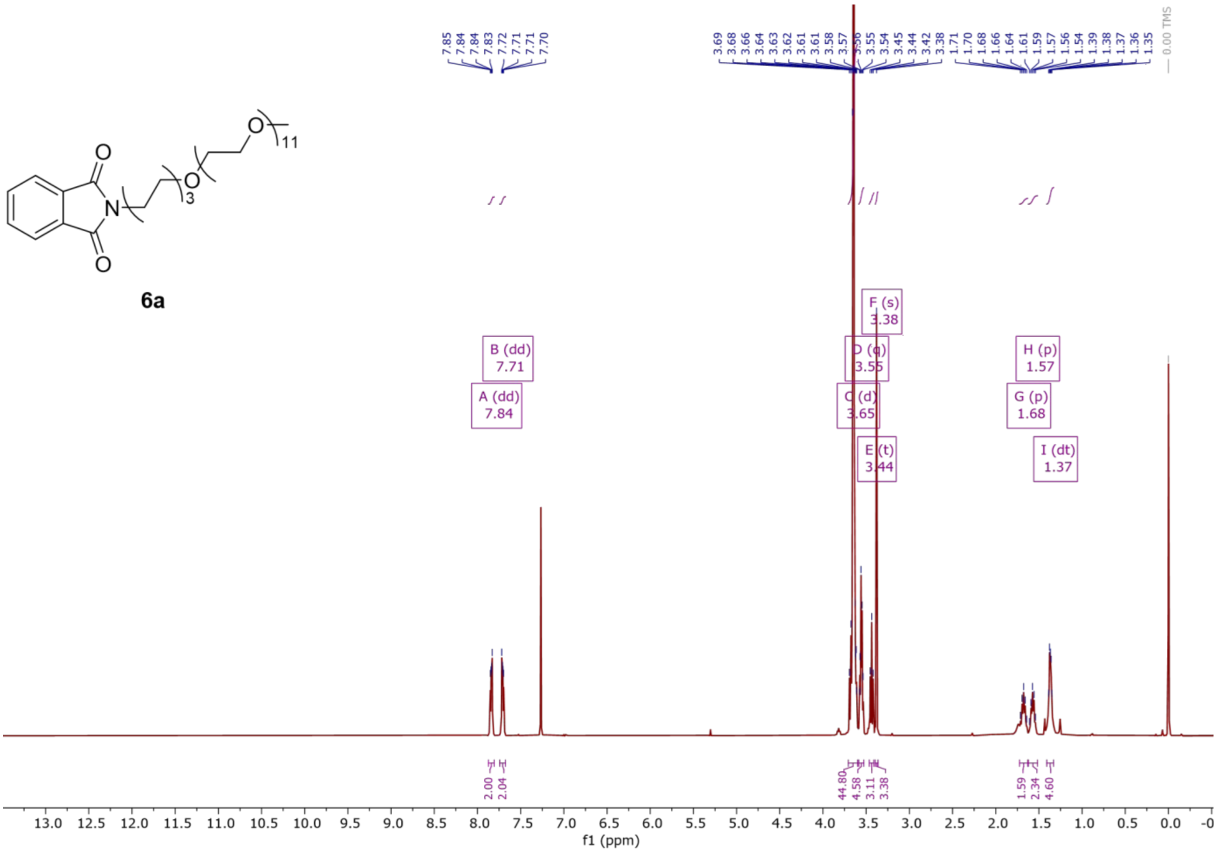

Figure S6. ^{1}H-NMR of compound **6a** in $CDCl_3$.

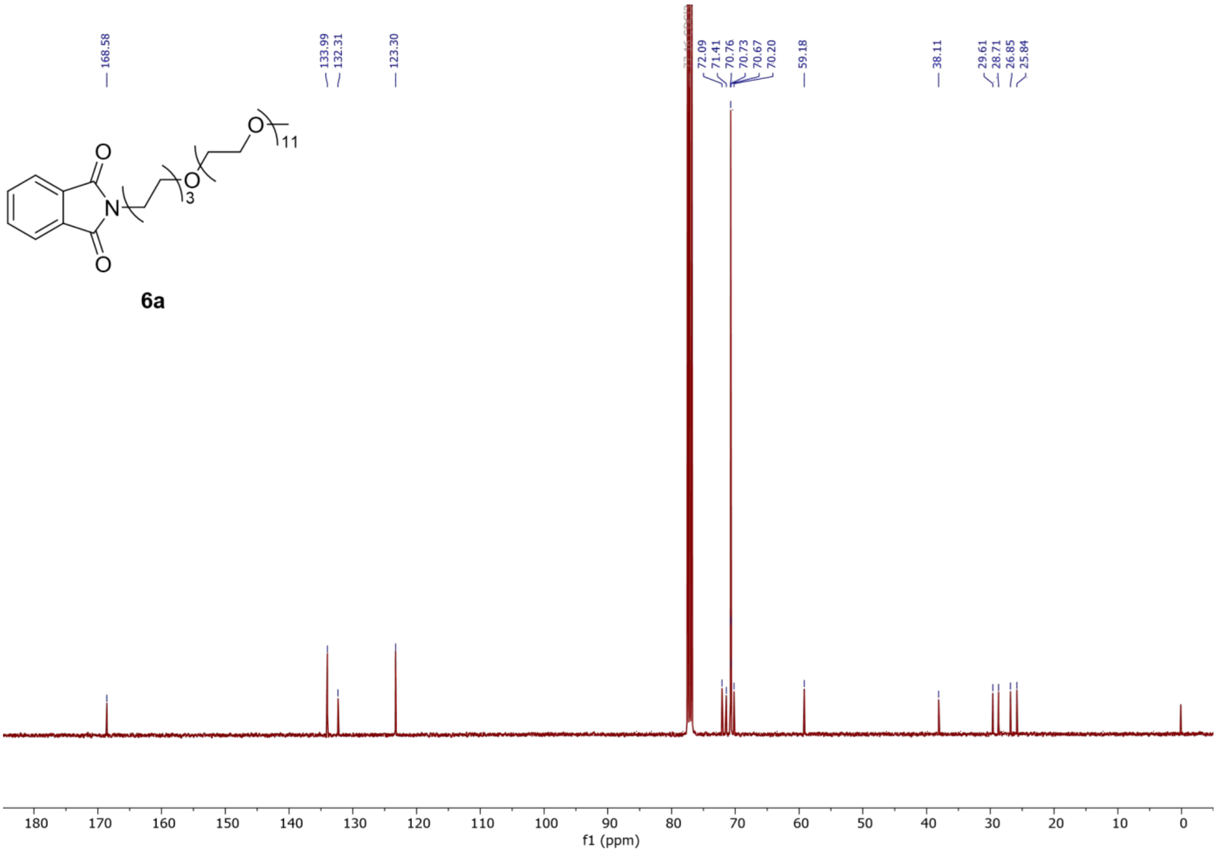

Figure S7. ^{13}C-NMR of compound **6a** in $CDCl_3$.

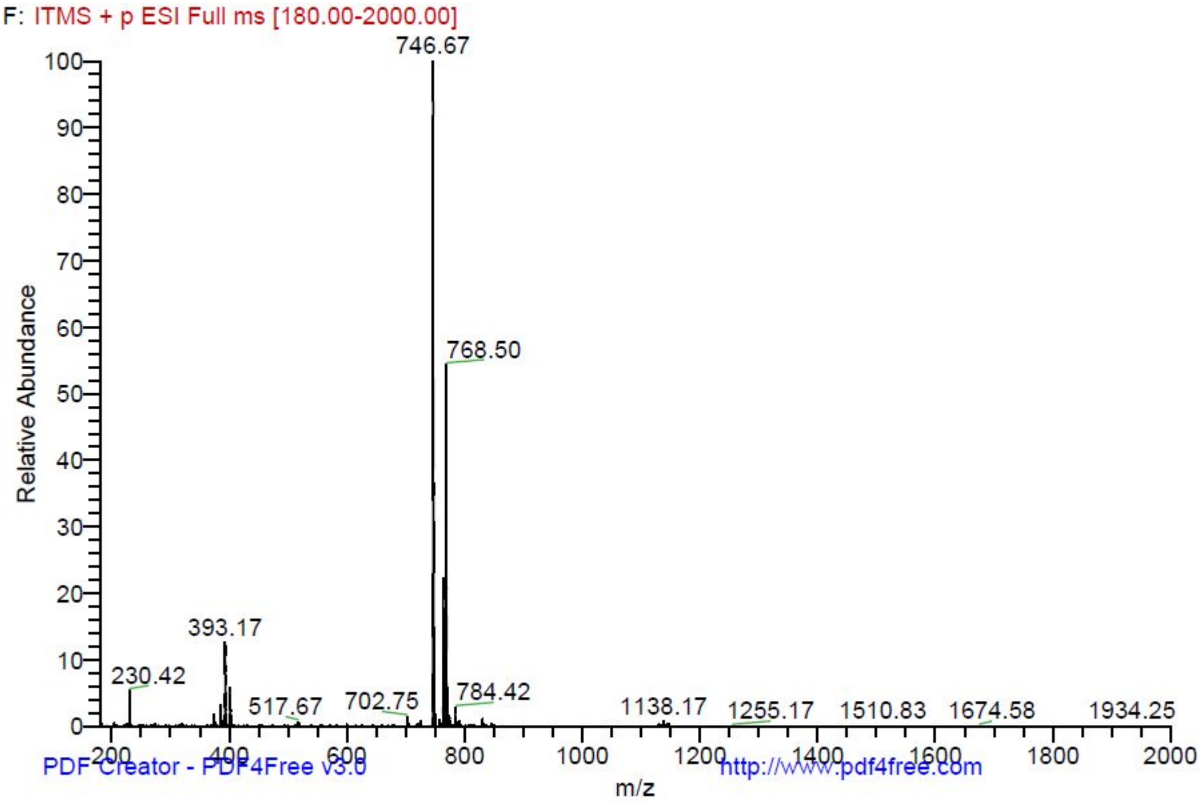

Figure S8. ESI-MS (ESI+, H_2O:CH_3CN = 1:1) spectrum of compound **6a.**

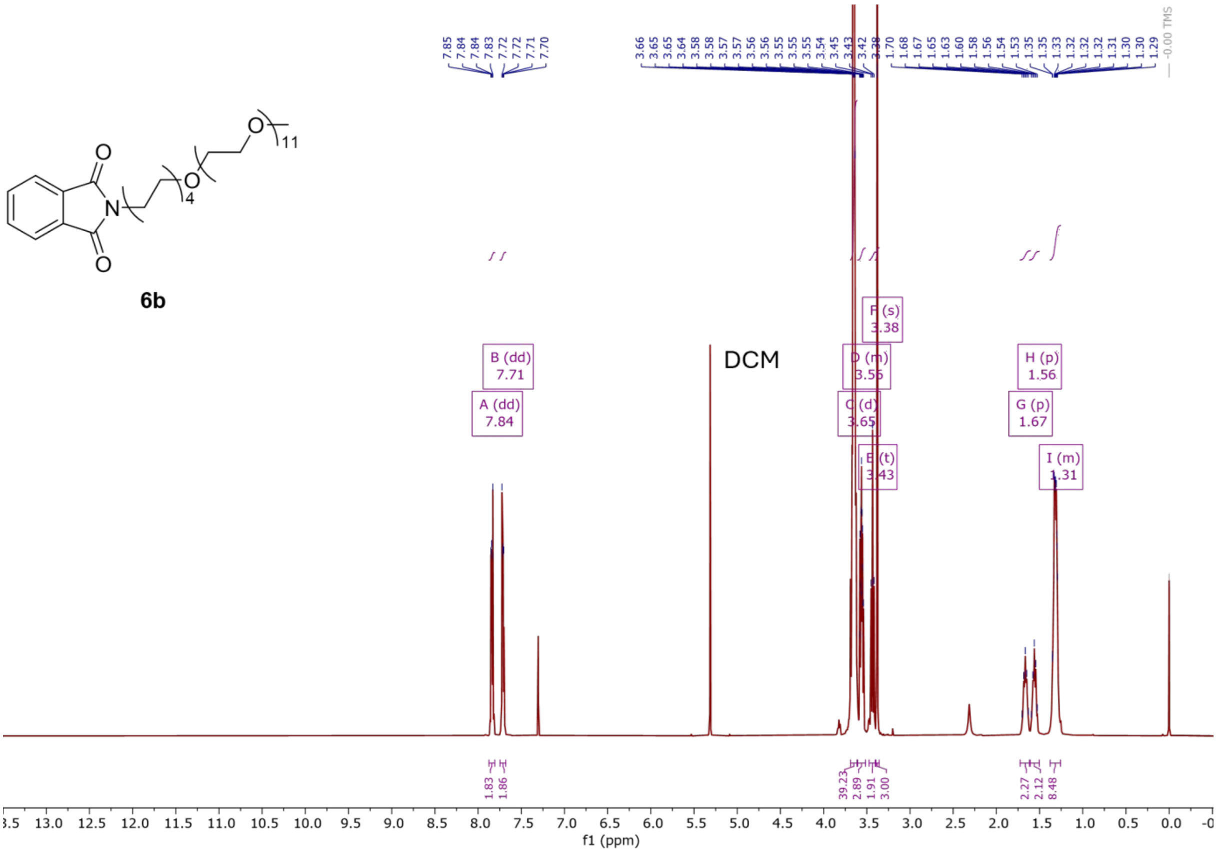

Figure S9. ^{1}H-NMR of compound **6b** in $CDCl_3$.

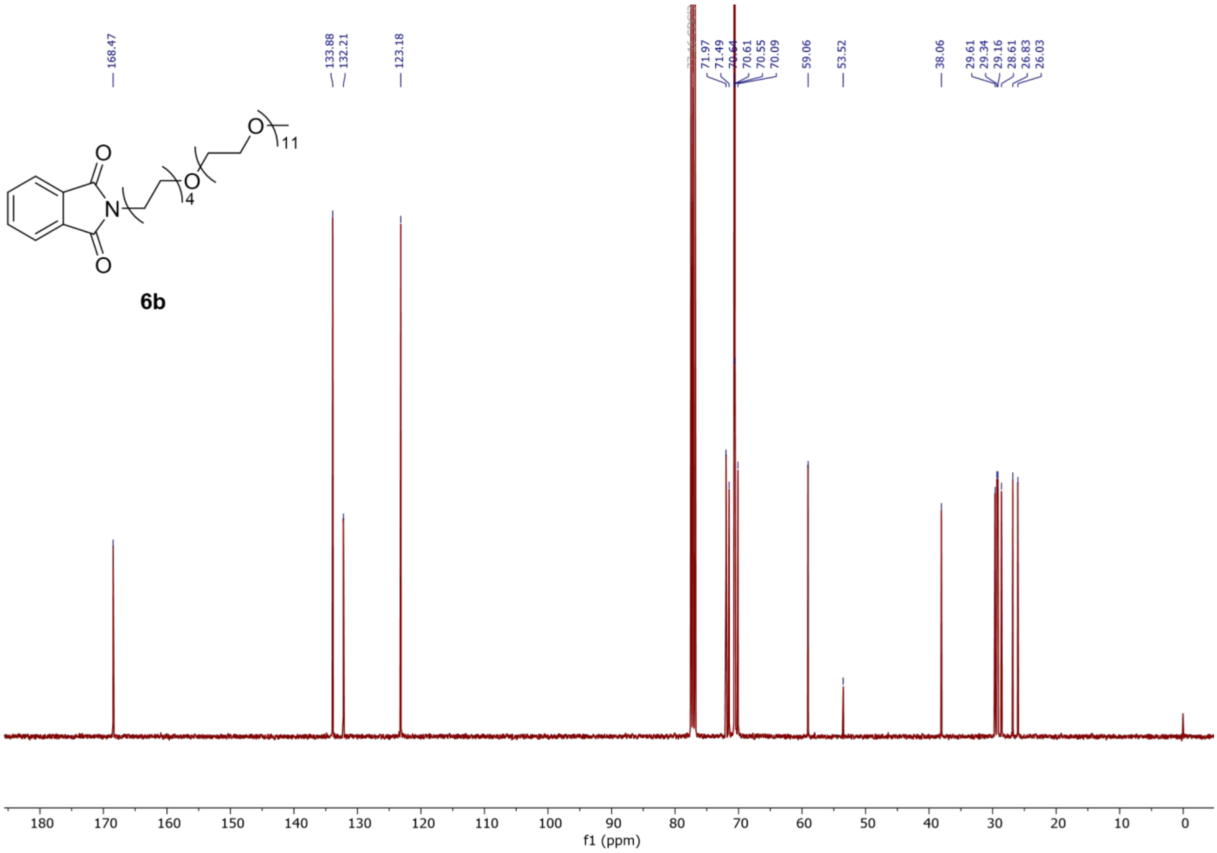

Figure S10. ^{13}C-NMR of compound **6b** in $CDCl_3$.

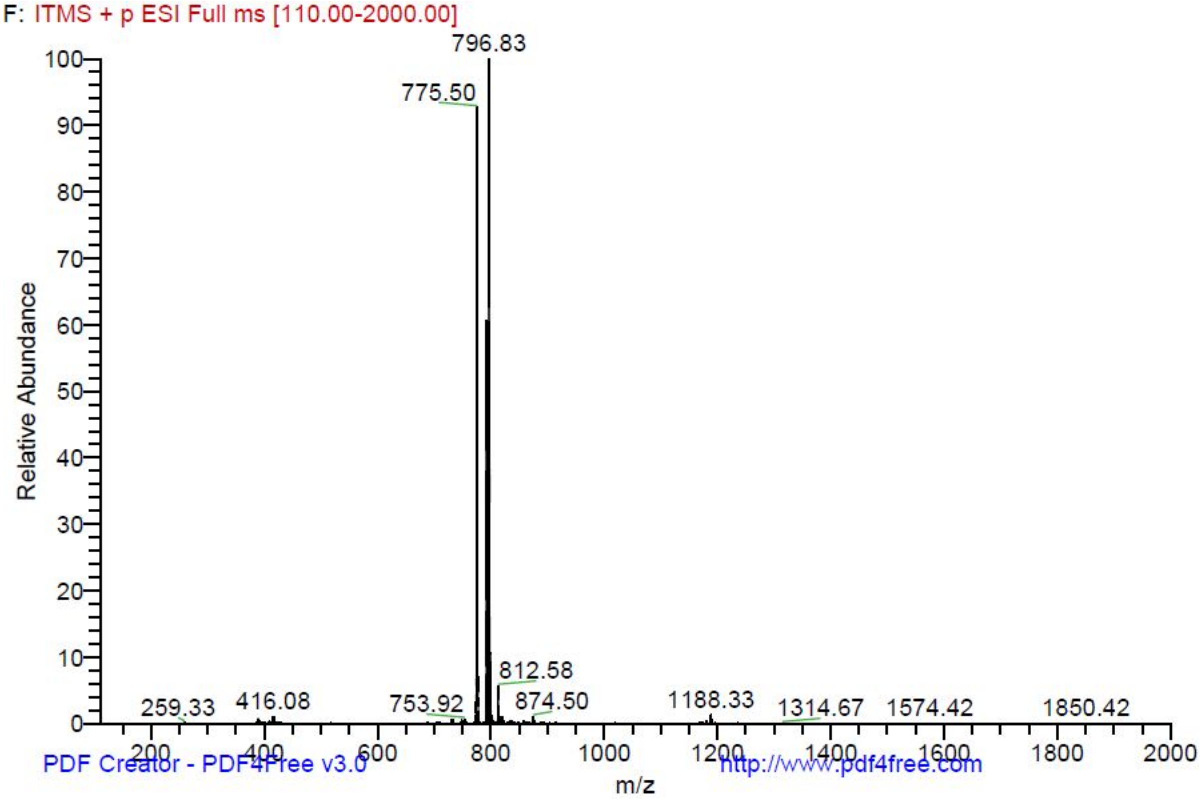

Figure S11. ESI-MS (ESI+, H_2O:CH_3CN = 1:1) spectrum of compound **6b.**

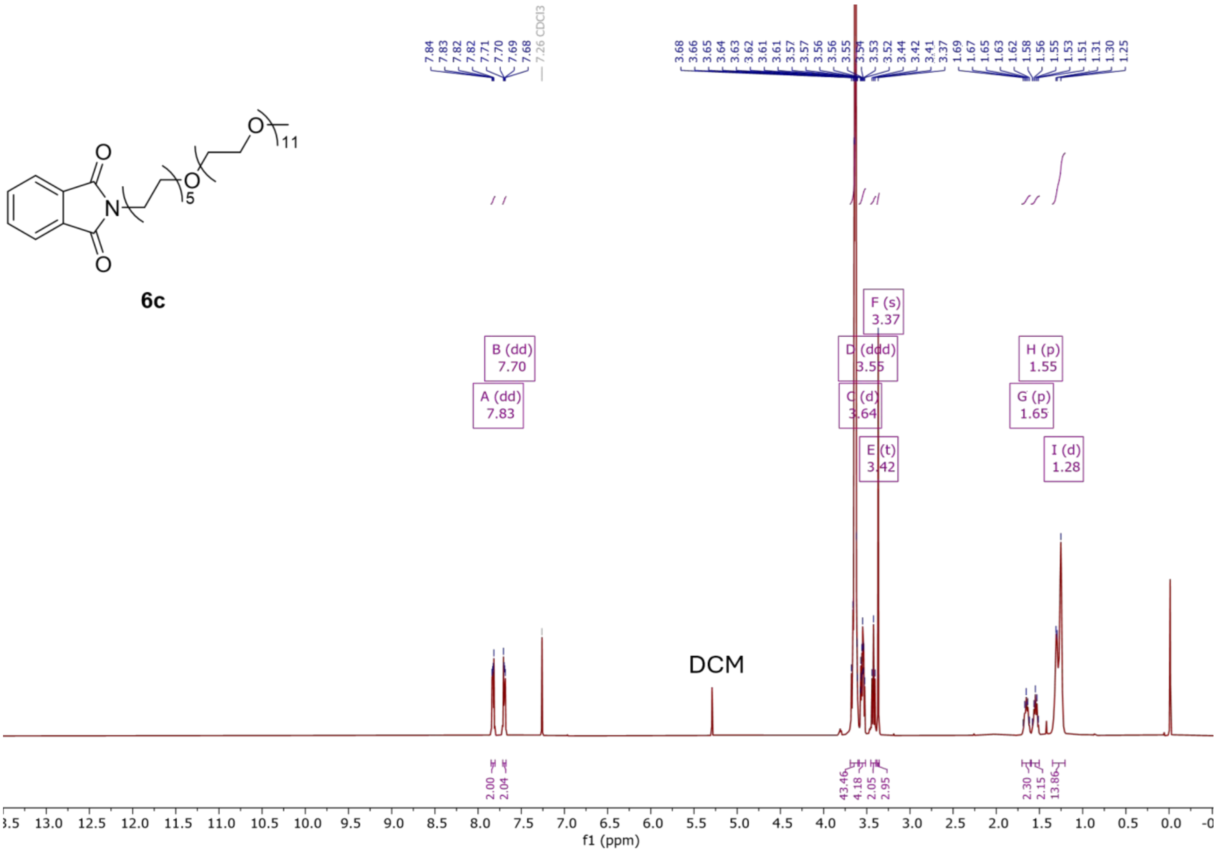

Figure S12. ^{1}H-NMR of compound **6c** in $CDCl_3$.

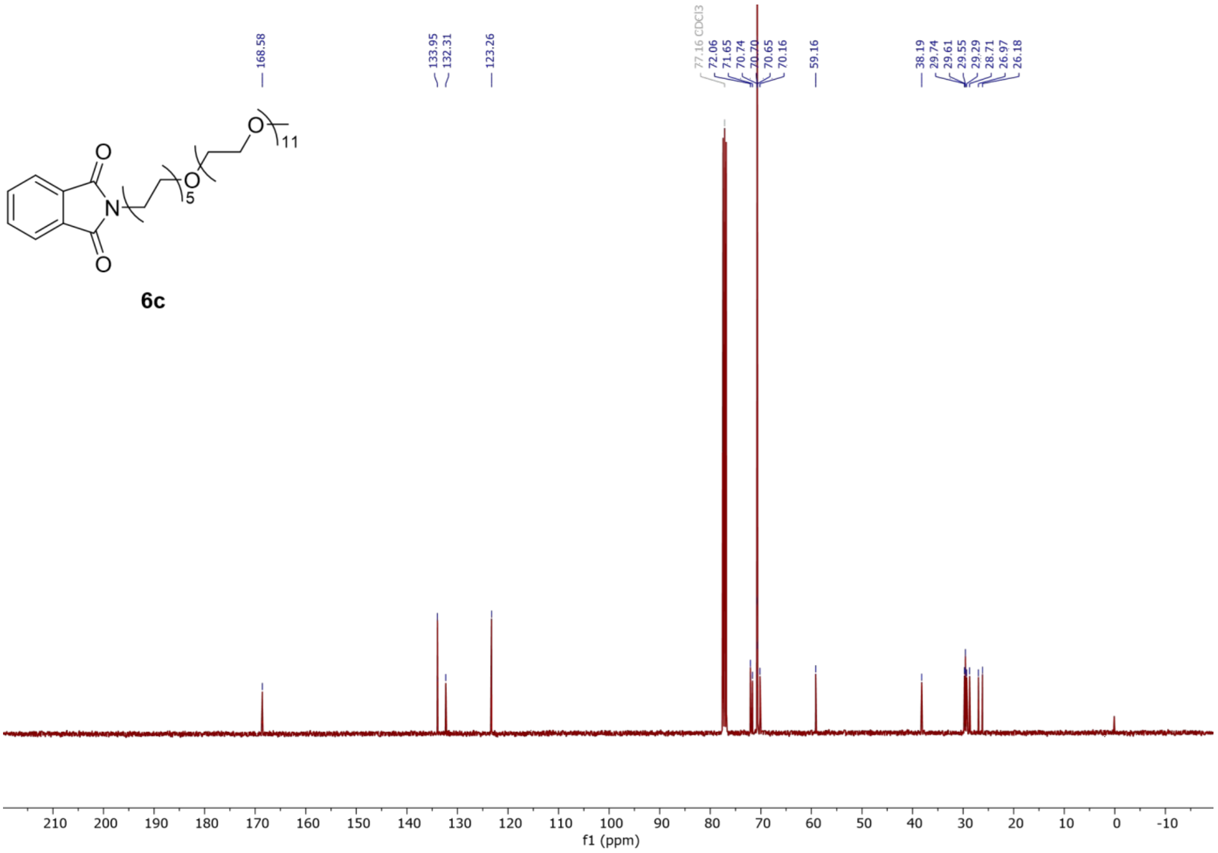

Figure S13. ^{13}C-NMR of compound **6c** in $CDCl_3$.

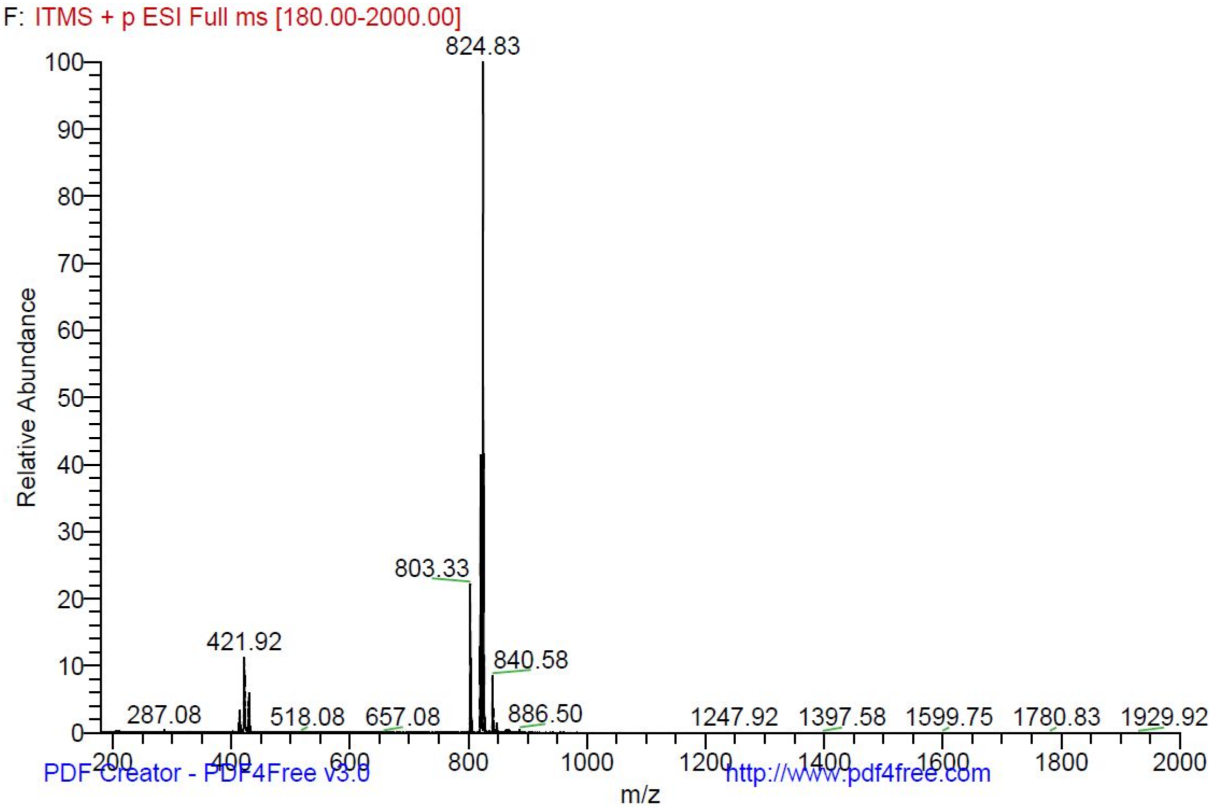

Figure S14. ESI-MS (ESI+, $H_2O:CH_3CN$ = 1:1) spectrum of compound **6c**.

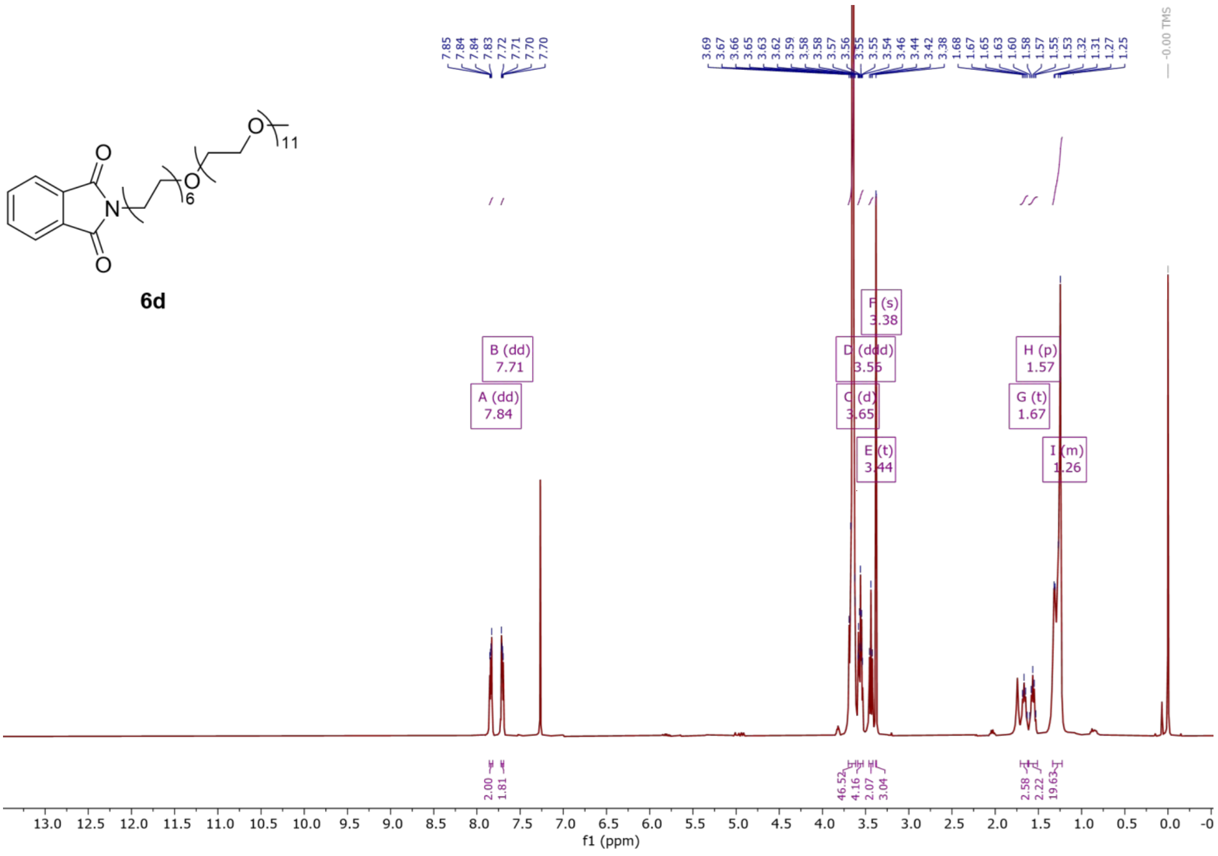

Figure S15. ^1H-NMR of compound **6d** in $CDCl_3$.

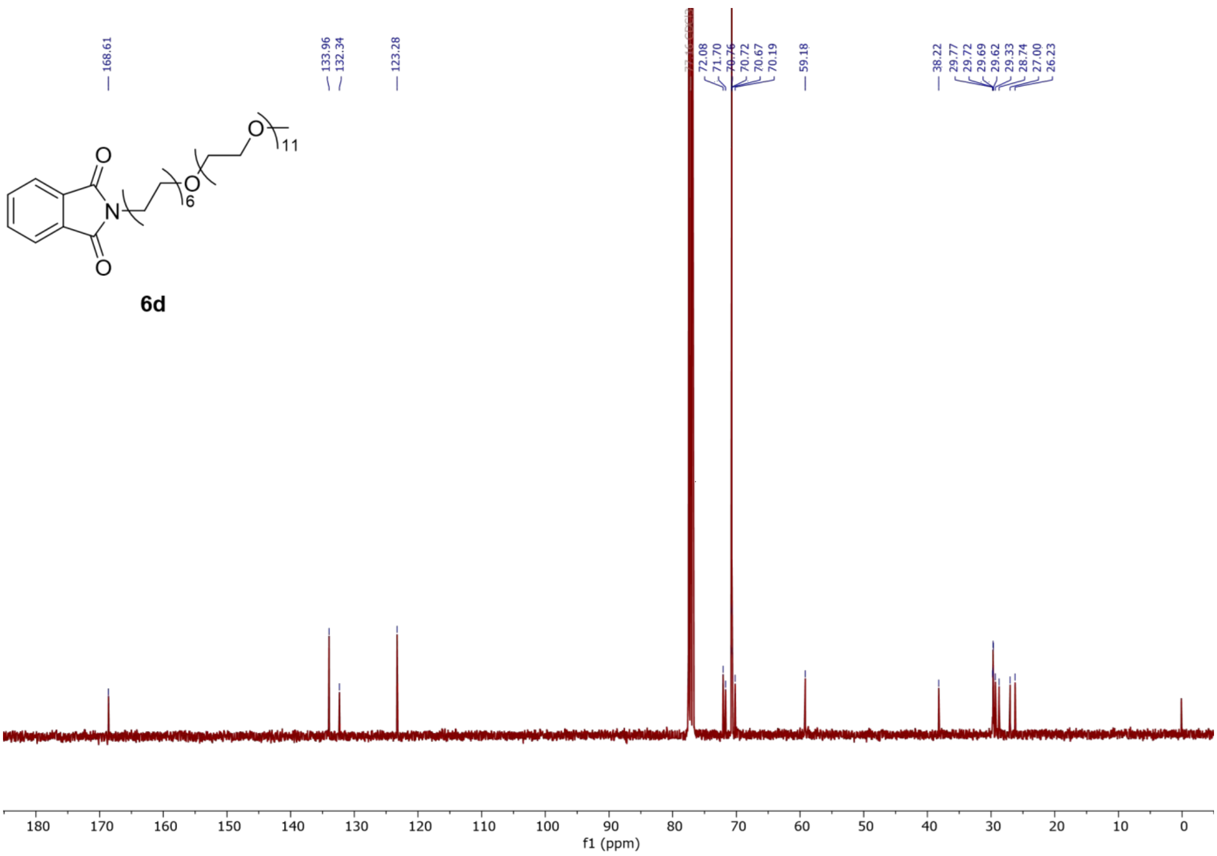

Figure S16. ^{13}C-NMR of compound **6d** in $CDCl_3$.

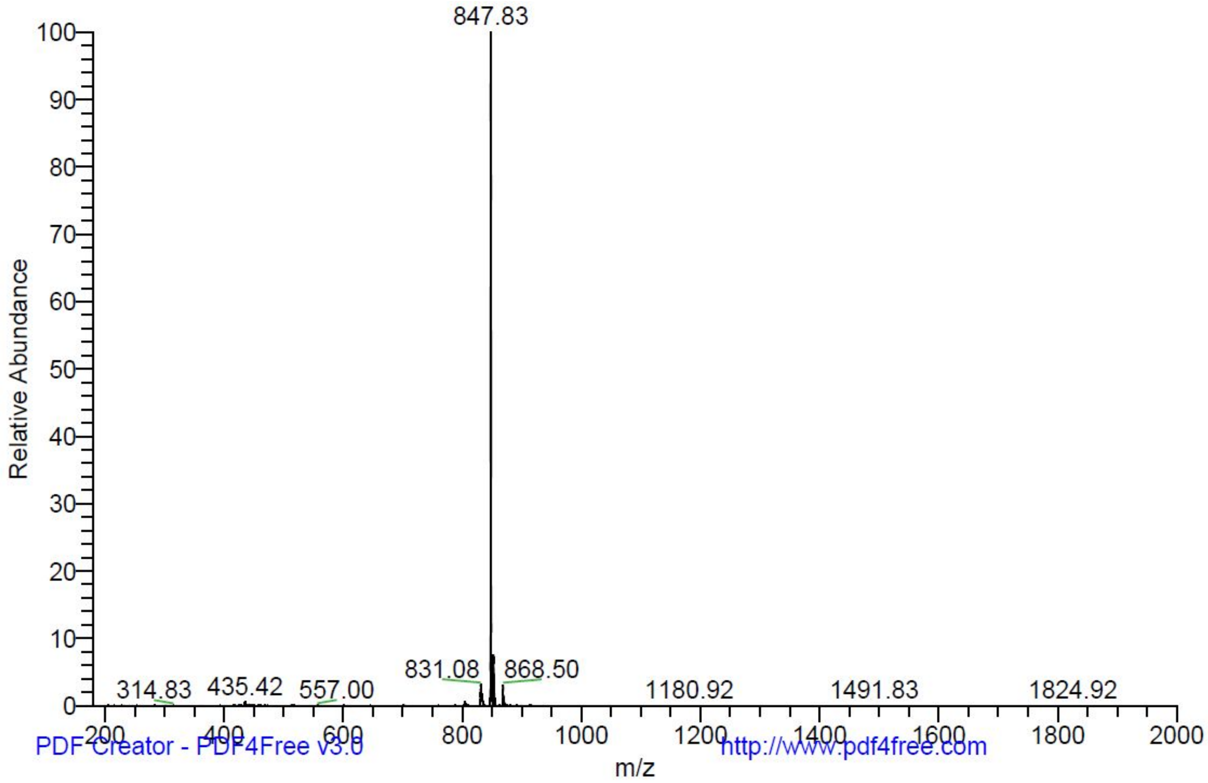

Figure S17. ESI-MS (ESI+, H_2O:CH_3CN = 1:1) spectrum of compound **6d**.

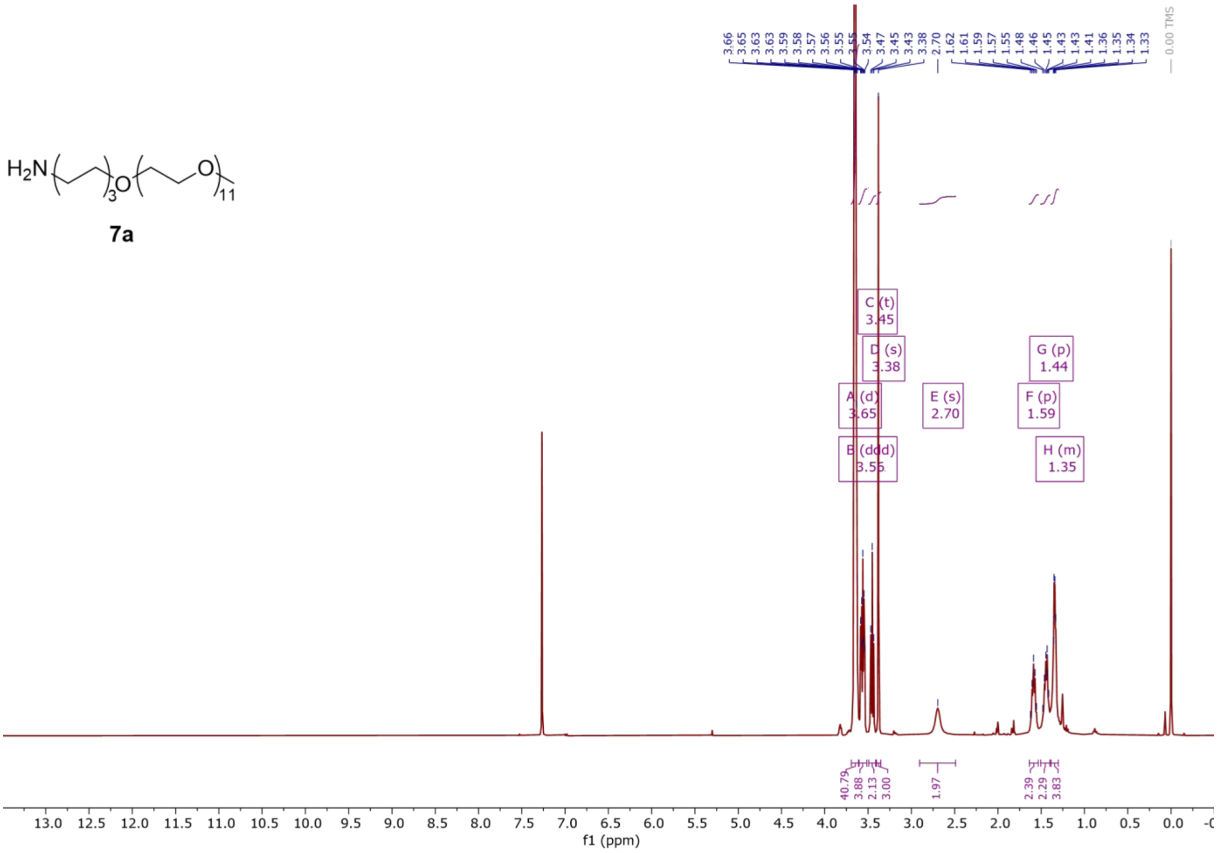

Figure S18. ^{1}H-NMR of compound **7a** in $CDCl_3$.

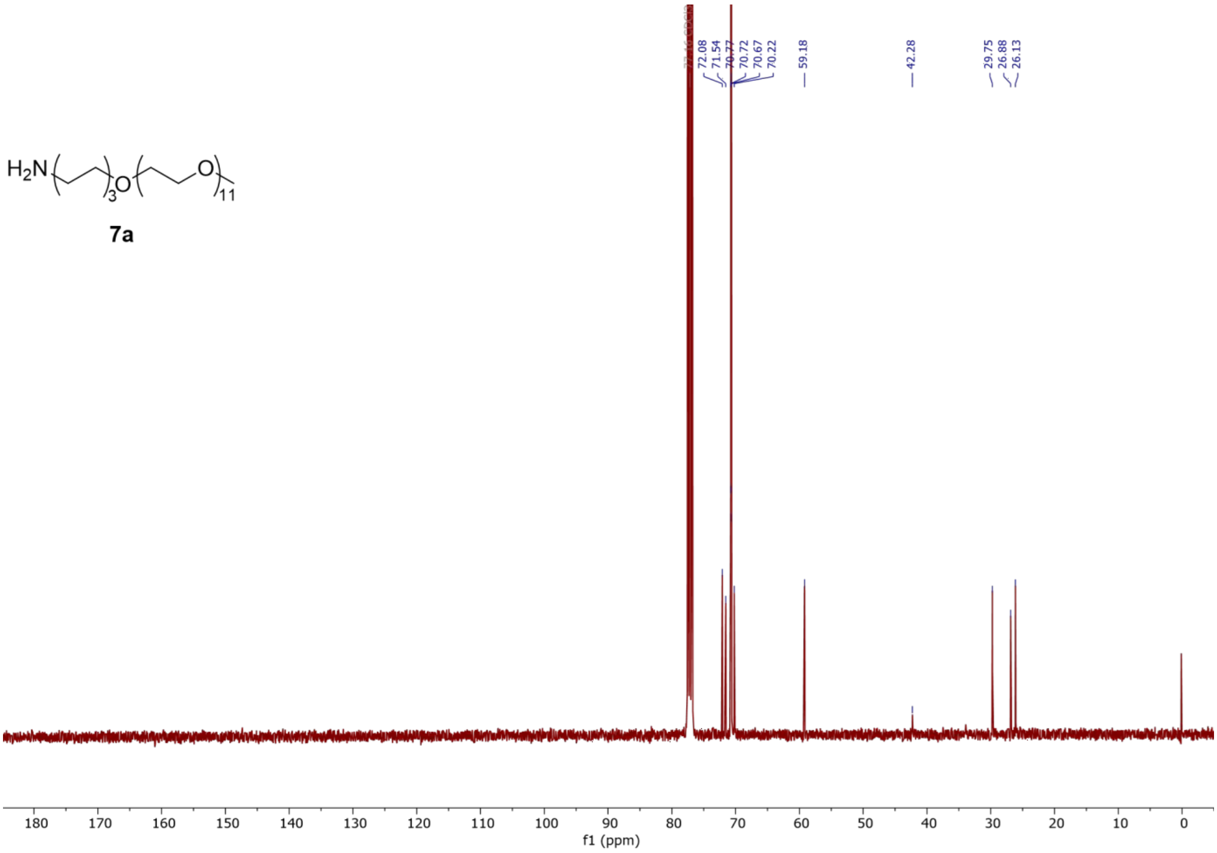

Figure S19. ^{13}C-NMR of compound **7a** in $CDCl_3$.

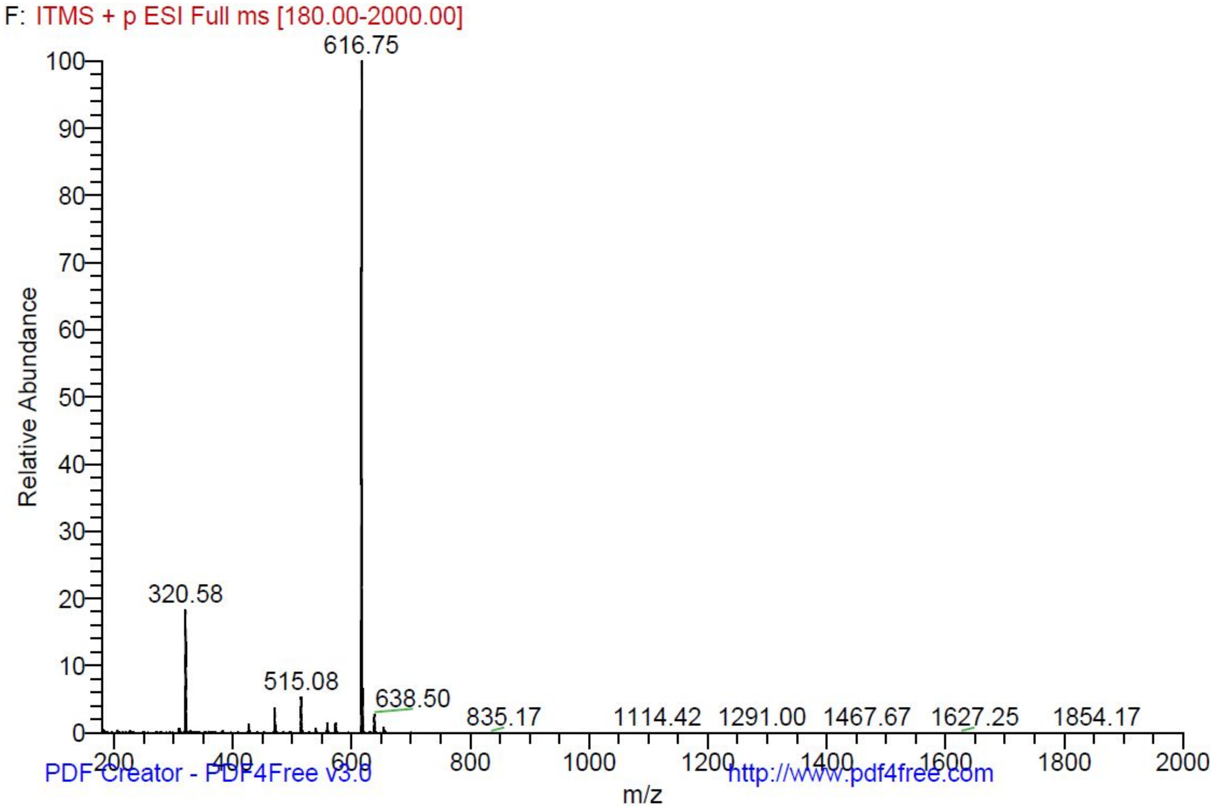

Figure S 20. ESI-MS (ESI+, H_2O:CH_3CN = 1:1) spectrum of compound **7a**.

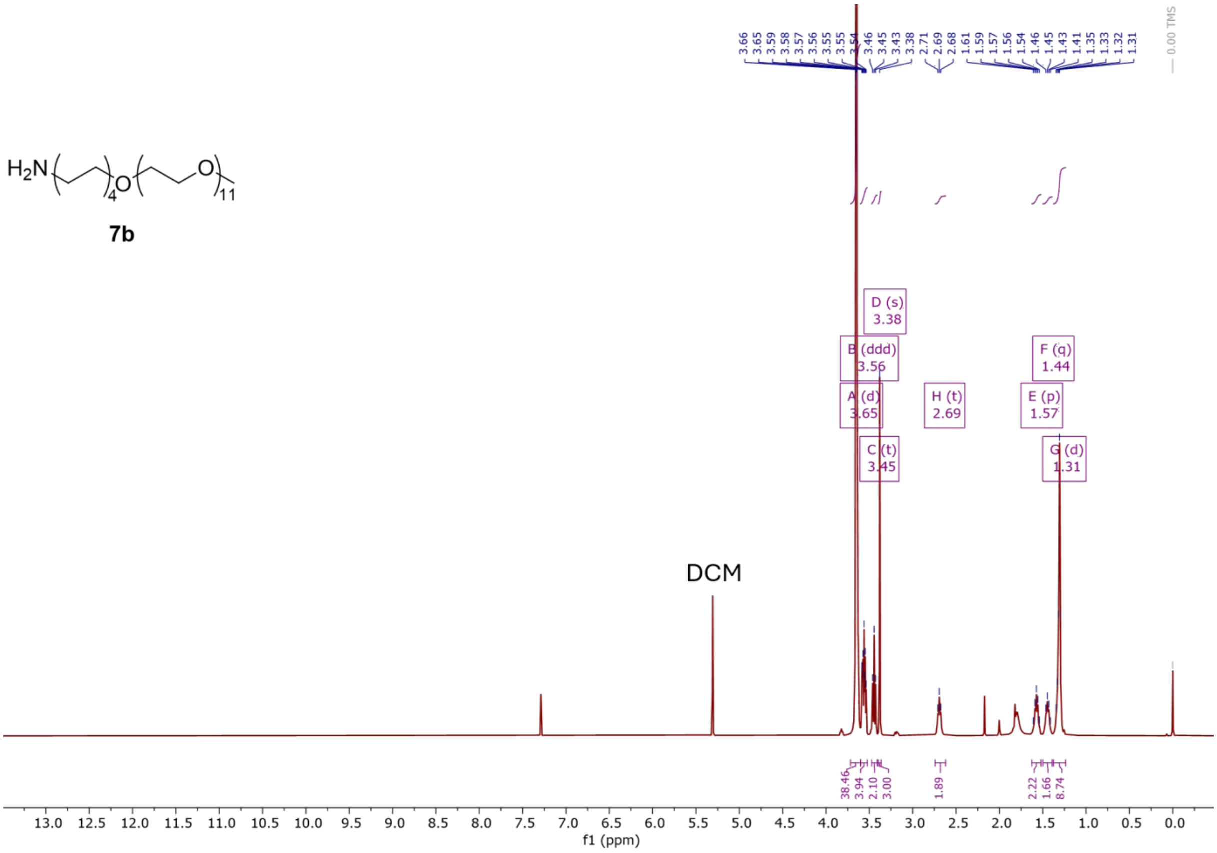

7b

Figure S21. ^{1}H-NMR of compound **7b** in $CDCl_3$.

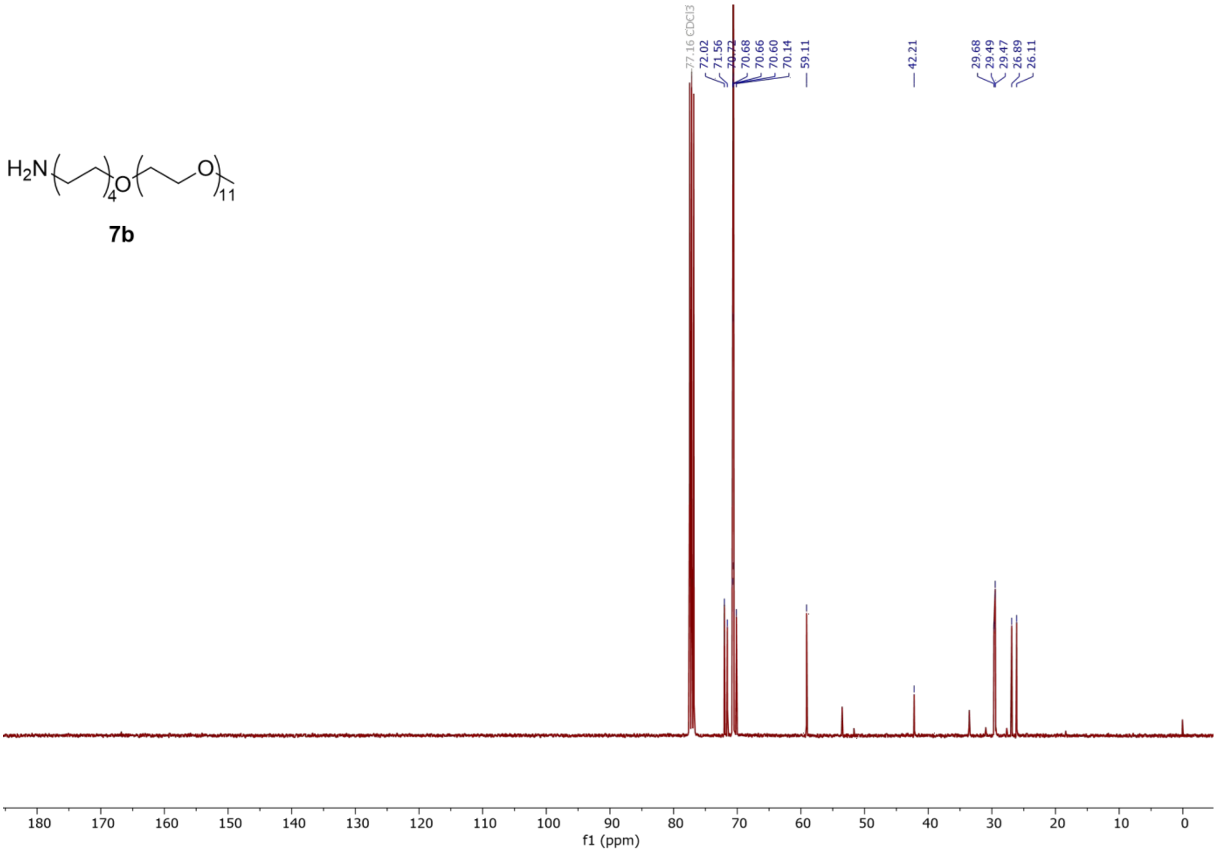

Figure S22. ^{13}C-NMR of compound **7b** in $CDCl_3$.

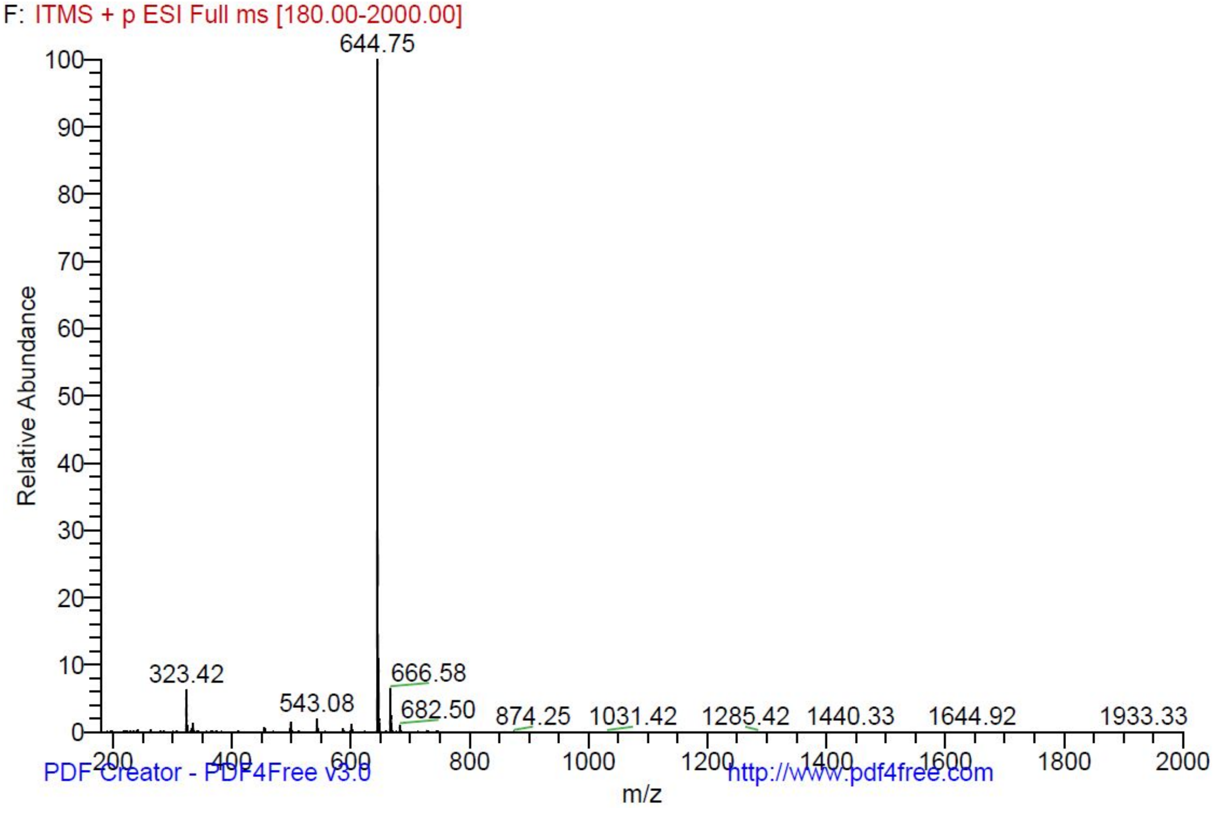

PDF Creator - PDF4Free v3.0 http://www.pdf4free.com

Figure S23. ESI-MS spectrum (ESI+, $H_2O:CH_3CN$ = 1:1) of compound **7b**.

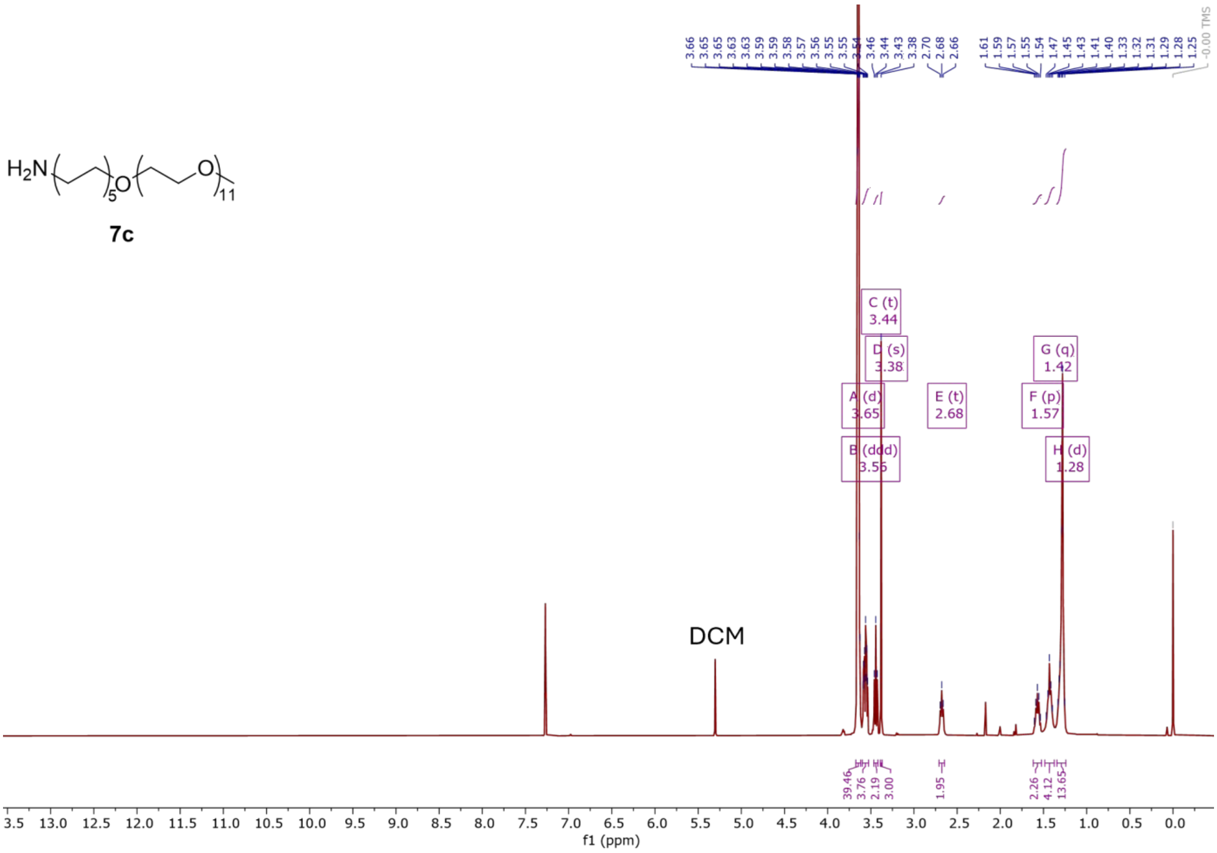

Figure S24. ^{1}H-NMR of compound **7c** in $CDCl_3$.

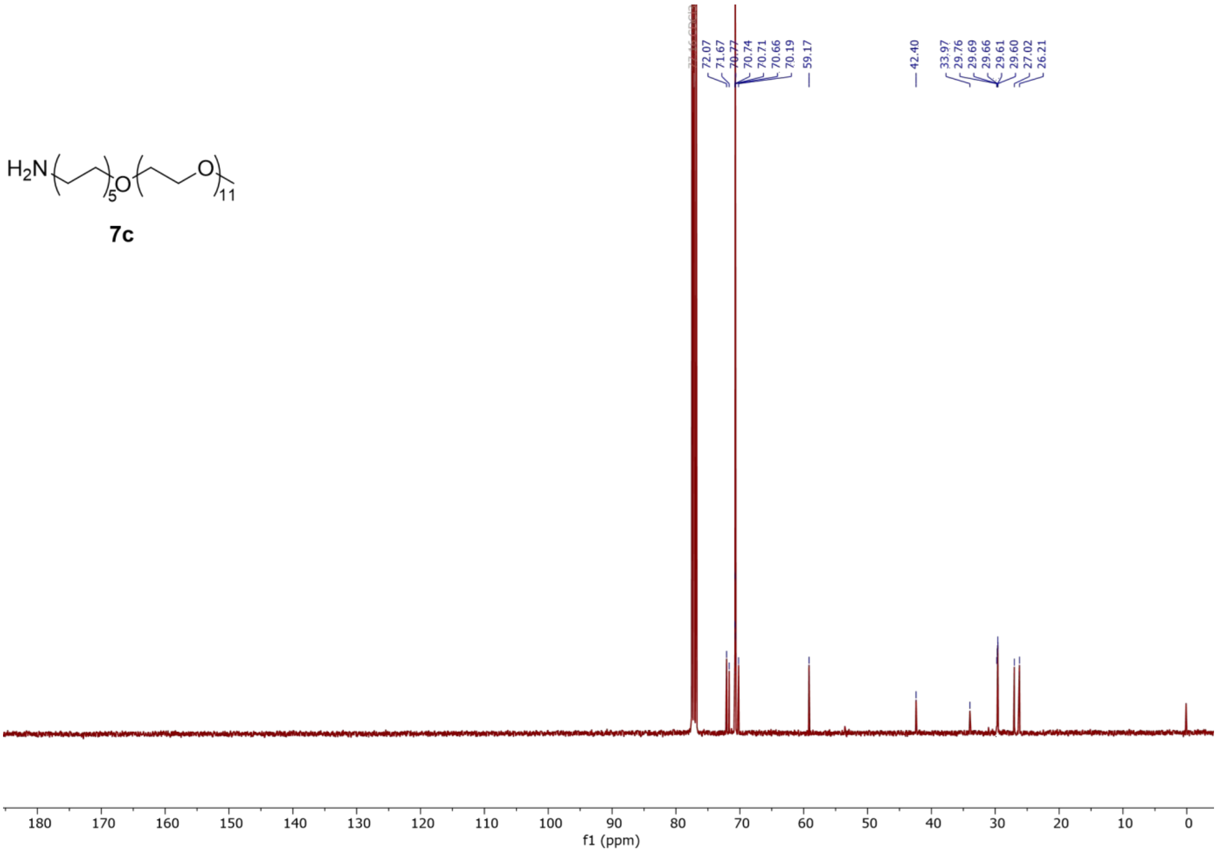

Figure S25. ^{13}C-NMR of compound **7c** in $CDCl_3$.

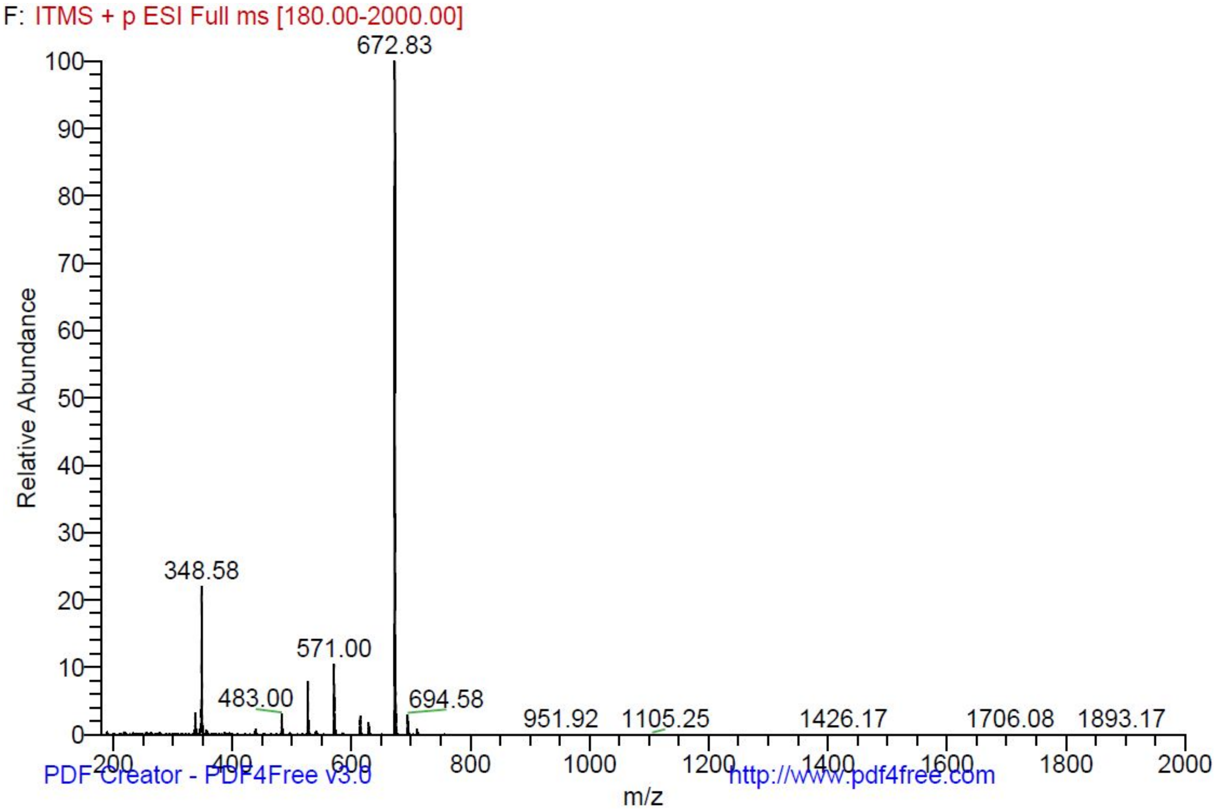

Figure S26. ESI-MS spectrum (ESI+, H_2O:CH_3CN = 1:1) of compound **7c**.

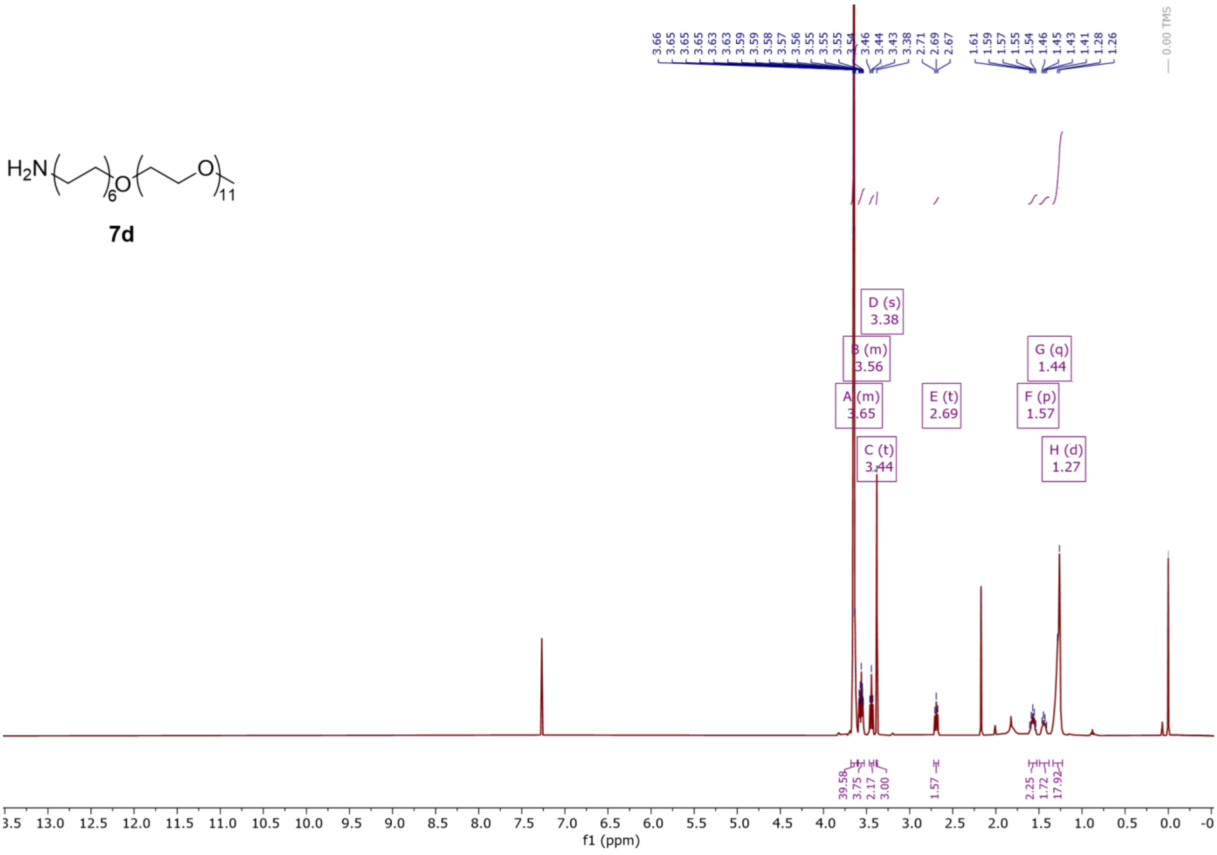

Figure S27. ^{1}H-NMR of compound **7d** in $CDCl_3$.

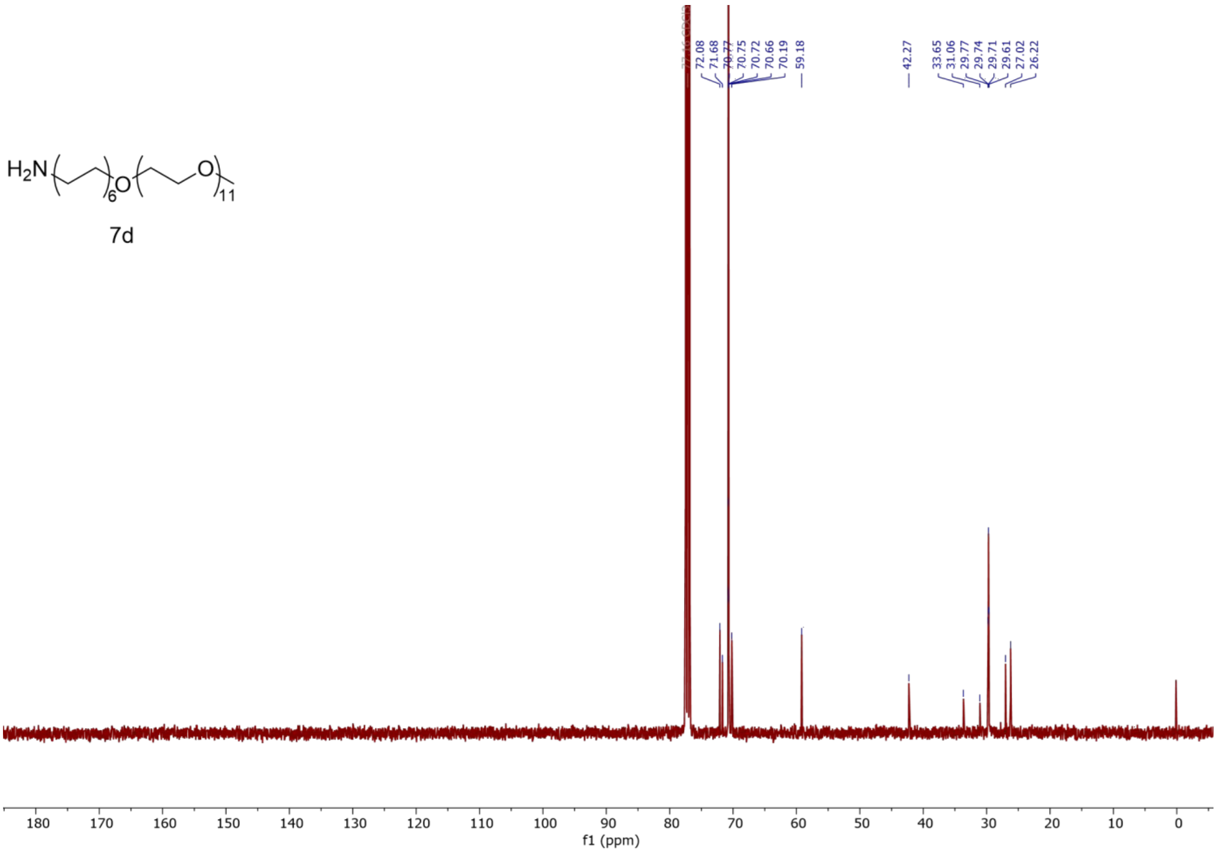

Figure S28. ^{13}C-NMR of compound **7d** in $CDCl_3$.

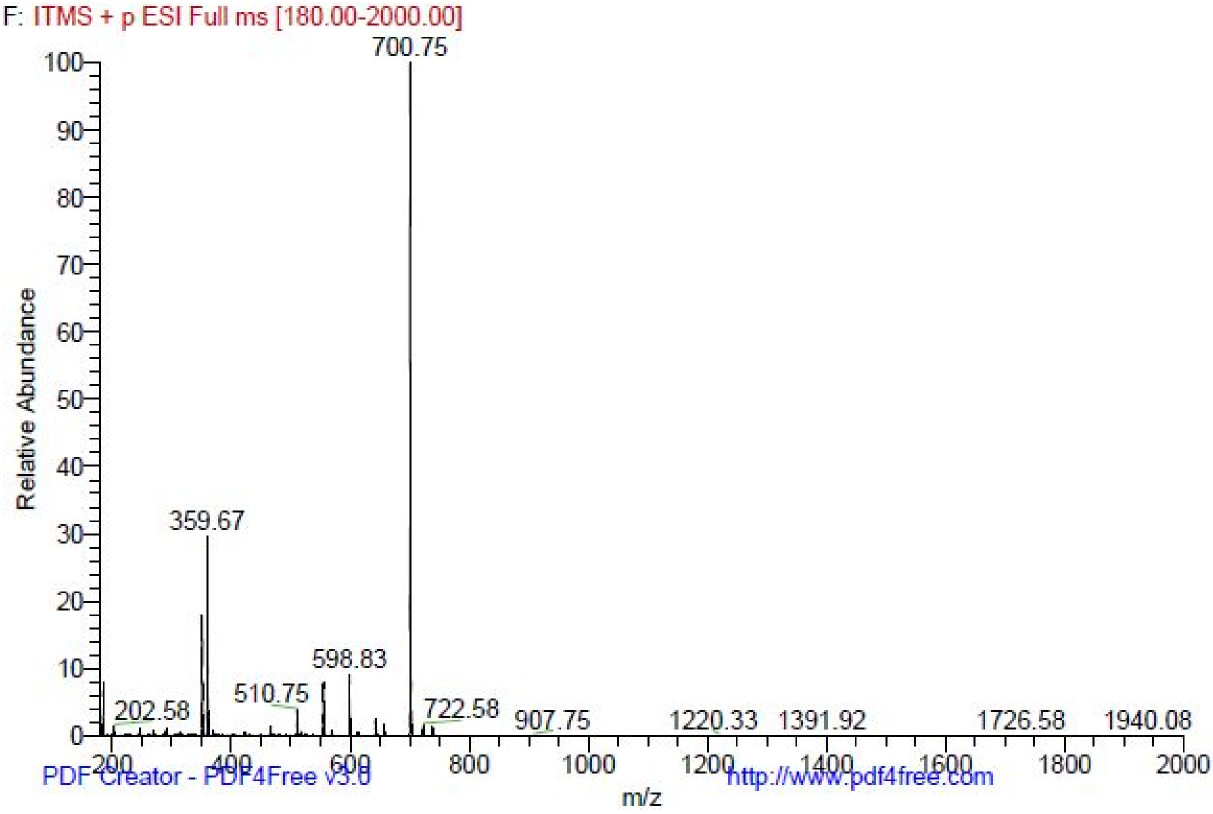

Figure S29. ESI-MS spectrum (ESI+, H_2O:CH_3CN = 1:1) of compound **7d**.

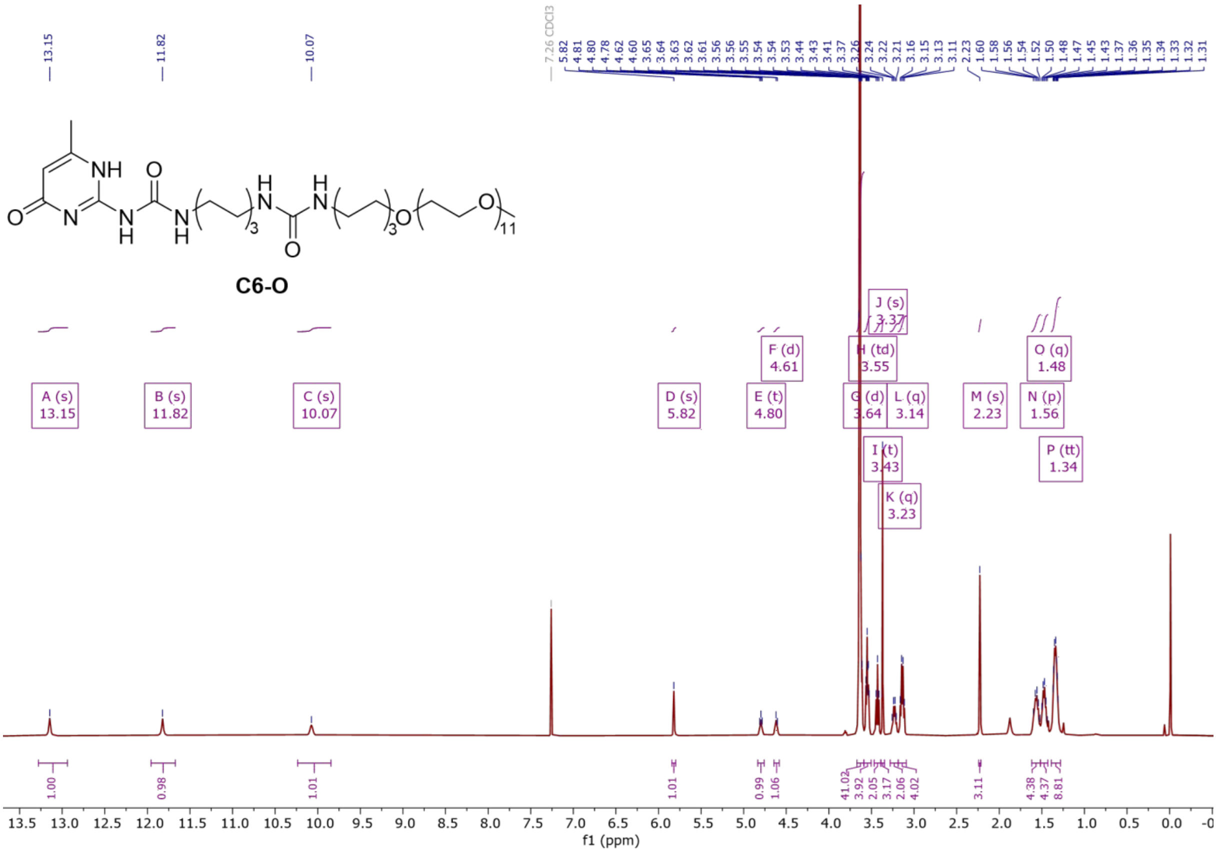

Figure S30. ^{1}H-NMR of compound **C6-O** in $CDCl_3$.

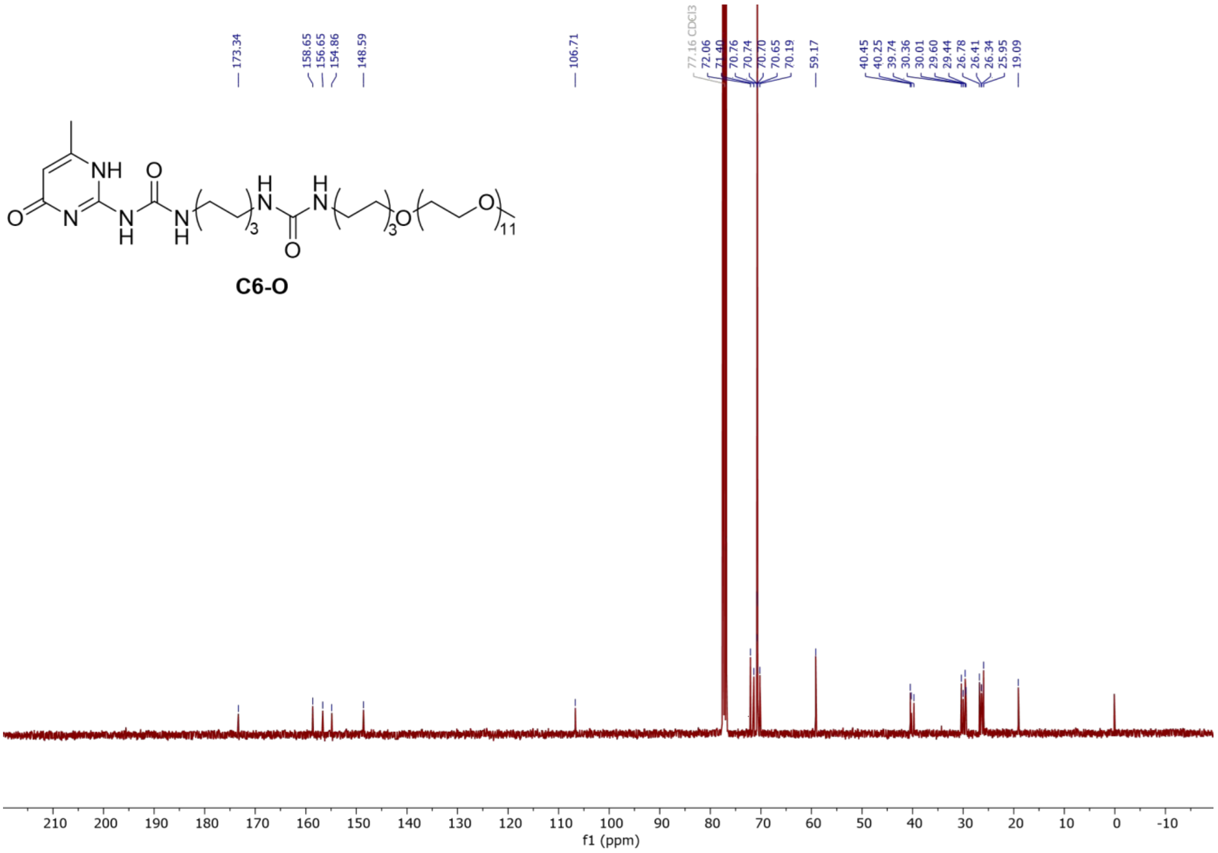

Figure S31. ^{13}C-NMR of compound **C6-O** in $CDCl_3$.

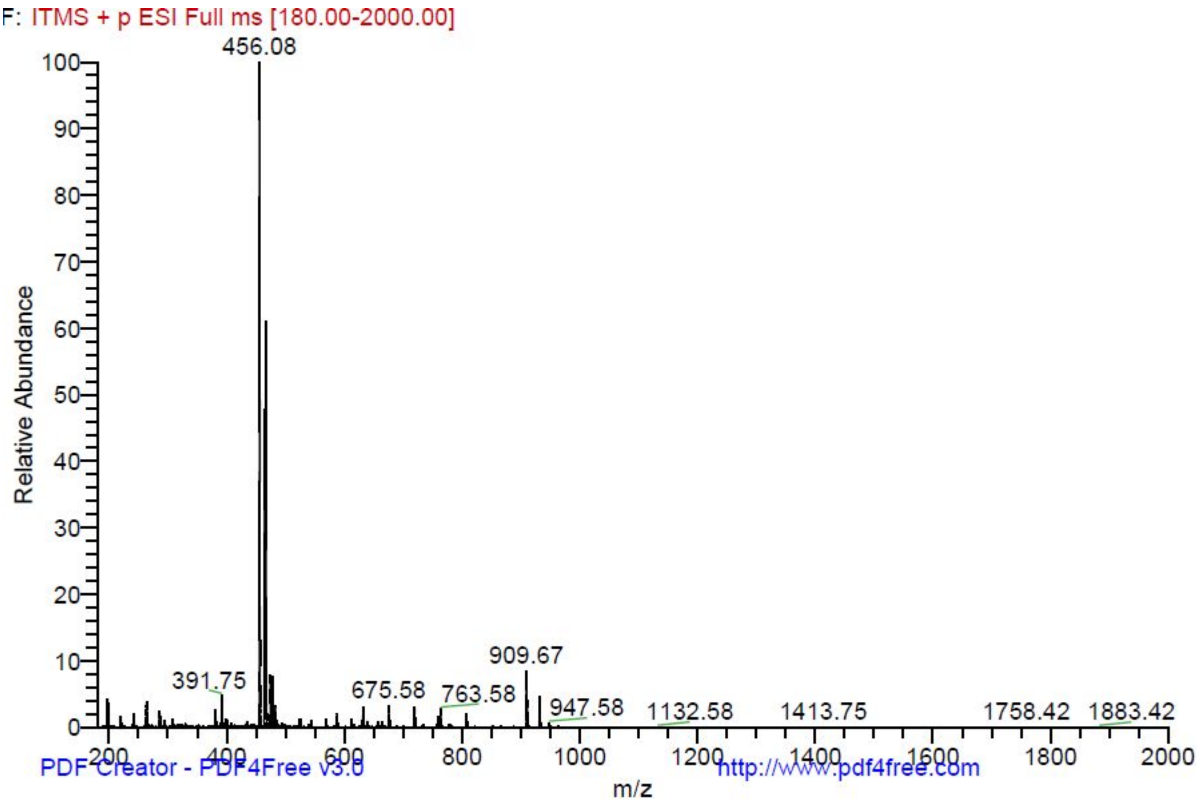

Figure S32. ESI-MS spectrum (ESI+, $H_2O:CH_3CN$ = 1:1) of compound **C6-O**.

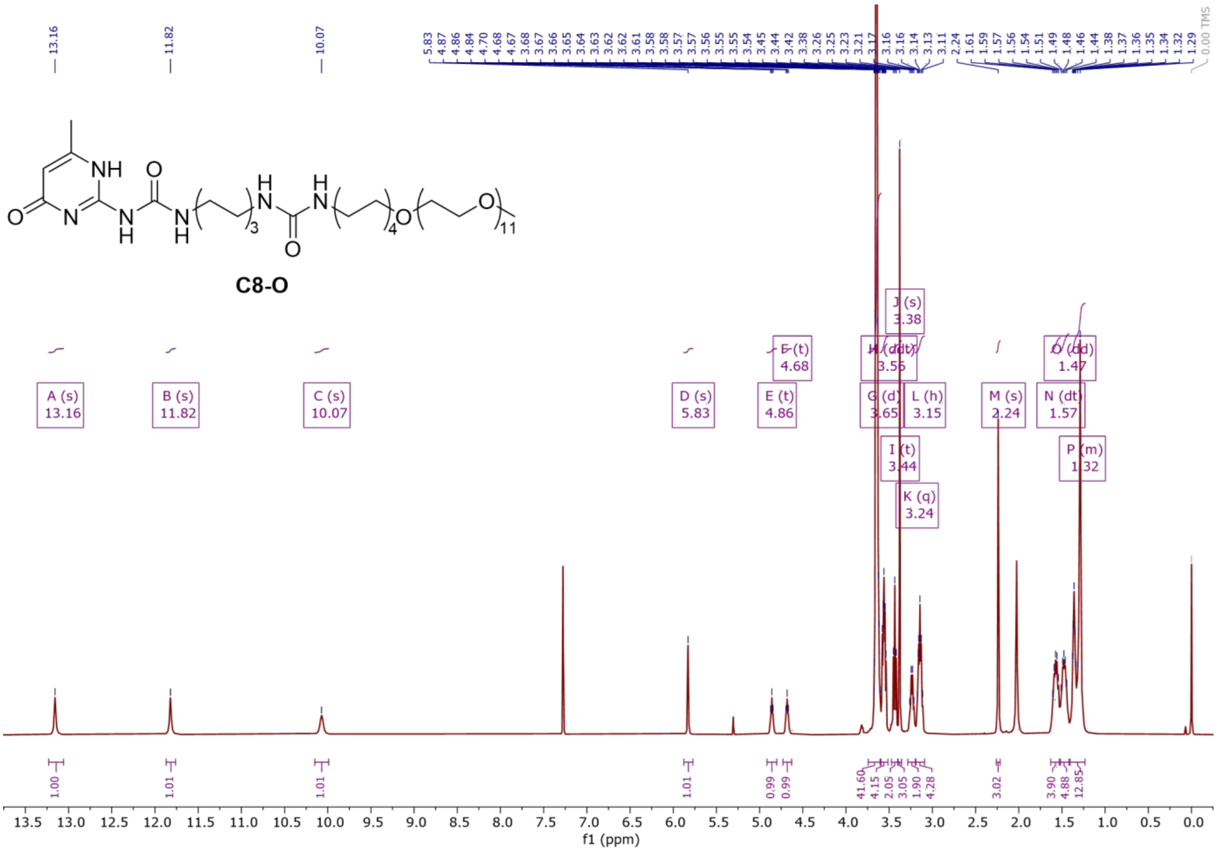

Figure S33. ^{1}H-NMR of compound **C8-O** in $CDCl_3$.

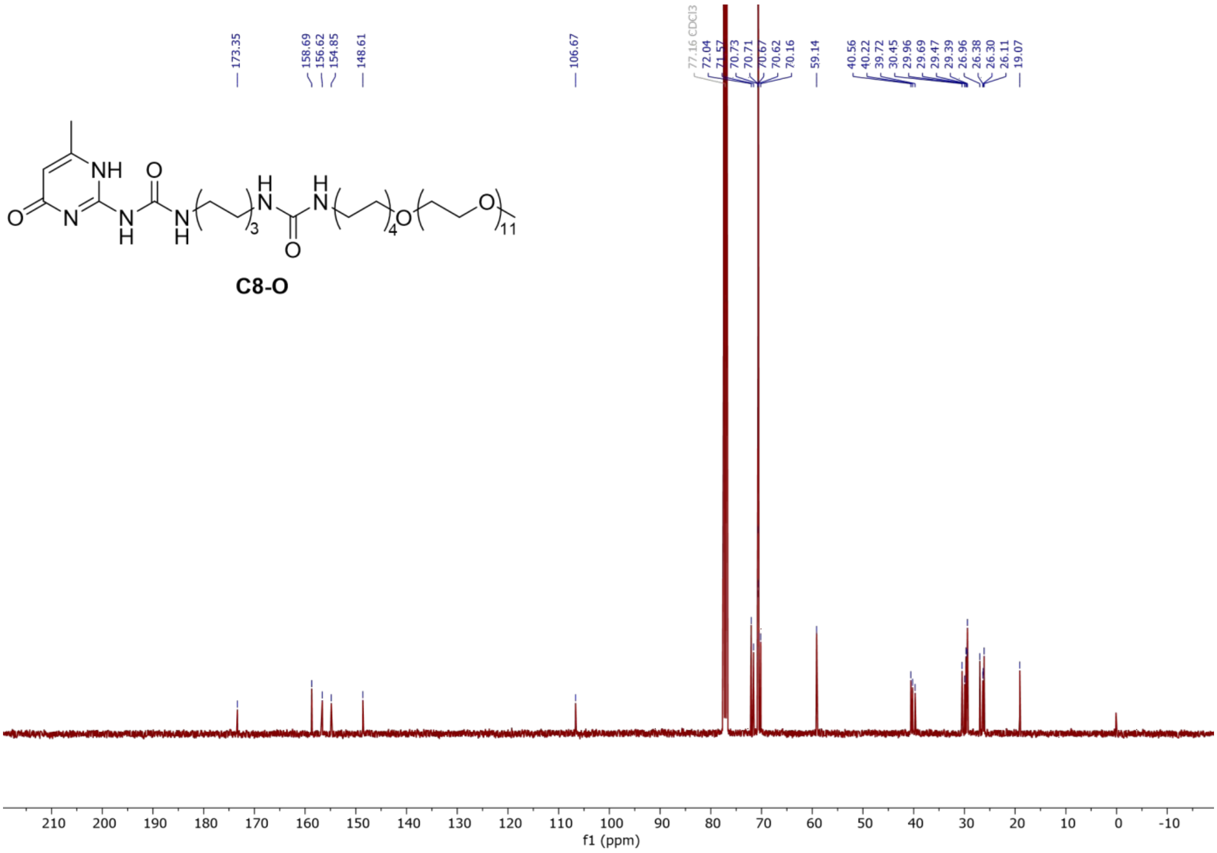

Figure S34. ^{13}C-NMR of compound **C8-O** in $CDCl_3$.

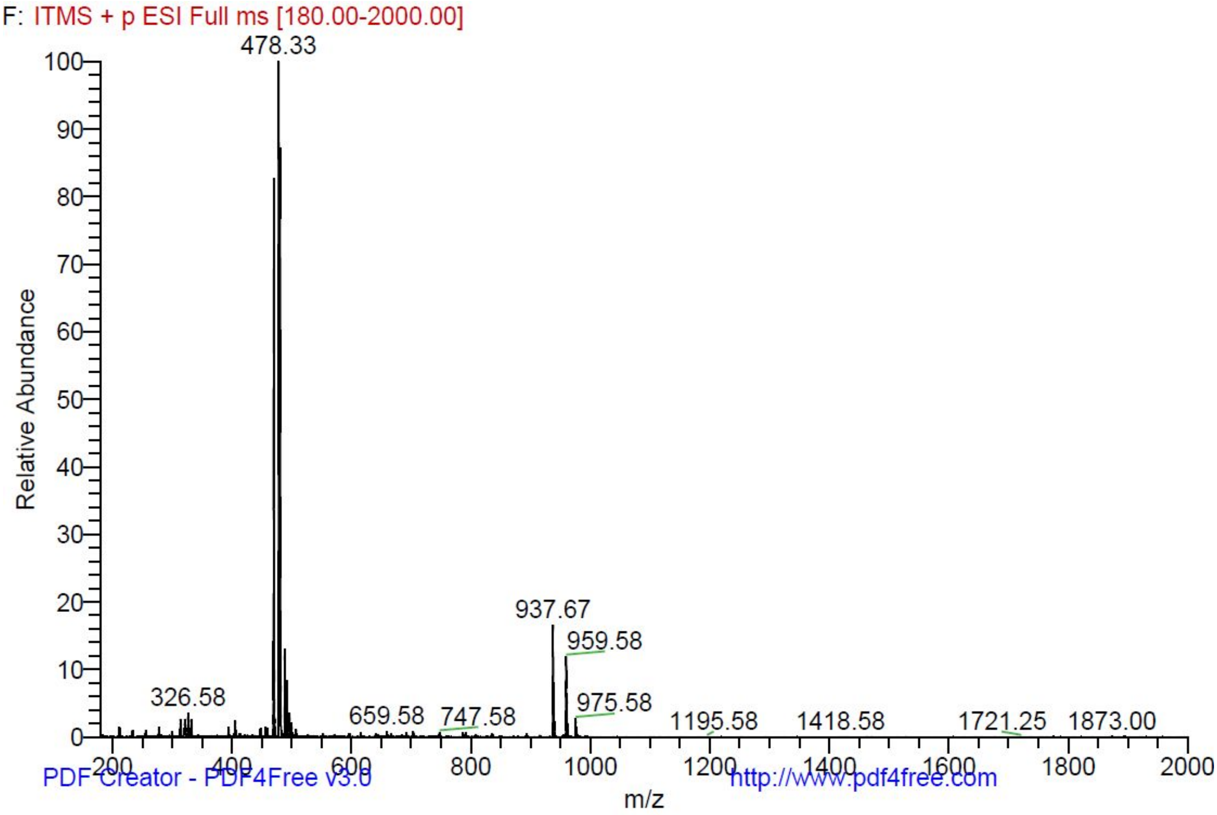

Figure S35. ESI-MS spectrum (ESI+, $H_2O:CH_3CN$ = 1:1) of compound **C8-O**.

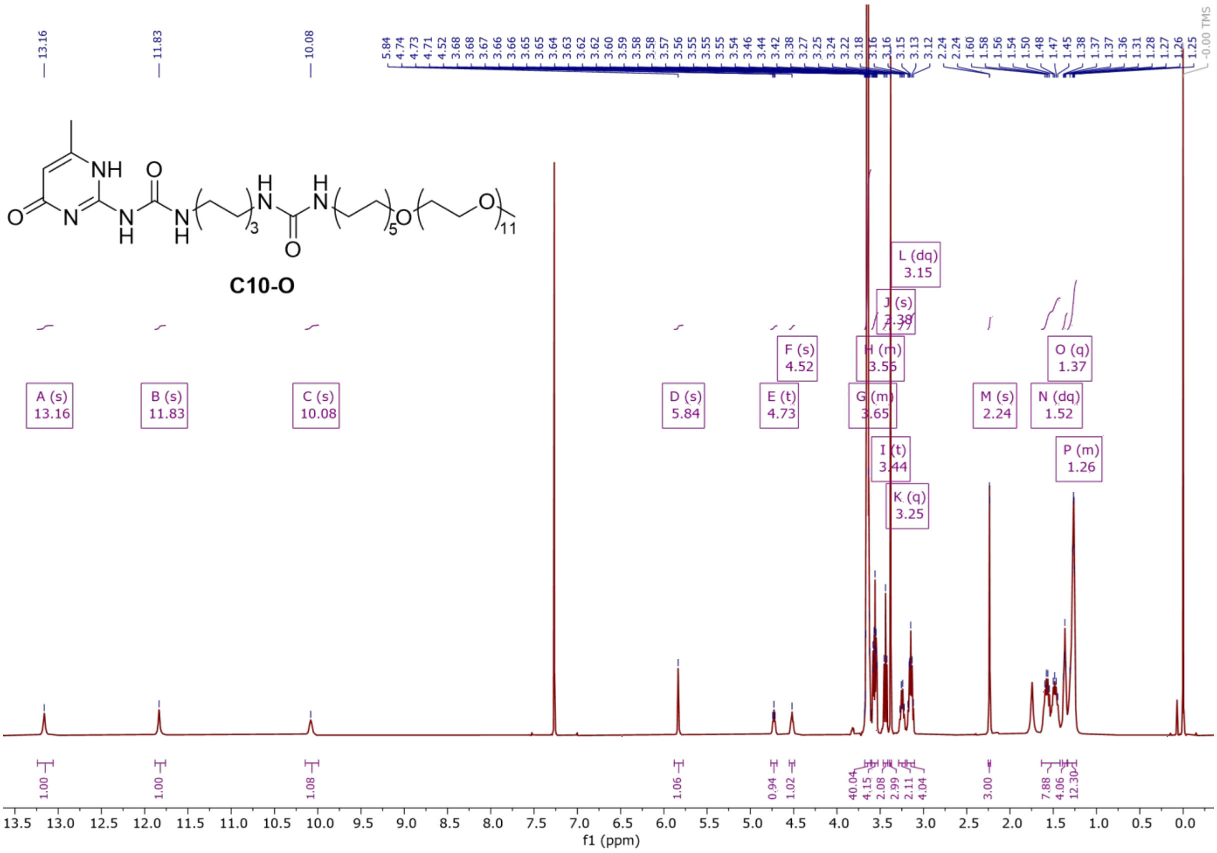

Figure S36. ^{1}H-NMR of compound **C10-O** in $CDCl_3$.

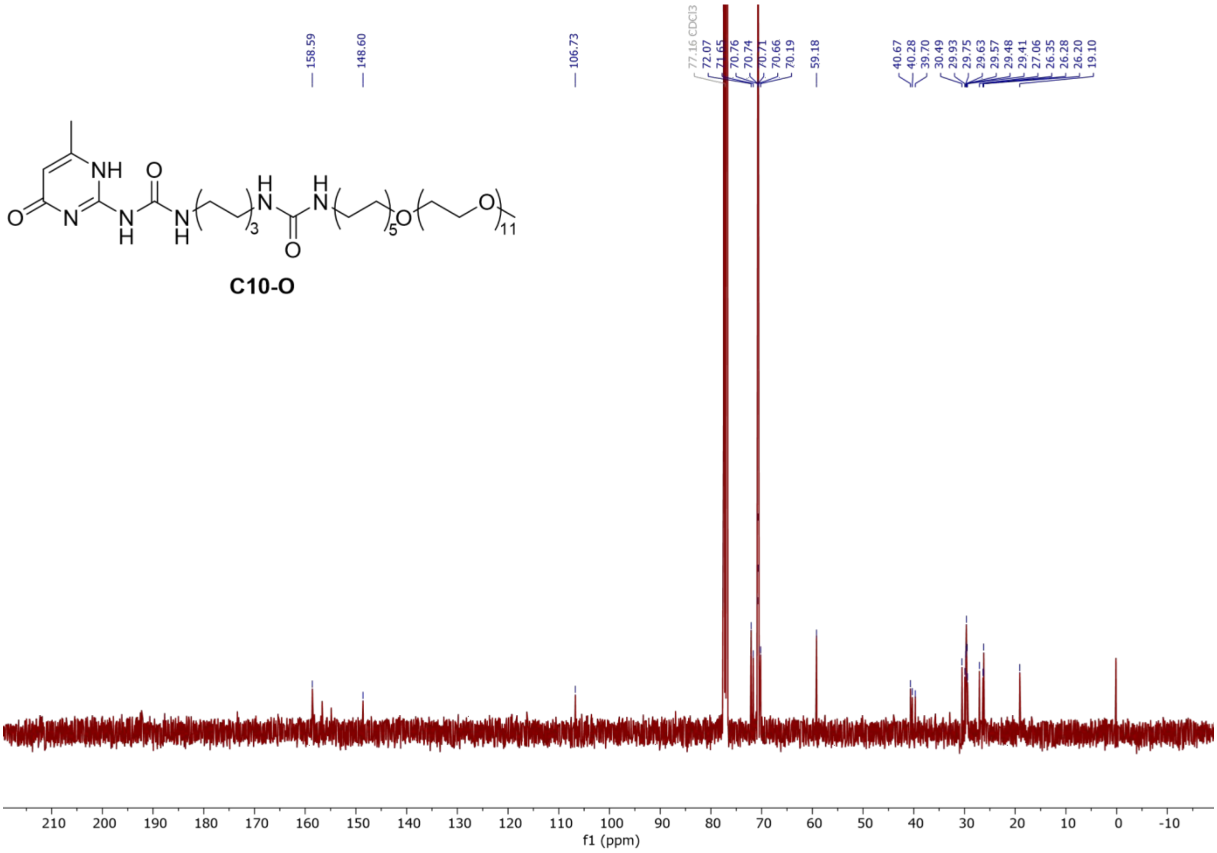

Figure S37. ^{13}C-NMR of compound **C10-O** in $CDCl_3$.

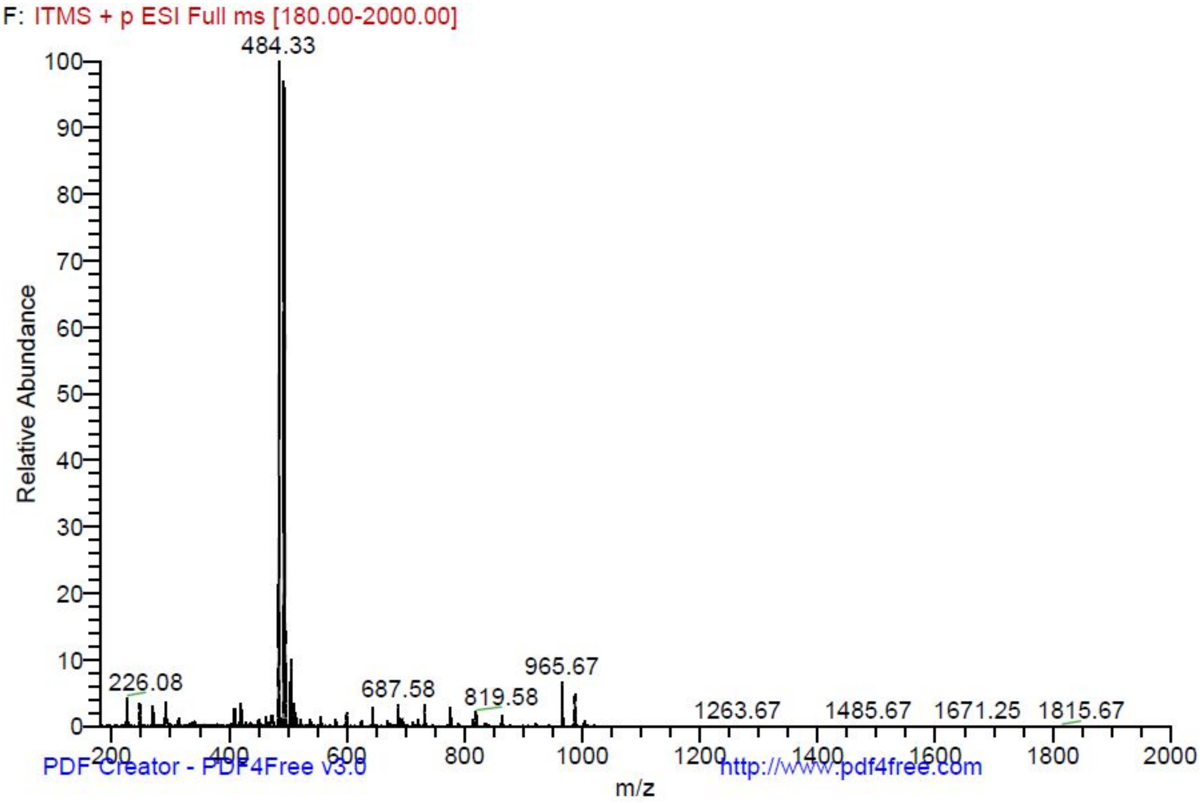

Figure S38. ESI-MS spectrum (ESI+, H_2O:CH_3CN = 1:1) of compound **C10-O**.

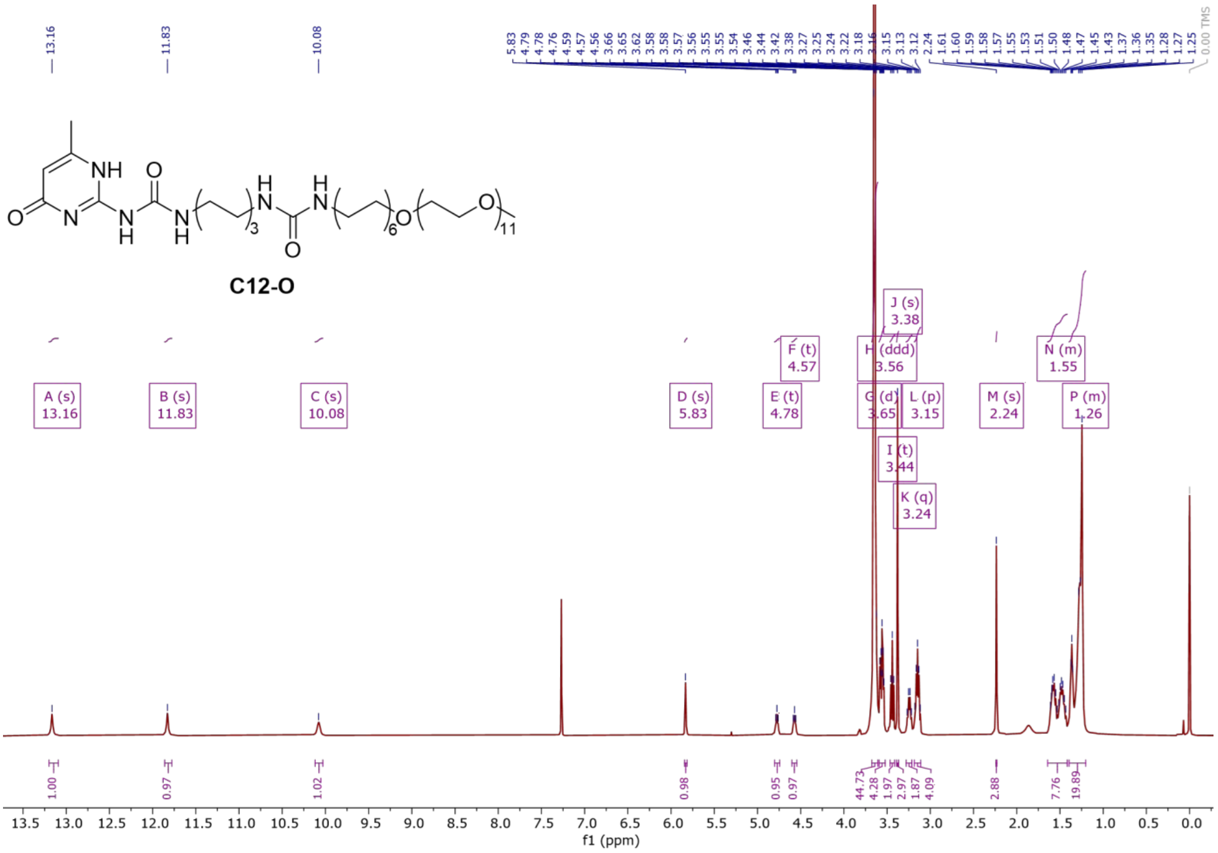

Figure S 39. ^{1}H-NMR of compound **C12-O** in $CDCl_3$.

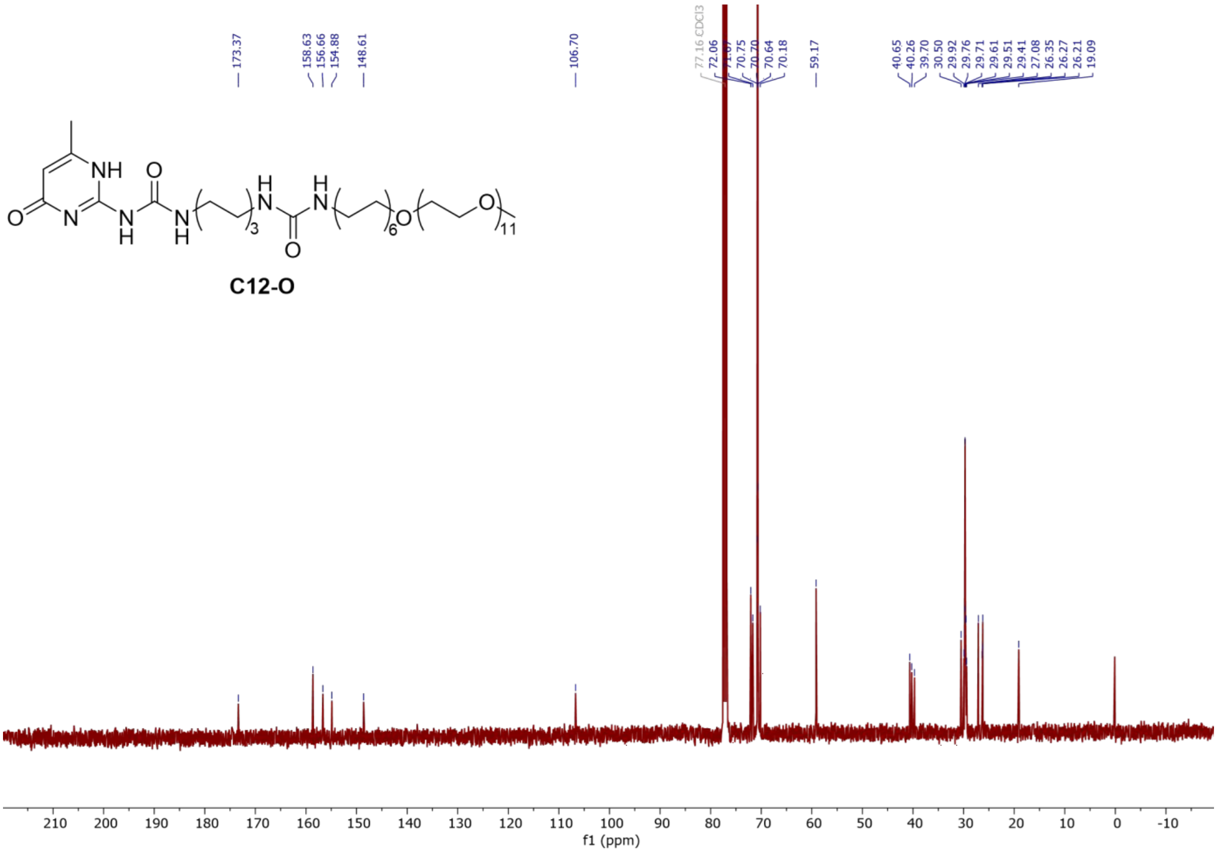

Figure S40. ^{13}C-NMR of compound **C12-O** in $CDCl_3$.

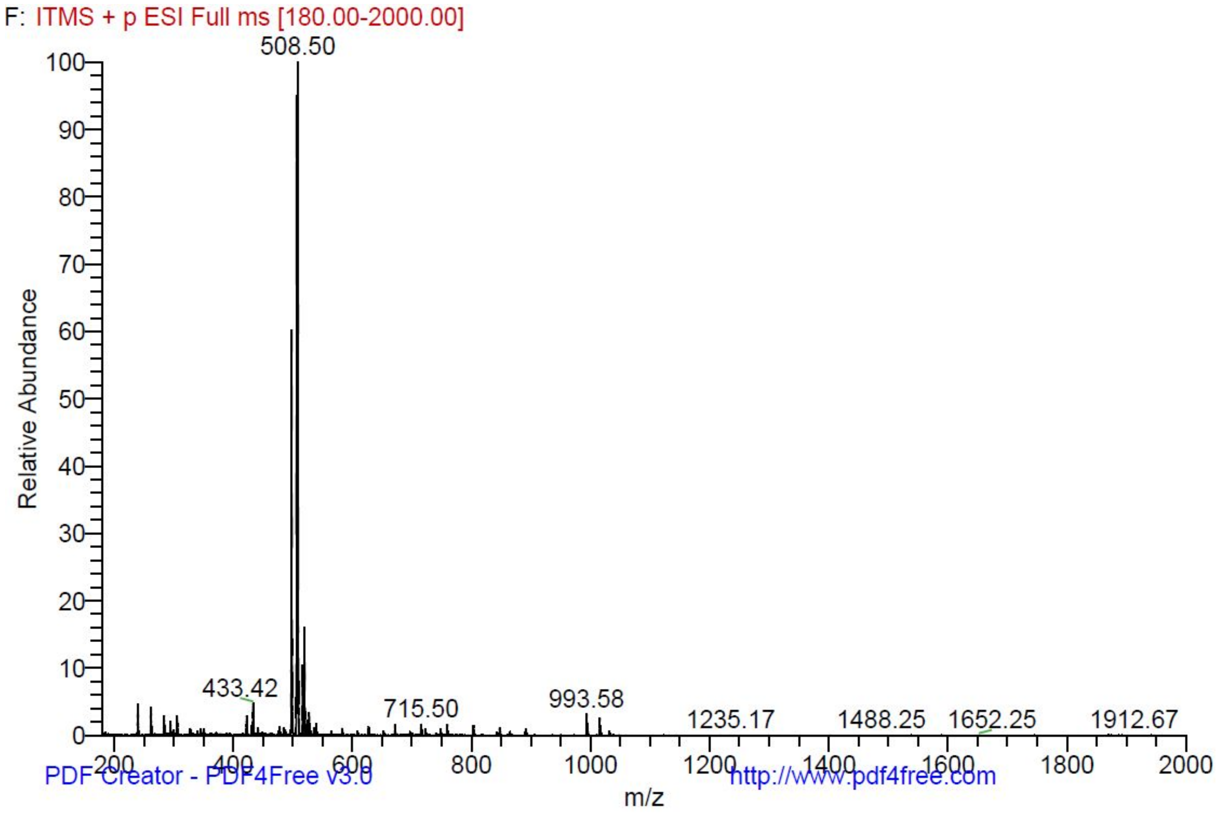

Figure S41. ESI-MS (ESI+, H_2O:CH_3CN = 1:1) spectrum of compound **12-O**.

3 UV-Vis

3.1 VT-UV Vis spectra

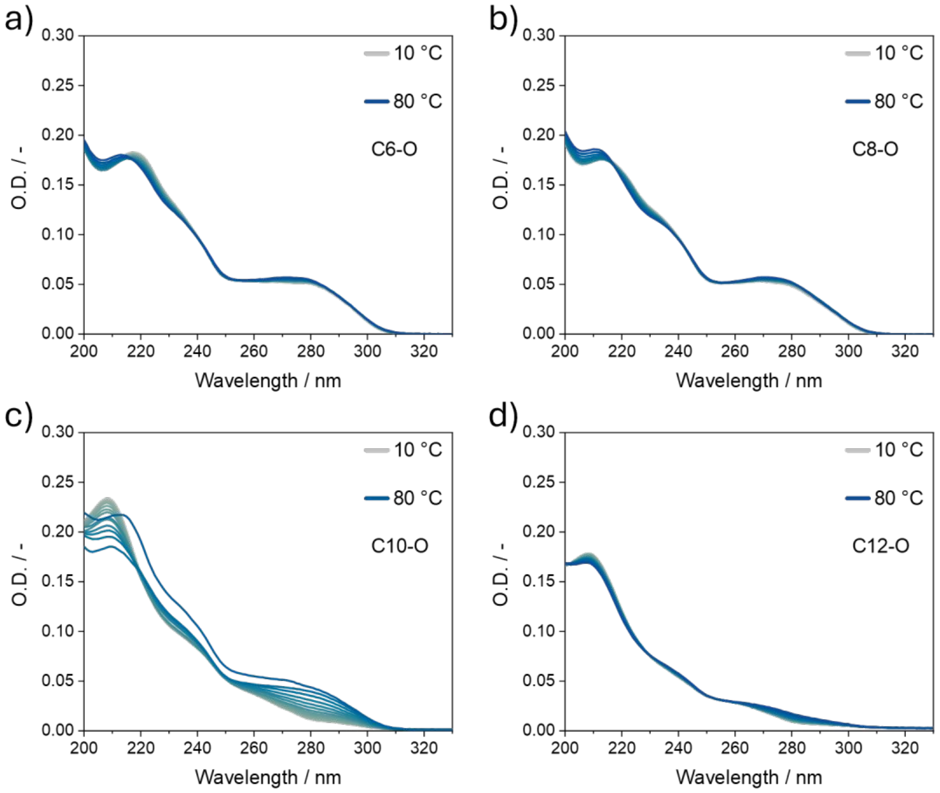

Figure S42. Variable temperature UV-Vis spectra of the (a) **C6-O**, (b) **C8-O**, (c) **C10-O** and (d) **C12-O** in MQ water (C = 100 μM, 1 °C·min^{-1}, *l* = 1 mm).

3.2 UV-Vis and pH-dependent UV-Vis spectra

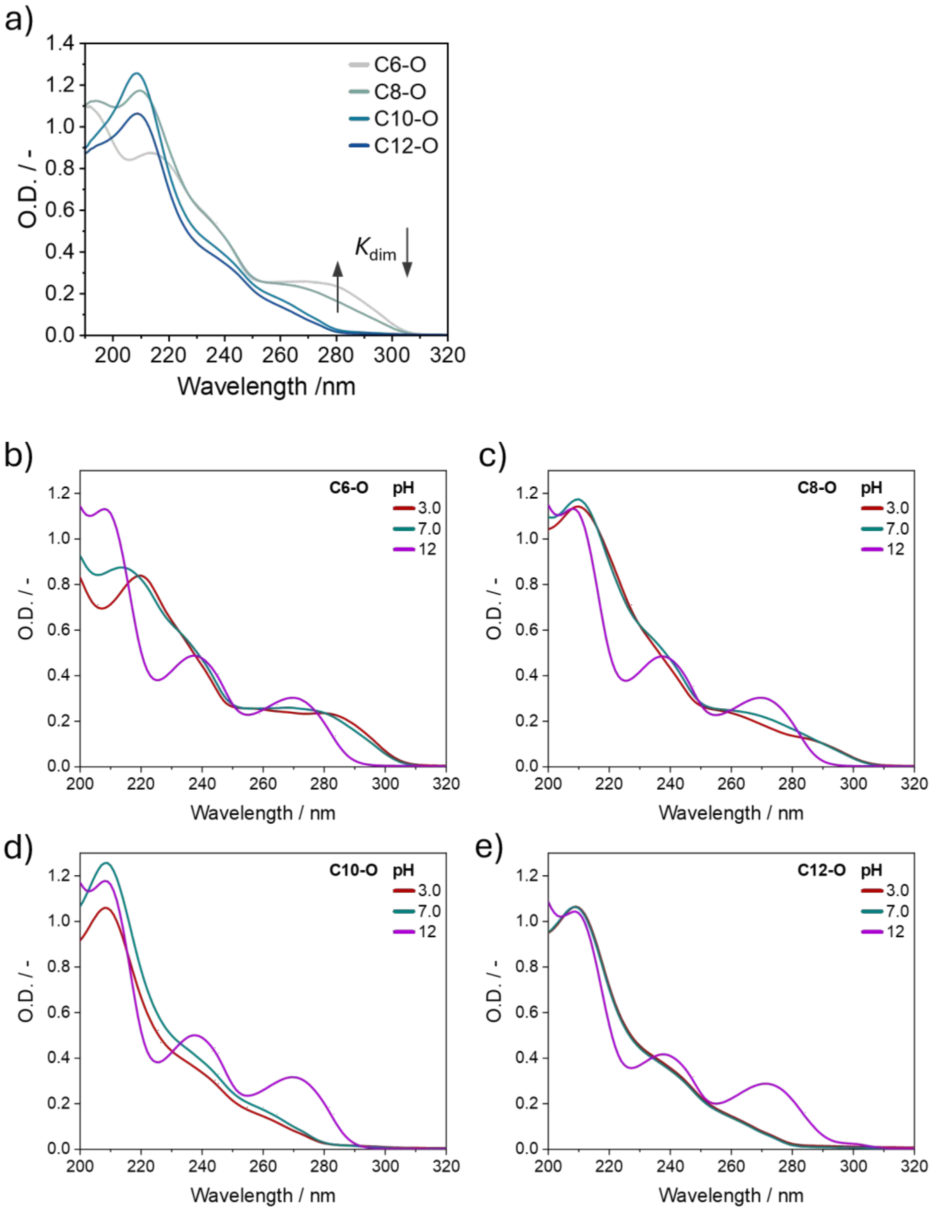

Figure S43. (a) UV-Vis spectra of the synthesized molecules (C = 100 µM, T = 20 °C, *l* = 1 mm) at neutral pH. pH-dependent UV-Vis spectra of the (b) **C6-O**, (c) **C8-O**, (d) **C10-O** and (e) **C12-O** in MQ water (C = 500 µM, T = 20 °C, *l* = 1 mm) at pH 3.0 (red line), pH 7.0 (green line) and pH 12 (purple line).

4 ^{1}H-NMR spectroscopy

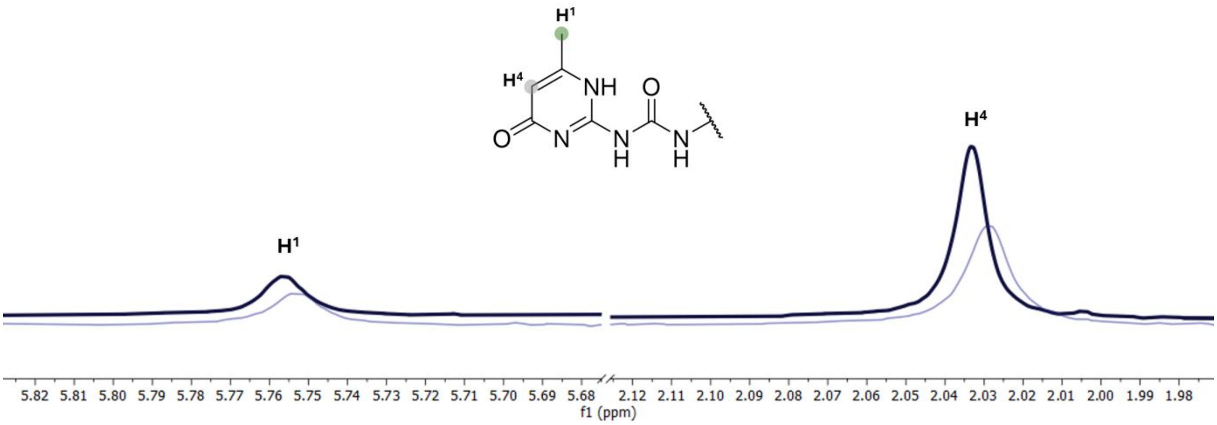

Figure S44. Partial ^{1}H-NMR spectra of **C6-O** (dark blue line) and **C8-O** (light blue line) at in D_2O (C = 500 μM, T = 25 °C) highlighting the signals corresponding to the alkylidene proton and methyl group of the UPy ring.

4.1 VT-NMR spectra

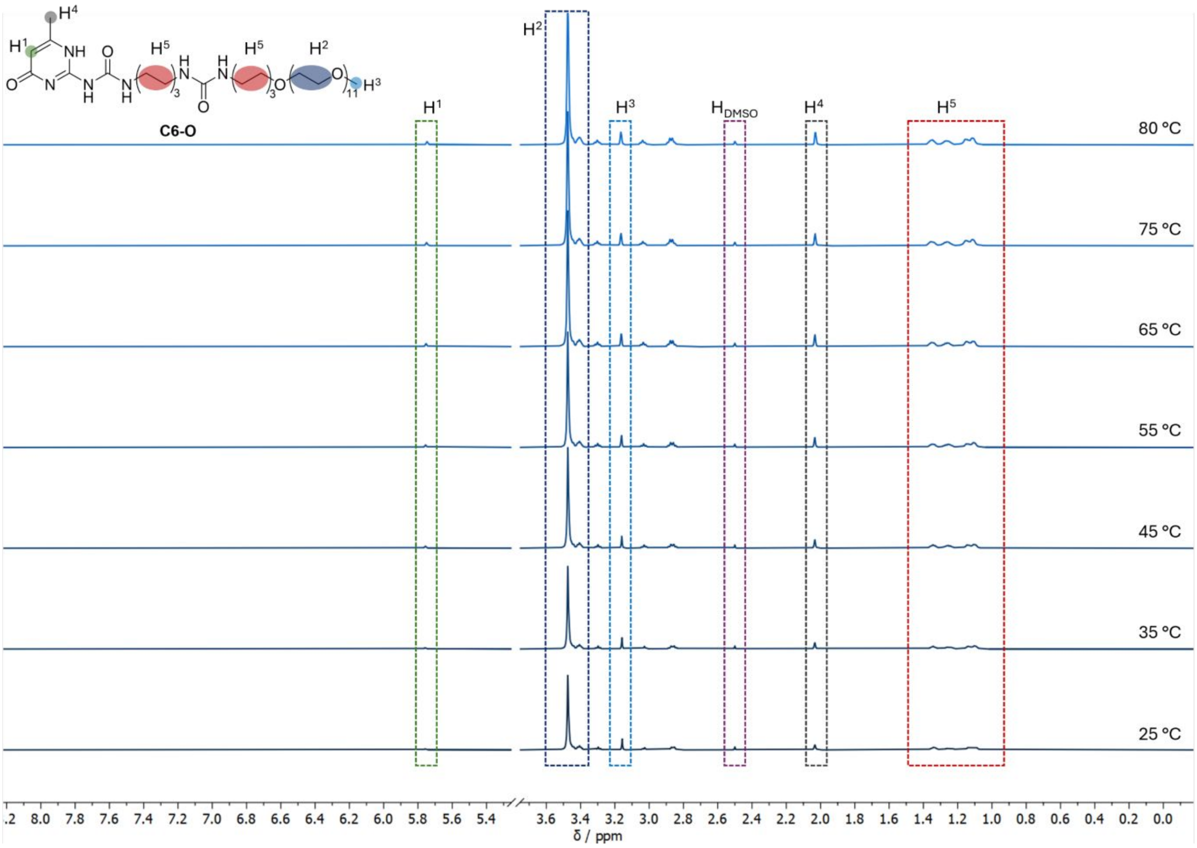

Figure S45. VT-NMR of **C6-O** (C = 500 μM) in D_2O.

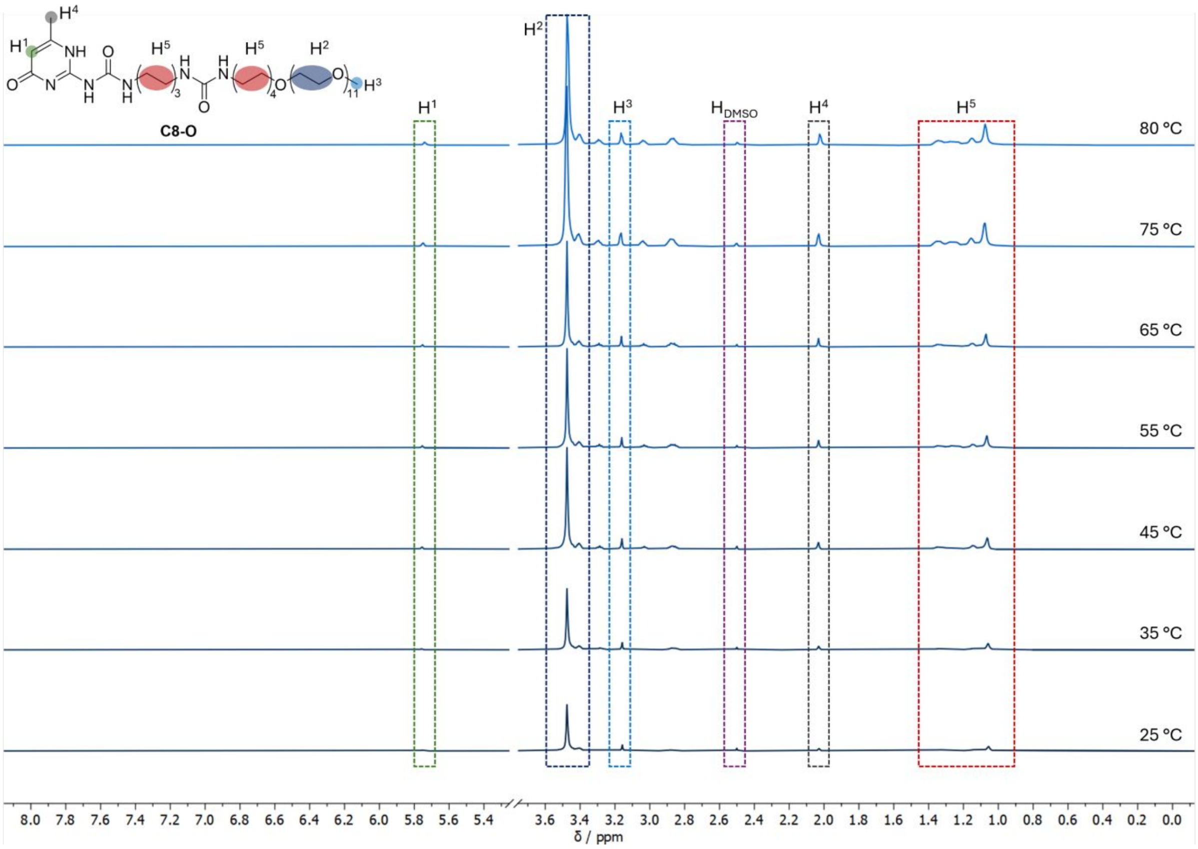

Figure S46. VT-NMR of **C8-O** (C = 500 μM) in D_2O.

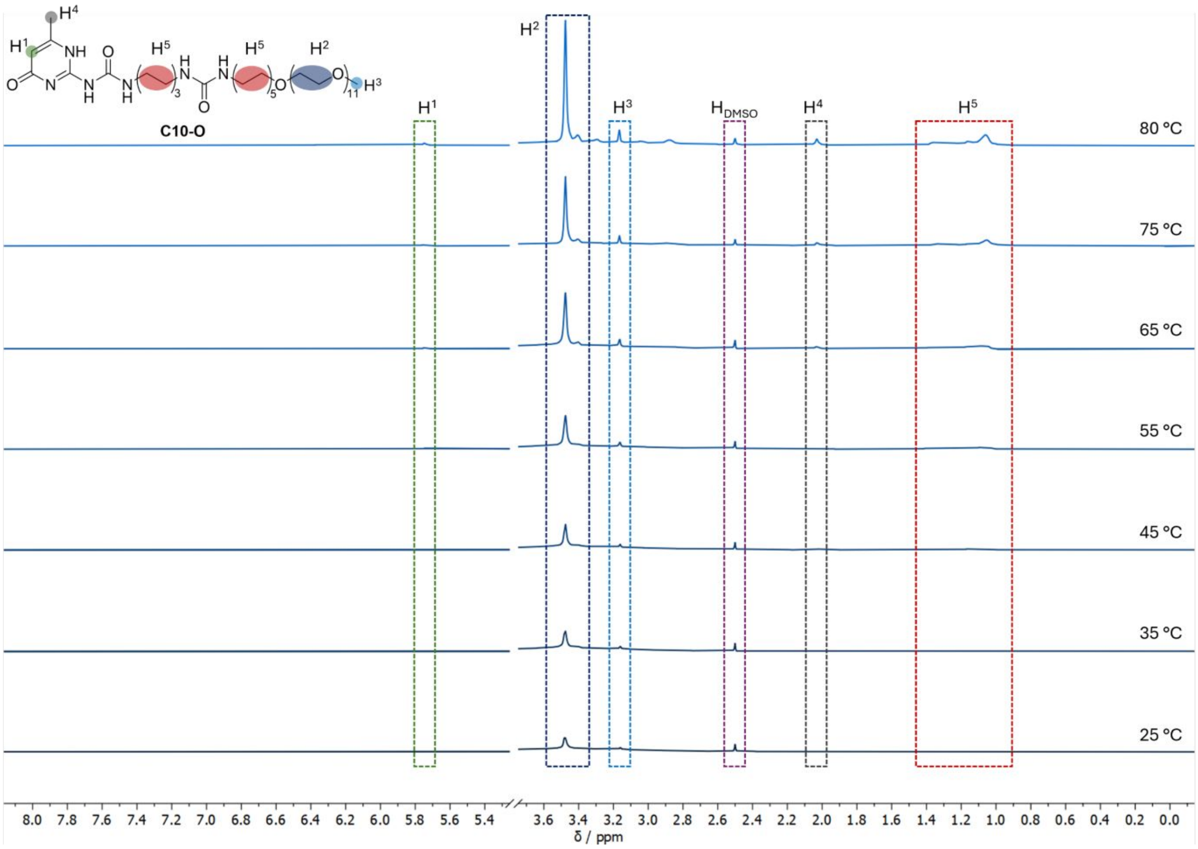

Figure S47. VT-NMR of **C10-O** (C = 500 μM) in D_2O.

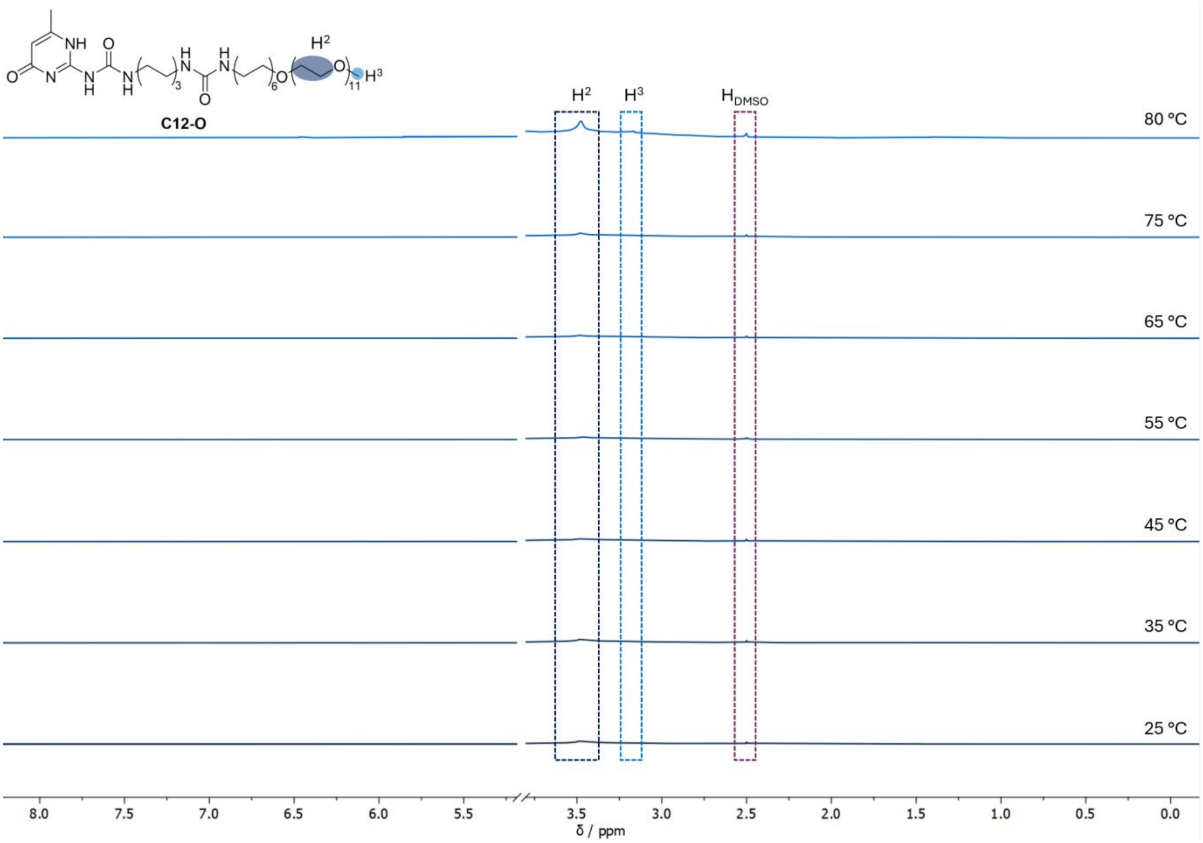

Figure S48. VT-NMR of **C12-O** (C = 500 μM) in D_2O.

4.2 pH dependent ^{1}H-NMR spectra

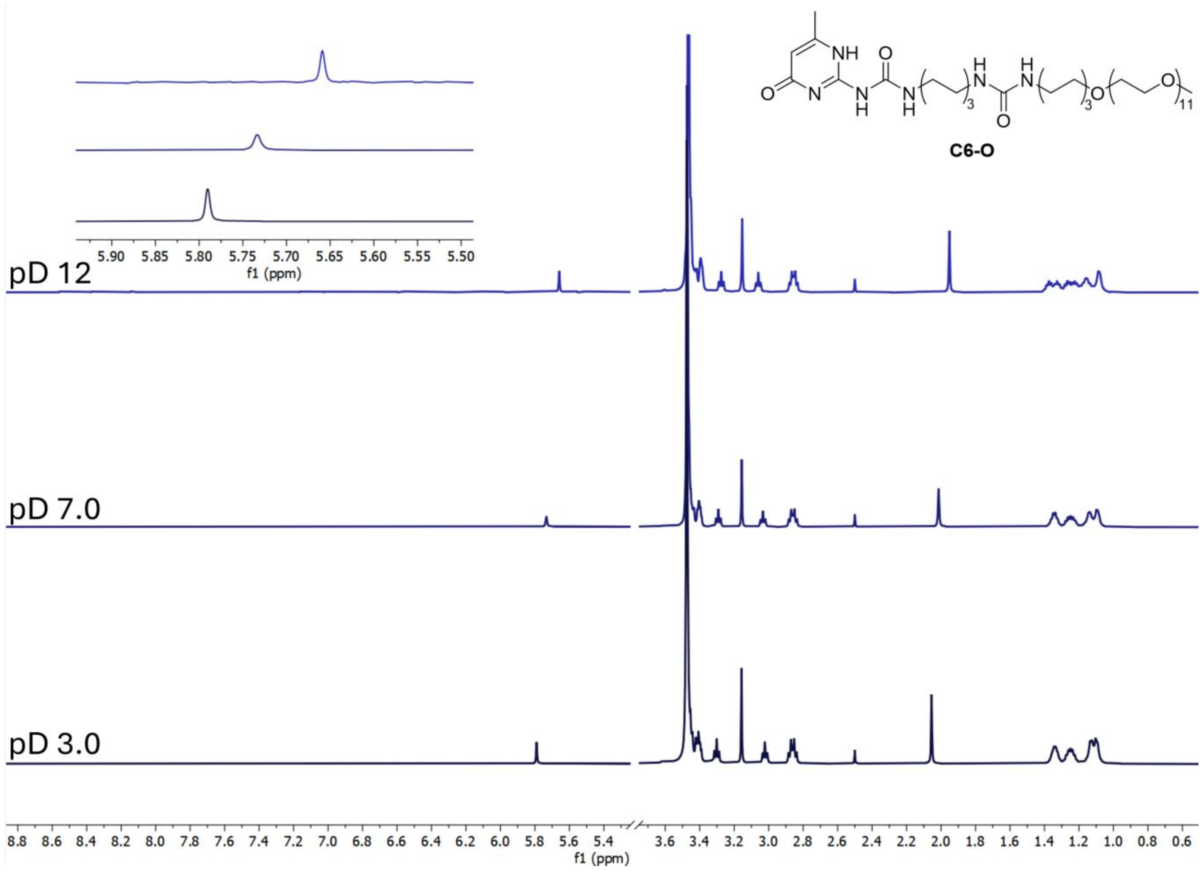

Figure S49. ^{1}H-NMR spectra of **C6-O** at pD 3.0 (bottom), pD 7.0 (middle) and pD 12 (top) (C = 500 μM).

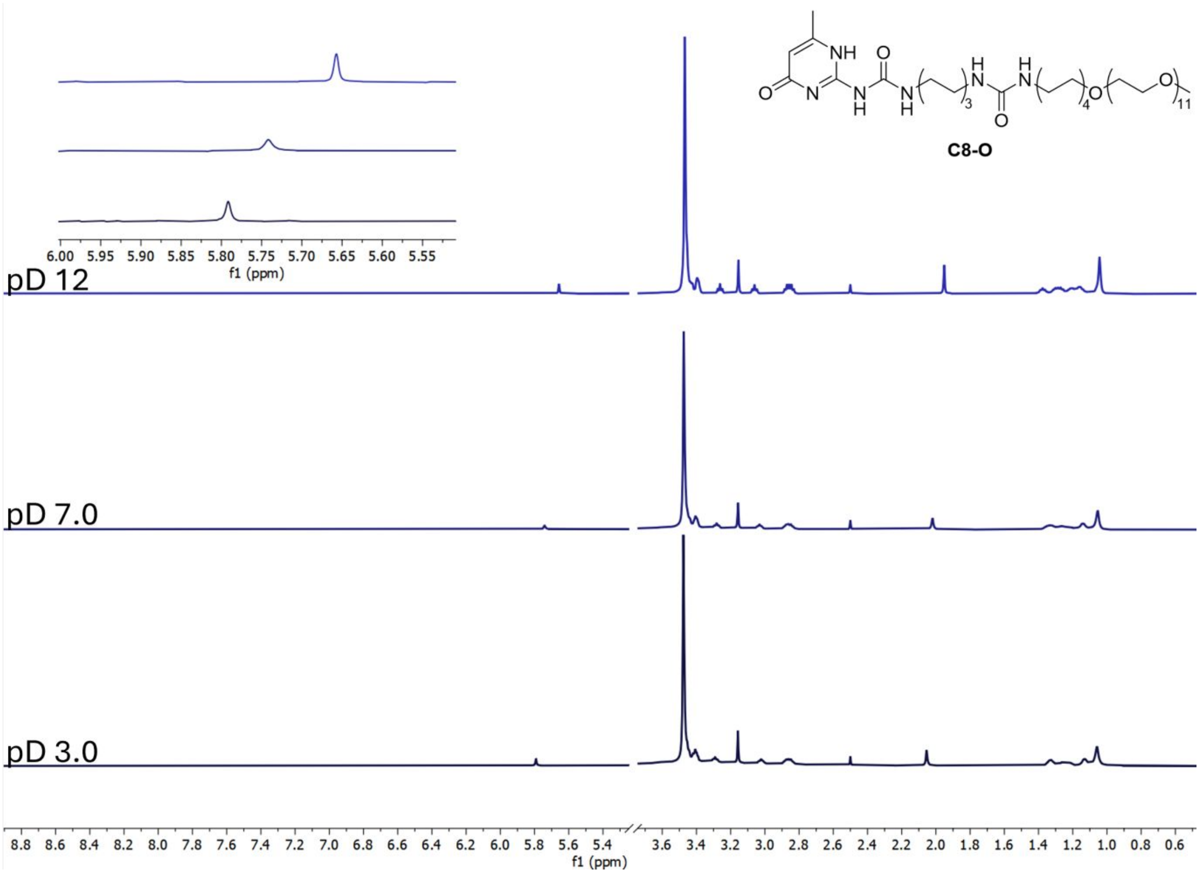

Figure S50. ^{1}H-NMR spectra of **C8-O** at pD 3.0 (bottom), pD 7.0 (middle) and pD 12 (top) (C = 500 μM).

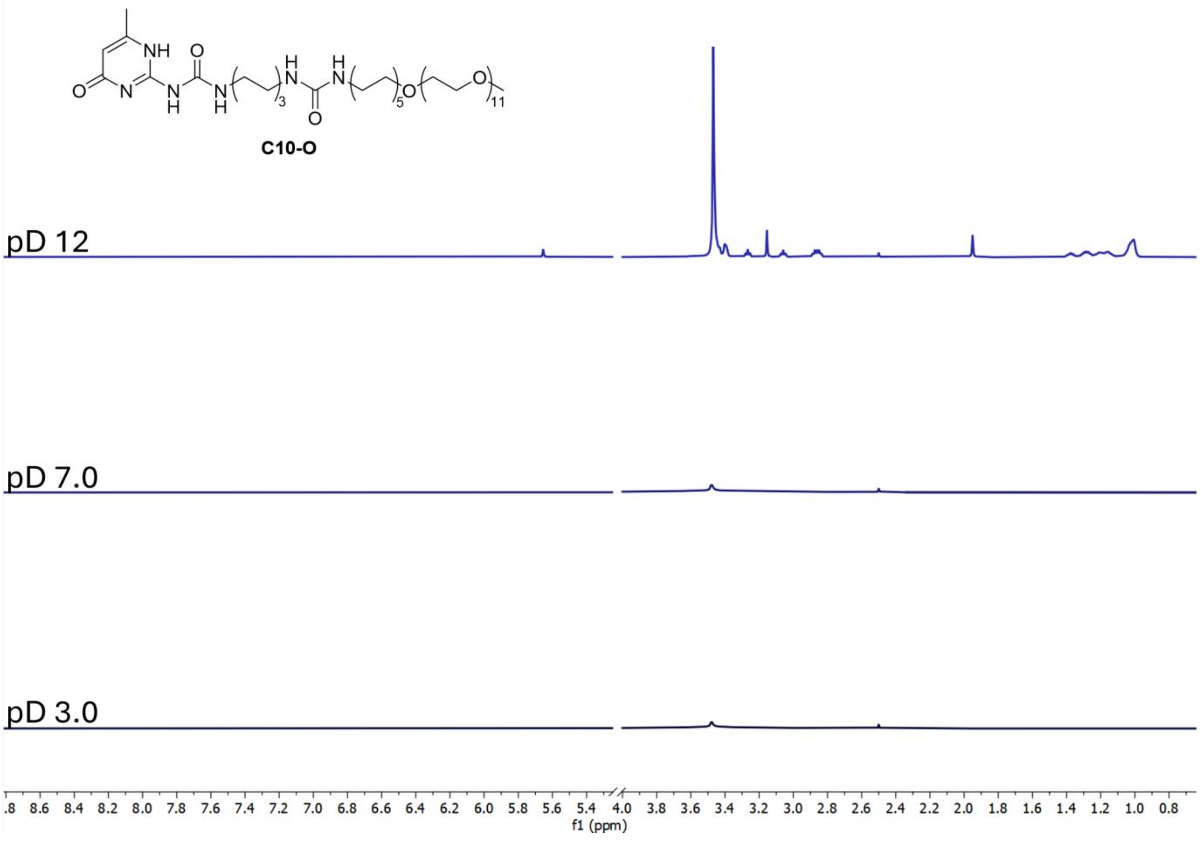

Figure S51. ^{1}H-NMR spectra of **C10-O** at pD 3.0 (bottom), pD 7.0 (middle) and pD 12 (top) (C = 500 μM).

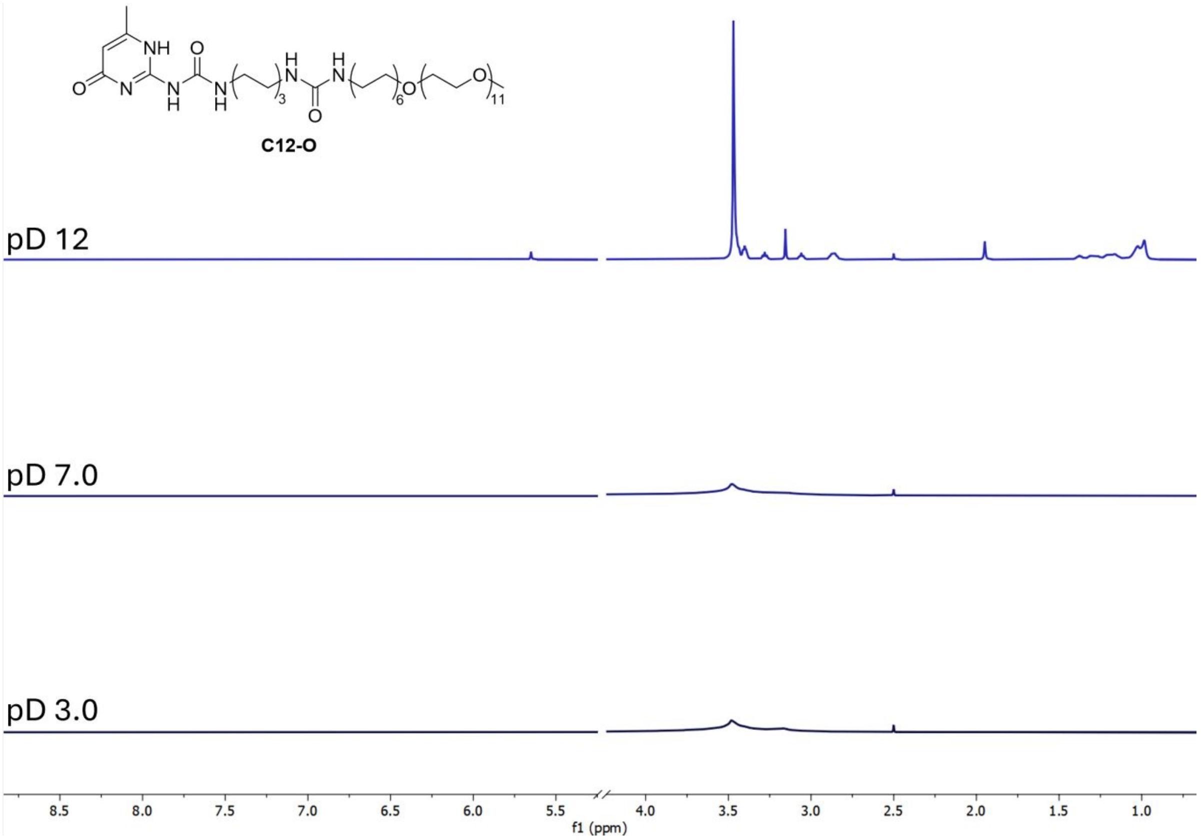

Figure S52. ^{1}H-NMR spectra of **C12-O** at pD 3.0 (bottom), pD 7.0 (middle) and pD 12 (top) (C = 500 μM).

5 Nile Red Fluorescence assay at different pHs

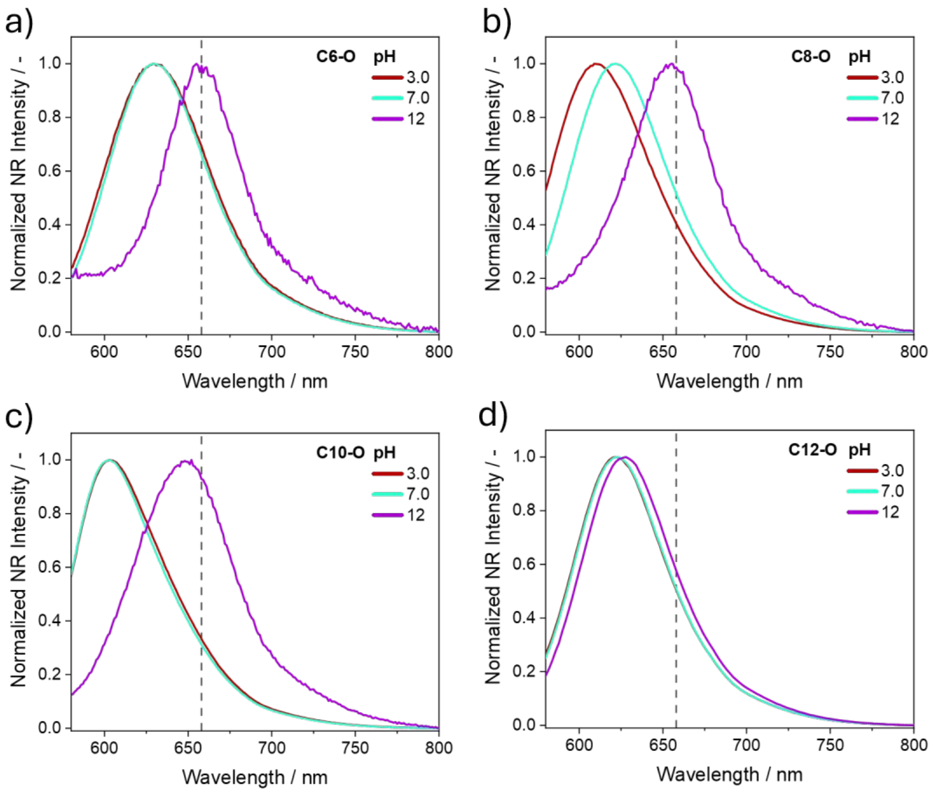

Figure S53. Nile Red emission spectra of (a) **C6-O**, (b) **C8-O**, (c) **C10-O** and (d) **C12-O** at pH 3.0, pH 7.0 and pH 12 (C = 500 µM, λ_{ex} = 550 nm, T = 20 °C, *l* = 10 mm).

6 SAXS analysis

Table S6. Summary of the parameters obtained from the fits of each sample in MQ water (C = 2.5 mM, pH 3.0). A power law model was added to every fit.

**Molecule**	**C6-O**	**C8-O**	**C10-O**	**C12-O**
Model	Flexible Cylinder	Elliptical Cylinder	Elliptical Cylinder	Elliptical Cylinder
Background	0.0091188	0.013261	0.011693	0.012668
A Scale	0.00023155	0.00044647	0.00049409	0.00033133
A Radius Minor / Å	-	24.422	29.187	25.453
A Radius / Å	24.035	-	-	-
A Axis Ratio	-	2.8537	2.5277	2.219
A Length / Å	1719.4	666.01	18063	17694
A Kuhn Length / Å	429.85	-	-	-
B Scale	1.2436e-08	1.0967e-07	7.833e-08	8.577e-08
B Power	3.6189	3.2737	3.3154	3.3037
X^2	0.62217	1.3511	1.6388	1.1009

Table S7. Summary of the parameters obtained from the fits of each sample in MQ water (C = 2.5 mM, pH 7.0). A power law model was added to every fit.

**Molecule**	**C6-O**	**C8-O**	**C10-O**	**C12-O**
Model	Cylinder	Elliptical Cylinder	Elliptical Cylinder	Elliptical Cylinder
Background	0.015501	0.013328	0.0085281	0.013074
A Scale	6.4794e-05	0.00043972	0.00044462	0.0003645
A Radius Minor / Å	-	24.526	30.484	27.216
A Radius / Å	82	-	-	-
A Axis Ratio	-	2.7254	2.1908	2.1571
A Length / Å	220	588.96	18112	17874
B Scale	7.8058e-08	7.2388e-08	1.0694e-08	1.423e-08
B Power	3.37	3.3449	3.6228	3.5799
X^2	2.2816	0.8519	0.8184	0.9889

Table S8. Summary of the parameters obtained from the fits of each sample in MQ water (C = 2.5 mM, pH 12.0). A power law model was added to every fit.

**Molecule**	**C6-O**	**C8-O**	**C10-O**	**C12-O**
Model	Lamellar	Sphere	Sphere	Sphere
Background	0.010748	0.012686	0.01447	0.01198
A Scale	0.00023916	1.9611e-08	1.9655e-08	4.3556e-08
A power		3.5283	3.5396	3.4039
Thickness / Å	46.984	-	-	-
A Radius / Å	-	30	43.1	38.8
B Scale	4.2402e-08	9.2891e-05	4.3898e-05	6.9179e-05
B power	3.4054	-	-	-
X^2	0.947	2.0152	1.0311	1.6089

Table S9. Summary of the parameters obtained from the fits of each sample in MQ water (C = 1.0 mM, pH 3.0). A power law model was added to every fit.

**Molecule**	**C6-O**	**C8-O**	**C10-O**	**C12-O**
Model	Cylinder	Flexible Cylinder	Elliptical Cylinder	Elliptical Cylinder
Background	0.013953	0.011801	0.012605	0.014404
A Scale	0.00010907	9.077e-05	0.00016602	0.00014743
A Radius Minor / Å	-	-	26.478	25
A Radius / Å	20.122	33.903	-	-
A Axis Ratio	-	-	2.9378	2.5851
A Length / Å	9218.7	458.95	586.56	575
A Kuhn Length / Å	-	45.895	-	-
B Scale	5.1222e-08	1.171e-06	3.5143e-08	4.5505 e-08
B Power	3.4132	2.9625	3.4432	3.433
X^2	1.0301	2.7221	0.85307	1.1882

Table S10. Summary of the parameters obtained from the fits of each sample in MQ water (C = 1.0 mM, pH 7.0). A power law model was added to every fit.

Molecule	**C6-O**	**C8-O**	**C10-O**	**C12-O**
Model	Cylinder	Flexible Cylinder	Elliptical Cylinder	Elliptical Cylinder
Background	0.0010015	0.013692	0.011695	0.010854
A Scale	0.00021179	0.00018228	0.00015044	0.00015203
A Radius Minor / Å	-	-	27.534	25.054
A Radius / Å	37.606	33.453	-	-
A Axis Ratio	-	-	2.5101	2.3772
A Length / Å	739.96	677.23	581.89	572.52
A Kuhn Length / Å	-	618.58	-	-
B Scale	2.2885e-09	7.3296e-08	2.3703e-08	3.1188e-08
B Power	3.9182	3.366	3.5006	3.4858
X^2	0.56739	0.67813	0.73549	0.94498

Table S11. Summary of the parameters obtained from the fits of each sample in MQ water (C = 1.0 mM, pH 12). A power law model was added to every fit.

Molecule	**C6-O**	**C8-O**	**C10-O**	**C12-O**
Model	Sphere	Sphere	Sphere	Sphere
Background	0.014043	0.01458	0.014505	0.014104
A Scale	6.1676e-08	6.3275e-08	4.8723e-08	2.5999e-08
A power	3.3785	3.3727	3.4115	3.4967
B Scale	7.2548e-05	3.775e-05	3.9028e-05	2.9038e-05
B Radius / Å	29.984	34.8	35.9	47.8
X^2	1.0678	0.71694	0.74485	1.244

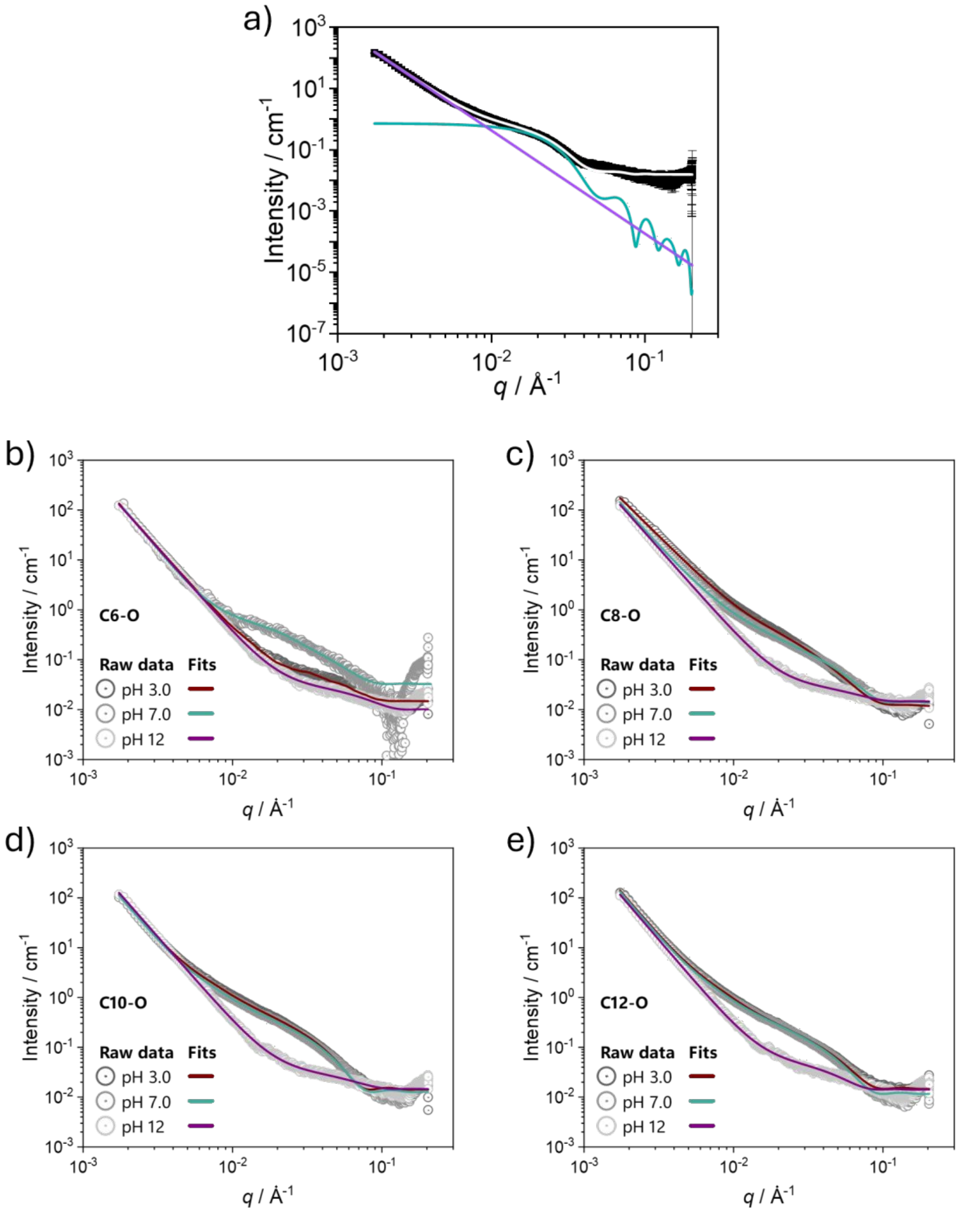

Figure S54. (a) Plot of the SAXS data for **C6-O** at a concentration of 2.5mM at pH 7 and fit (black data and white line respectively). The green data shows the contribution of the cylinder model to the fit and the purple lines shows the contribution of the power law component of the fit. Plot of the SAXS data (circles) and fits (solid lines) of (b) **C6-O**, (c) **C8-O**, (d) **C10-O** and (e) **C12-O** in MQ water at pH 3.0 (red line), 7.0 (green line) and 12 (purple line) (C = 1.0 mM).

7 HDX-MS analysis

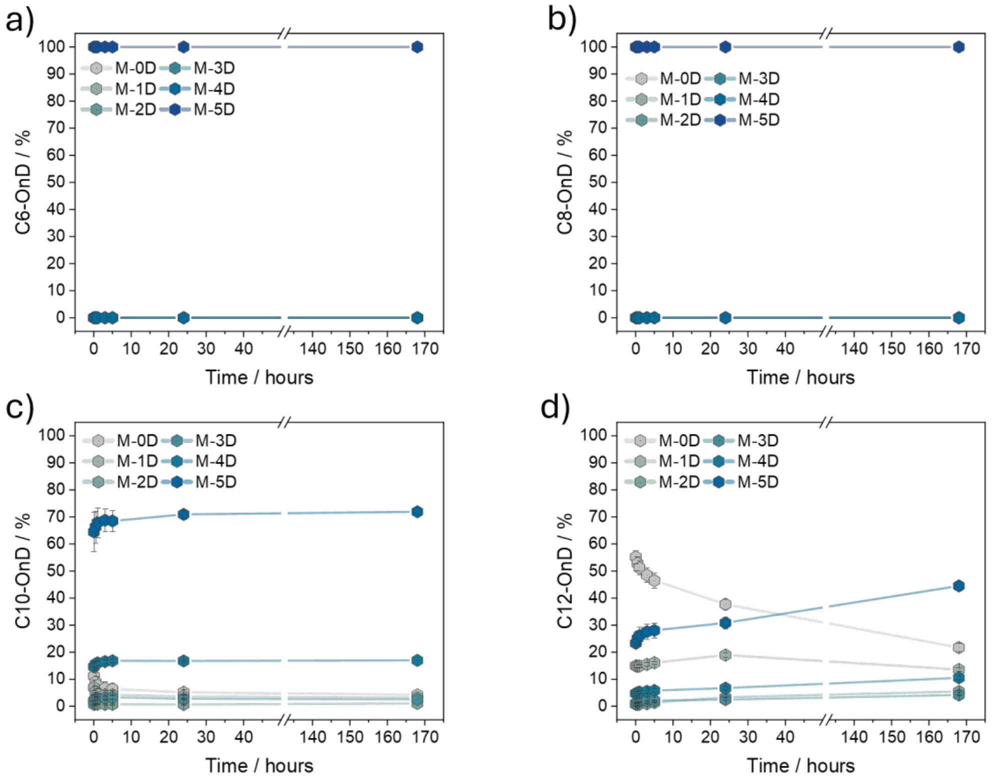

Figure S55. Percentage of each deuterated species (a) **C6-O**, (b) **C8-O**, (c) **C10-O** and (d) **C12-O** as a function of time after 25 × dilution of 2.5 mM aqueous samples in D_2O.

8 Rheological measurements

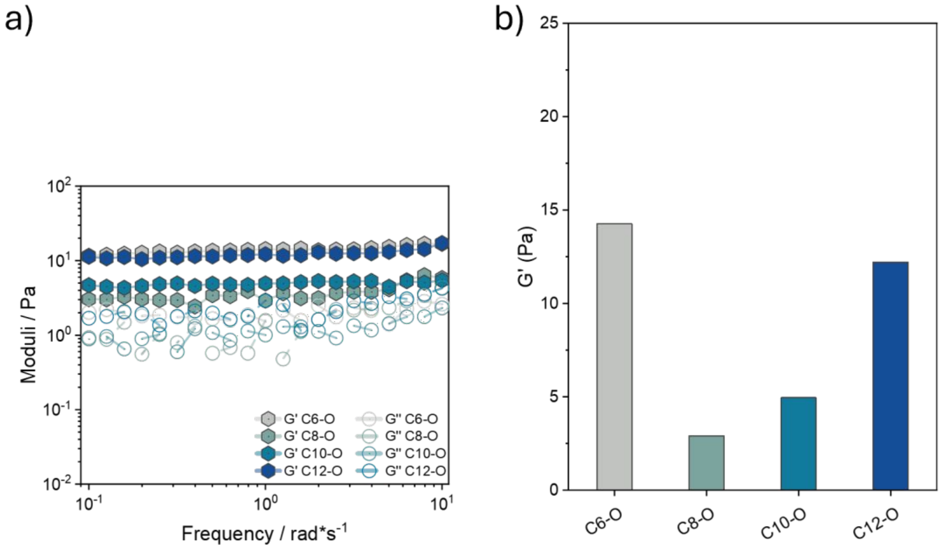

Figure S56. Rheology measurements of the different UPy molecules. (a) Frequency spectra showing the time dependent mechanical behavior of the different networks and (b) quantification of G′ at 1 rad / s and 1.0 % strain.

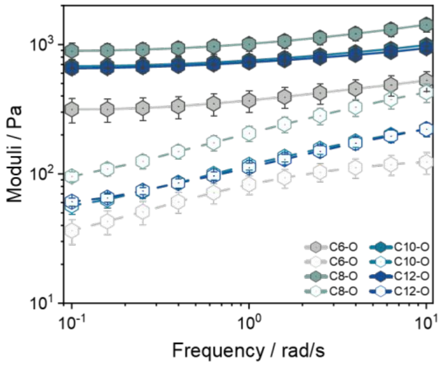

Figure S57*.* Frequency spectra showing the time dependent mechanical behavior (G′ as full symbols and G″ as empty symbols) of the different networks at UPy : BF = 80 : 1 ratio.

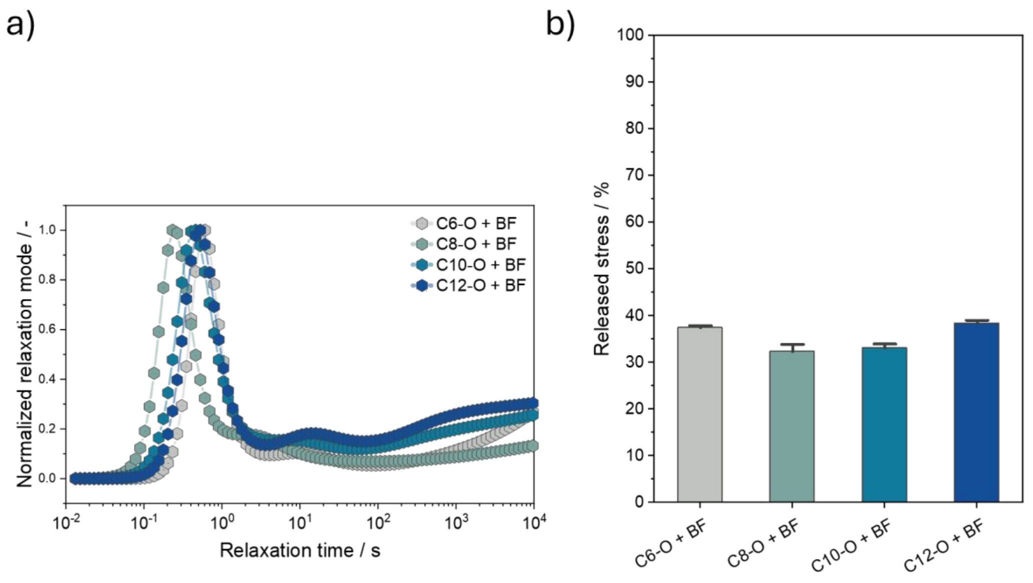

Figure S58. Quantification of stress relaxation for UPy : BF = 80 : 1 ratio. (a) Obtained relaxation spectra from the stress relaxation graph showing most important relaxation scales in the networks. (b) Quantification of released stress after 1000 s relaxation. Bar graph represents mean with SEM.

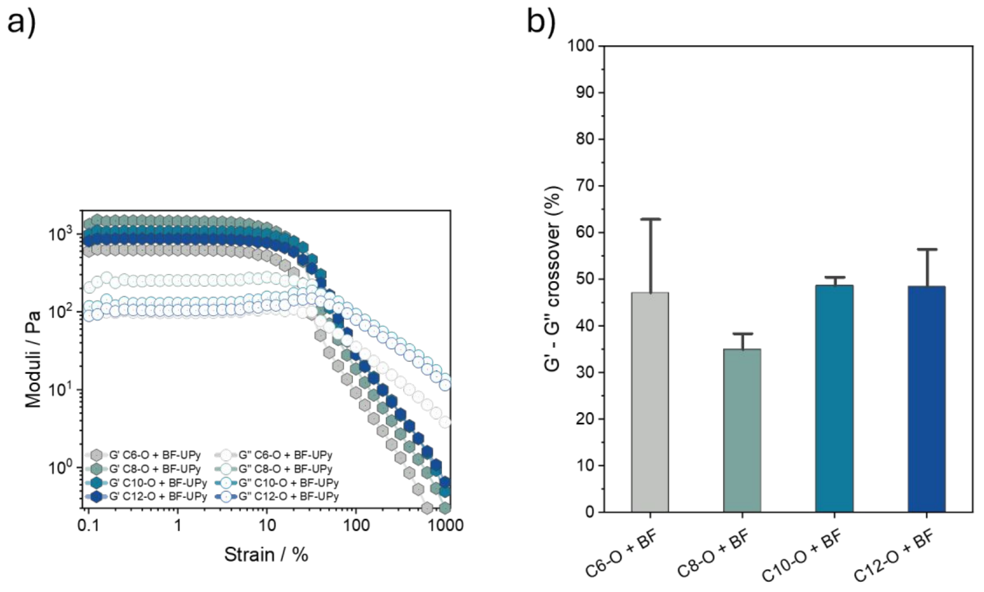

Figure S59. Strain sweep at 1 rad / s (a) obtained spectra for the different networks at UPy : BF = 80 : 1 ratio and (b) determined G′ and G″ crossover showing at which strain the gels break. Bar graph represents mean with SEM.

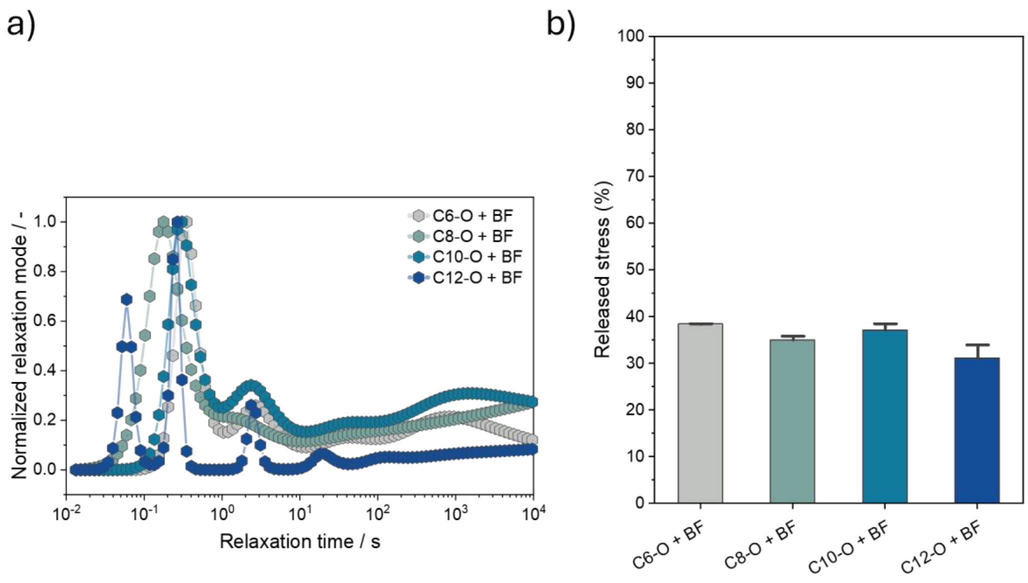

Figure S60. Quantification of stress relaxation for UPy : BF = 9 : 1 ratio. (a) Obtained relaxation spectra from the stress relaxation graph showing most important relaxation scales in the networks. (b) Quantification of released stress after 1000 s relaxation. Bar graph represents mean with SEM.

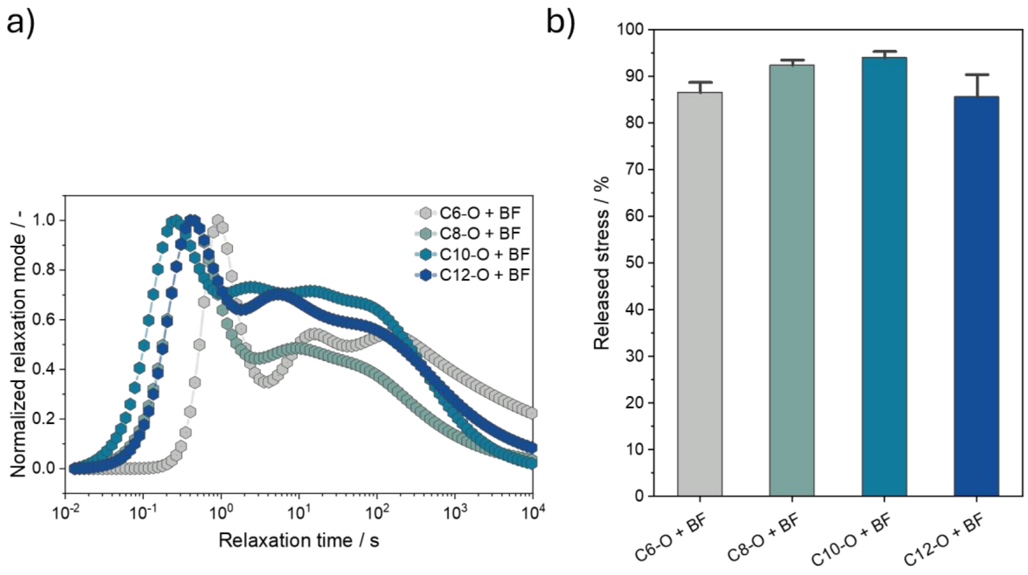

Figure S61. Quantification of stress relaxation for UPy : BF = 1 : 1 ratio. (a) Obtained relaxation spectra from the stress relaxation graph showing most important relaxation scales in the networks. (b) Quantification of released stress after 1000 s relaxation. Bar graph represents mean with SEM.

9 CryoTEM imaging

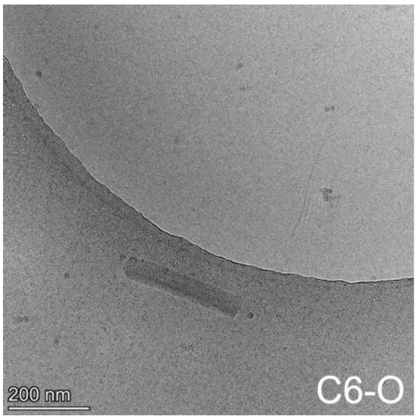

Figure S62. CryoTEM image of **C6-O** (C = 100 µM) in MQ water pointing out the presence of not solely cylindrical fibers (on the ice layer) with a diameter of 6-7 nm but also the presence of larger ribbon-like structures (on the carbon layer) that might be either blotted away or forced apart during the sample preparation owing to their size.

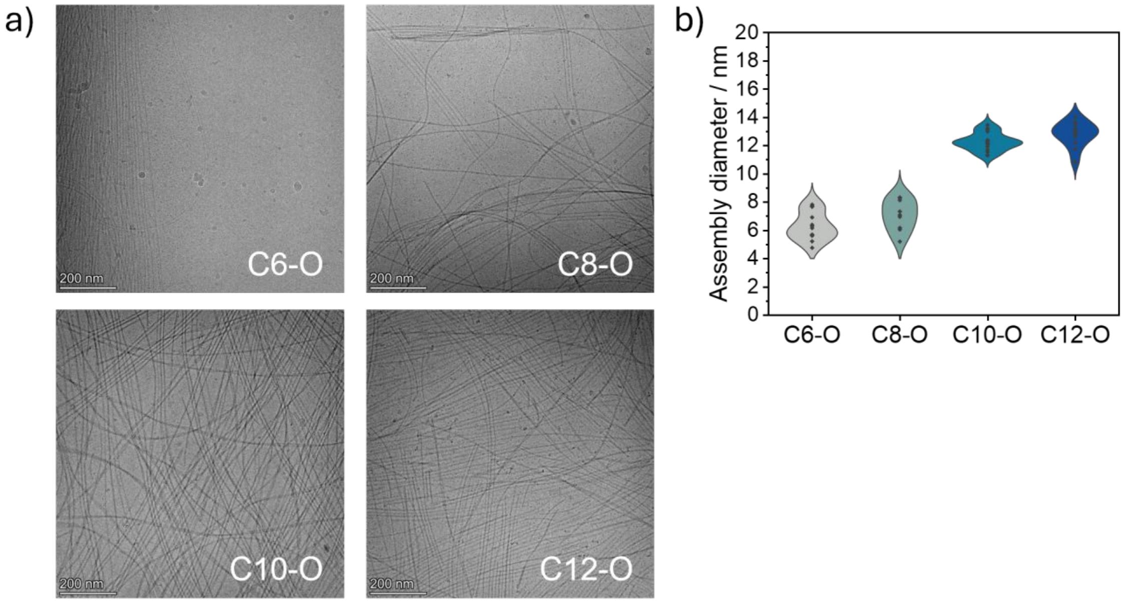

Figure S63. (a) Representative cryoTEM images of **Cn-O** in MQ water at 2.5 mM indicating consistency of the (b) respective fiber diameter with the samples imaged at 100 µM reported in the main text.

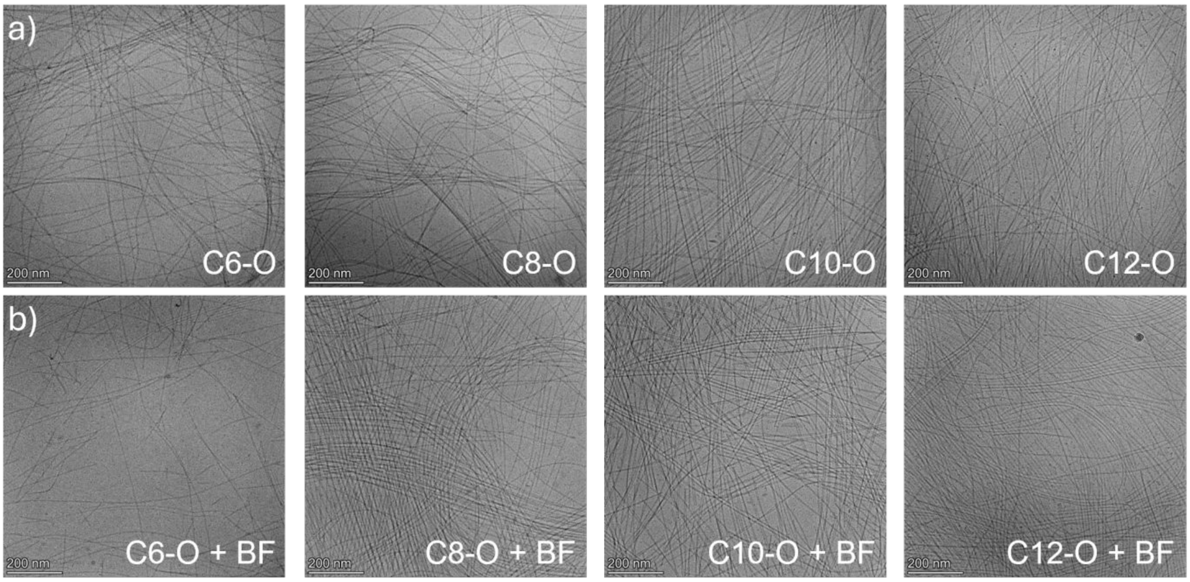

Figure S64. Representative cryoTEM pictures of the gels obtained from each monomer without (a) and with (b) **BF** crosslinker at **UPy**:**BF** = 80:1 in MQ water (C_{tot} = 9.87 mM).

10 3D cell encapsulation experiments

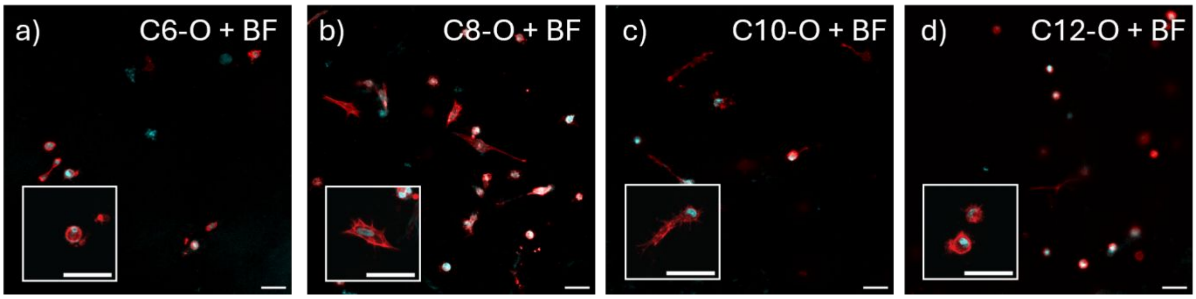

Figure S65. Cells embedded in 3D hydrogels and cultured for 3 days (cytoskeleton; red and nuclei; cyan). All scale bars represent 50 μm.

11 References

(1) Filik, J.; Ashton, A. W.; Chang, P. C. Y.; Chater, P. A.; Day, S. J.; Drakopoulos, M.; Gerring, M. W.; Hart, M. L.; Magdysyuk, O. V; Michalik, S. Processing Two-Dimensional X-Ray Diffraction and Small-Angle Scattering Data in DAWN 2. *J. Appl. Crystallogr.* **2017**, *50*, 959–966.

(2) Pauw, B. R.; Smith, A. J.; Snow, T.; Terrill, N. J.; Thünemann, A. F. The Modular Small-Angle X-Ray Scattering Data Correction Sequence. *J. Appl. Crystallogr.* **2017**, *50*, 1800–1811.

(3) Lou, X.; Schoenmakers, S. M. C.; van Dongen, J. L. J.; Garcia-Iglesias, M.; Casellas, N. M.; Fernández-Castaño Romera, M.; Sijbesma, R. P.; Meijer, E. W.; Palmans, A. R. A. Elucidating Dynamic Behavior of Synthetic Supramolecular Polymers in Water by Hydrogen/Deuterium Exchange Mass Spectrometry. *J. Polym. Sci.* **2021**, *59*, 1151–1161.

(4) Rutten, M. G. T. A.; Rijns, L.; Dankers, P. Y. W. Controlled, Supramolecular Polymer Formulation to Engineer Hydrogels with Tunable Mechanical and Dynamic Properties. *J. Polym. Sci.* **2024**, *62*, 155–164.

(5) Wisse, E.; Spiering, A. J. H.; Dankers, P. Y. W.; Mezari, B.; Magusin, P. C. M. M.; Meijer, E. W. Multicomponent Supramolecular Thermoplastic Elastomer with Peptide-modified Nanofibers. *J. Polym. Sci. Part A Polym. Chem.* **2011**, *49*, 1764–1771.
